# Ti‐Doping Activates Adjacent Zr Sites in Bimetallic MOFs for Cooperative Phospholipid Removals in Human Biomonitoring

**DOI:** 10.1002/advs.76529

**Published:** 2026-07-13

**Authors:** Yanmin Liang, Hongyue Ma, Feier Bai, Jing Zhang, Yan Qi, Bing Shao

**Affiliations:** ^1^ National Key Laboratory of Veterinary Public Health Security College of Veterinary Medicine China Agricultural University Beijing China; ^2^ Beijing Key Laboratory of Diagnostic and Traceability Technologies for Food Poisoning Beijing Center for Disease Prevention and Control Beijing China; ^3^ School of Public Health Capital Medical University Beijing China; ^4^ School of Food and Bioengineering Xihua University Chengdu China

**Keywords:** chemical hazards, matrix interference, nontargeted analysis, phospholipids removal, Zr/Ti bimetallic MOFs

## Abstract

The interference of phospholipids in serum poses a critical bottleneck for nontargeted human biomonitoring of chemical hazards. Here, we report a Ti‐doping strategy to activate adjacent Zr sites in a bimetallic Zr/Ti metal‐organic framework (B‐Zr/Ti MOF) for cooperative and efficient phospholipid removal. By precisely engineering the Ti content, the optimal B‐Zr/Ti MOF achieves near‐complete removal of major phospholipid subclasses while maintaining high recoveries (>50%) for over 500 diverse chemical hazards, even at trace levels (10 ng mL^−^^1^). Combined XPS, FT‐IR, and DFT calculations reveal that Ti incorporation modulates the local electronic environment, converting adjacent Zr sites into highly active Lewis acid centers that synergistically anchor phosphate headgroups via inner‐sphere coordination. When integrated into a nontargeted LC‐HRMS workflow, this material dramatically reduces matrix effects and expands detectable feature coverage. This work establishes bimetallic node engineering as a powerful strategy to overcome matrix interference in exposomics and precision biomonitoring.

## Graphical abstract

Ti‐doped Zr/Ti bimetallic MOFs activate neighboring Zr sites to selectively capture phospholipids from serum through cooperative interactions. In nontargeted screening, the material minimizes matrix interference, expands detectable feature coverage, and enables broad recovery of diverse chemical hazards, providing a powerful cleanup strategy for LC‐HRMS‐based human biomonitoring.

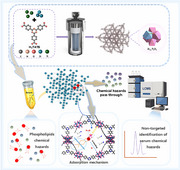

## 1 Introduction

Human biomonitoring (HBM), as the “gold standard” for assessing human exposure to environmental chemicals, is a core component of environmental and health risk assessment. By analyzing chemicals and their metabolites in human specimens (e.g., tissues, blood, urine), HBM provides information on the types, amounts, and levels of exposure in individuals and populations, as well as their spatiotemporal dynamics, and helps elucidate links to health effects [1, 2]. However, with the development of the chemical synthesis industry, a growing number of chemical hazards are being used extensively in people's production and daily life, with a substantial fraction exhibiting potential toxicity and persistence. According to the World Health Organization (WHO) and the United Nations (UN), more than 160 million chemicals are in human use or contact, of which ∼6000 account for 99% of market transactions [3]. Humans are widely exposed to these substances through ingestion of contaminated food and water, inhalation of particulate‐laden air, and dermal contact [4, 5, 6]. Due to the wide range of structural and physicochemical properties covered by these chemical hazards, purely targeted assays offer limited coverage. Accurately screening and identifying these chemical hazards has become a challenge in HBM. Nontargeted screening combined with targeted quantitation raises HBM throughput and coverage while keeping cost in check [7]. Recent advances in high‐resolution mass spectrometry (HRMS) allow simultaneous and reproducible nontargeted analysis of thousands of compounds in biological matrices [8, 9, 10]. However, matrix effects remain a major challenge in MS analysis of biological samples. Sample pretreatment is therefore crucial for accuracy in nontargeted workflows. It should remove interferences without depleting analytes or introducing artifacts [11]. Phospholipids are the major biofilm components and are abundant in biological samples. They are the main source of matrix effects in blood. In LC–HRMS, phospholipids in serum often induce strong matrix effects that mask trace and ultratrace xenobiotics of biological relevance and lead to non‐detection in nontargeted screening [12, 13].

At present, the most common sample preparation for nontargeted screening in plasma or serum is protein precipitation with cold methanol or acetonitrile. Although this approach can extract the largest number of compounds [14], it only provides high recovery and repeatability mainly for medium concentrations of about 800–5000 ng mL^−^^1^ due to the endogenous interference of phospholipids. Current targeted HBM methods mostly use liquid‐liquid extraction, solid‐phase extraction, and removal materials to remove endogenous phospholipids in order to reduce ion suppression [15, 16, 17, 18]. Jade Chaker et al. performed a comprehensive evaluation of 12 sample preparation methods (SPM) using phospholipid and protein removal plates (PLR), solid‐phase extraction plates (SPE), supported liquid extraction cartridge (SLE), and conventionally used protein precipitation (PPT). They found that the preparation method strongly affects both the number of observable compounds and their observable levels [19]. Kalliroi Sdougkou et al. used commercial cartridges with an optimized phospholipid removal step and achieved sensitive multiclass targeted analysis of 77 priority analytes. Even so, when using vendor‐supplied generic phospholipid removal protocols, the recoveries of many analytes after phospholipid removal were low and showed large variability across classes [20].

In recent years, advances in materials science have opened new routes for nontargeted screening of small molecules. Crystalline porous materials such as metal‐organic frameworks (MOFs) and covalent organic frameworks (COFs) have drawn wide attention for their strong performance [21, 22]. MOFs are three‐dimensional crystalline porous networks built from metal nodes and organic linkers. They are designable, thermally stable, highly tunable, and possess high crystallinity, high porosity, and large surface area. By selecting the organic linkers and metal nodes, one can tune surface area, pore size, aperture, and chemical reactivity [23, 24, 25]. Moreover, researchers have shown that synthetic control can tailor many properties of MOFs. As a promising class of sample‐pretreatment materials, MOFs have been developed for nontargeted screening of chemical hazards across diverse matrices [26, 27]. However, MOF sorbents designed specifically for phospholipid removal are still rarely reported. Studies show that MOF adsorbents with single metal active sites often struggle with complex real matrices. The active sites are also tightly confined in the crystal lattice, which limits contact with targets [28]. Introducing a second metal such as Fe, La, or Zn can build bimetallic MOFs. New defects form in the same framework and the two metals act cooperatively. As a result, bimetallic MOFs usually outperform single metal analogues in adsorption [29, 30, 31].

**FIGURE 1:**
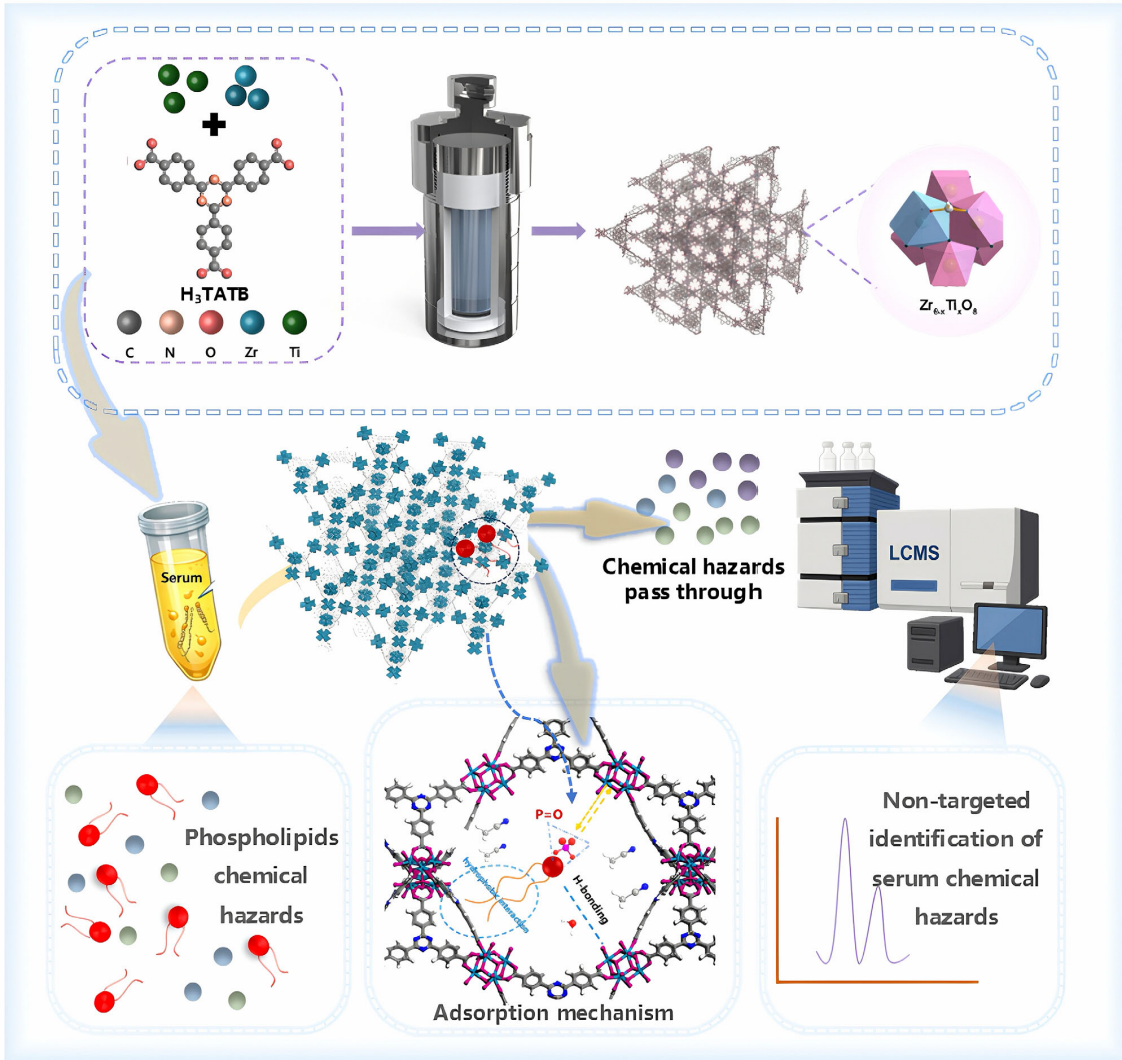
Schematic diagram of synthesis and application mechanism of B‐Zr/Ti MOF.

Zirconium‐based MOFs such as UiO‐66, MOF‐808, and NU‐1000 have drawn wide attention for their high stability and rich structures [32]. PCN‐777 is a Zr MOF with very high porosity and good stability. It is among the MOFs with the largest pore size reported to date. The pore diameter reaches 3.8 nm. The large channels provide ample space for postsynthetic modification [33, 34]. In view of hard acid–base interactions, many hard metal ions such as Fe^3^^+^, Al^3^^+^, Zr^4^^+^, La^3^^+^, and Ti^4^^+^ show strong affinity for phosphate groups [35]. Introducing these metals into PCN‐777 nodes can selectively capture and remove phospholipids and thus lower matrix effects in serum. Based on this idea, the goal of this study was to synthesize a Ti^4^^+^‐doped PCN‐777 bimetallic MOF adsorbent for phospholipid removal from serum and for high recovery of diverse chemical hazards. The prepared Zr/Ti Bimetallic MOF (B‐Zr/Ti MOF) was fully characterized by SEM, XRD, FT‐IR, and XPS. We systematically evaluated its removal performance toward phospholipids and further examined the adsorption mechanism by FT‐IR, XPS, and theoretical calculations (Figure 1). The results showed effective removal of phospholipids from serum and excellent recoveries for more than 500 chemical hazards.

## 2 Materials and Methods

### 2.1 Experimental Materials

Zirconyl chloride octahydrate (ZrOCl_2_·8H_2_O, 99%) was obtained from Macklin. N, N‐dimethylformamide (DMF), N, N‐Diethylformamide (DEF, 99%), 2, 4, 6‐Tris (4‐Carboxyphenyl)‐1,3,5‐Triazine (H_3_TATB, 97%), trifluoroacetic acid (TFA, 99%), Titanium isopropylate were acquired from Aladdin. Acetone was obtained from HUSHI, Agriculture and Veterinary Medicine, and 20 phospholipid standards were purchased in Alta Tianjin. Acetonitrile, isopropanol, formic acid, ammonium formate, ammonium fluoride, and ammonium acetate are all of HPLC grade and purchased from Dikma Technologies Inc., Fetal bovine serum was purchased from PAN Seratech. Human serum used for method validation was obtained from a Beijing population‐based health cohort recruited between August 2017 and November 2017. All participants were informed about the study, and the study protocol was approved by the Ethics Review Committee of the Beijing Center for Disease Prevention and Control (No. 2017D(6)).

### 2.2 Preparation of Ti/Zr Bimetallic‐Based MOFs

Synthesis of PCN‐777 was done to follow the reported protocols [36]. Briefly, 90 mg H_3_TATB and 360 mg ZrOCl_2_·8H_2_O were dissolved in 12 mL DEF, followed by adding 0.6 mL of TFA to continue sonication. The clarified solution was heated under vacuum oven at 120°C for 12 h. After cooling to room temperature, a white powder product of PCN‐777 was collected. The crystallized products were washed with 50 mL DMF and 50 mL acetone, and then the white solids were dried at room temperature for 12 h.

Preparation of B‐Zr/Ti MOF: B‐Zr/Ti MOF was synthesized in the same way as PCN‐777. Different proportions of ZrOCl_2_·8H_2_O and titanium isopropoxide (n_Zr_+n_Ti_ = 1 mmol), and 90 mg H_3_TATB were dissolved in 12 mL DEF. Finally, 0.6 mL of TFA was added to continue sonication for 30 min. The clarified solution was heated under vacuum oven at 120°C for 12 h. After waiting for natural cooling, the crystallized products were washed with 50 mL DMF and 50 mL acetone, and then the white solids were dried at room temperature for 12 h. The final sample was designated as B‐Zr/Ti MOF (where Ti/Zr = 5%, 10%, 25%, 50%).

### 2.3 Characterization of Adsorbents

Various structural parameters of the B‐Zr/Ti MOF were determined by different means of characterization and analysis. The microscopic morphology and the elemental composition of their surfaces were observed using a Scanning Electron Microscope‐Energy Spectrometer (SEM‐EDS). The pore structure characteristics of the B‐Zr/Ti MOF were analyzed by the specific surface area and porosity analyzer (QuadrasorbSI) at 77 K after vacuum treatment at 100°C for 12 h. X‐ray diffractograms (XRD) were measured in the range of 2–30° using an x‐ray diffractometer (Rigaku SmartLab SE, Japan) at a speed of 5°/min. Fourier Transform Infrared Spectroscopy (FT‐IR) was utilized to identify characteristic functional groups within the adsorbent. X‐ray Photoelectron Spectroscopy (XPS) analyzed the chemical environment surrounding specific elements.

### 2.4 Removal of Phospholipid by B‐Zr/Ti MOF

Serum samples are rich in diverse phospholipids, with phosphatidylcholine (PC), sphingomyelin (SM), and lysophosphatidylcholine (lyso‐PC) collectively accounting for approximately 80%–90% of the total phospholipid content in plasma [37]. Due to their amphiphilic nature, these phospholipids are frequently co‐extracted with target analytes during sample preparation, leading to significant matrix effects [12, 38]. To evaluate the adsorption and desorption capabilities of B‐Zr/Ti MOF for phospholipids in serum, fetal bovine serum was selected as a model sample, and acetonitrile was employed as the solvent for protein precipitation. Specifically, 200 µL of the serum sample was transferred into a 1.5 mL centrifuge tube. Following the addition of 800 µL of acetonitrile, the mixture was vortexed for 30 s and subsequently subjected to ultrasonication for 5 min. The mixture was then centrifuged at 12 000 rpm for 5 min at 4°C to precipitate proteins. The resulting organic supernatant was collected and reserved for subsequent experiments.

Varying amounts of ground B‐Zr/Ti MOF were added to 500 µL of acetonitrile and ultrasonicated for 1 min to achieve a homogeneous dispersion. Then, 500 µL of the aforementioned serum extract was introduced into the material dispersion, and the mixture was vortexed for several minutes. After centrifugation at 10 000 rpm for 3 min, the supernatant was collected for analysis. The removal efficiency of phospholipids was assessed by quantifying PCs, LPCs, and SMs using ultra‐high performance liquid chromatography‐mass spectrometry (UPLC‐MS/MS) in positive ion mode. This was achieved by monitoring the phosphocholine head group fragment ion (exact mass ∼184.0733, C_5_H_15_NO_4_P^+^) via a precursor ion scan (PIS) of m/z = 184. Additionally, the removal efficiency of B‐Zr/Ti MOF for 20 representative phospholipids was evaluated using multiple reaction monitoring (MRM) mode. The retention times (t) for these 20 phospholipids are provided in Table S2.

To evaluate the selective removal of phospholipids on B‐Zr/Ti_10%_ MOF, a mixed standard solution of 35 organophosphate esters, each at 10 µg kg^−1^, was spiked into serum. Sample pretreatment was then performed as described in the above method, followed by UPLC–MS/MS analysis to assess the selectivity of the adsorbent toward phospholipids. The specific instrumental analysis method of organophosphorus is based on the previous work of our research [39].

Various solvents were selected for phospholipid elution, and the elution efficiencies of different eluents were compared. Specifically, the phospholipid‐adsorbed material was redispersed in 1 mL of various organic solutions, followed by ultrasonication for 3 min and subsequent centrifugation at 10 000 rpm for 3 min. The resulting eluate was collected for analysis. The reproducibility across different batches of B‐Zr/Ti _10%_ MOF was evaluated by comparing the removal efficiency of five independently synthesized batches. The stability of B‐Zr/Ti _10%_ MOF was preliminarily assessed by monitoring the changes in removal efficiency over periods of 1, 5, 10, 15, and 30 days. Subsequently, its stability under different pH conditions (0.1 mol/L HCl, pH   1; water, pH   7; 0.1 mol/L NaOH, pH   13) was further examined. The phospholipid content in the eluate was determined using the method described above.

The phospholipid removal efficiency (%) and the elution efficiency of the adsorbed phospholipids (Ee%) were calculated using the following Equations (1) and (2):

(1) $$\mathrm{Removal}\;\mathrm{efficiency}\;\%=\frac{A_{0}-A_{e}}{A_{0}}\;\times 100\%$$

(2) $$\mathrm{Ee}=\frac{A_{d}\times V_{d}}{A_{0}\times V}\;\times 100\%\;$$

where A_0_ and A_e_ represent the initial and equilibrium peak areas of the phospholipids, respectively; A_d_ denotes the peak area of the phospholipids in the eluate; V (mL) and V_d_ (mL) are the volumes of the solution in contact with the adsorbent and the eluent, respectively.

### 2.5 Adsorption Experiment

B‐Zr/Ti _10%_MOF was accurately weighed and put in phospholipid solution. After the material was evenly dispersed by ultrasound, the mixture was placed on a shock mixer with a velocity of 1500 rpm. After adsorption saturation, the reaction mixture was centrifuged, and the supernatant was detected by LC‐MS to determine the residual phospholipid concentration. The following equation (Equation 3) was used to calculate the adsorption capacity (q_e_, exp mg g^−1^).

(3) $$q_{e}=\frac{(C_{0}-C_{e})V}{m}$$

C_0_ and C_e_ (mg L^−1^) represent the initial and equilibrium concentrations of the PC solution, respectively. V (L) is the volume of the phospholipid solution, and m (g) is the mass of B‐Zr/Ti _10%_ MOF.

#### 2.5.1 Adsorption Kinetics

At a constant temperature, Samples were collected at from 0 to 15 min and were subsequently analyzed for concentration. The obtained data were then fitted using the pseudo‐first‐order (Equation 4) and pseudo‐second‐order (Equation 5) adsorption kinetic models.

(4) $$\ln(q_{e}-q_{t})=\ln q_{e}-k_{1}t\;$$

(5) $$\frac{t}{q_{t}}=\frac{t}{q_{e}}+\frac{1}{k_{2}q_{e}^{2}}$$

where t (min) represents the adsorption reaction time, q_t_ (mg g^−1^) and q_e_ (mg g^−1^) represent the phospholipid adsorption capacity of B‐Zr/Ti _10%_ MOF at t and equilibrium time, respectively. k_1_ (min^−1^) and k_2_ (g mg^−1^ min^−1^) represent the rate constants of the pseudo‐first and pseudo‐second‐order models, respectively.

#### 2.5.2 Adsorption Isotherm

Phospholipid solutions were initially prepared with varying concentrations. Adsorption equilibrium was achieved through constant‐temperature adsorption, samples were subsequently analyzed for concentration. The experimental data were subjected to fitting using Langmuir (Equation 6) and Freundlich (Equation 8) isothermal adsorption models:

(6) $$\frac{C_{e}}{q_{e}}=\frac{C_{e}}{q_{m}}+\frac{1}{k_{L}q_{m}}$$

(7) $$R_{L}=\frac{1}{1+k_{L}C_{0}}$$

(8) $$\log\;q_{e}=\frac{1}{n}\;\log C_{e}+\log k_{F}$$

where q_m_ (mg g^−1^) represents the maximum adsorption amount of lipid, k_L_ (L mg^−1^) and k_F_ (mg L^−1^) represent the constants of the Langmuir and Freundlich isotherm model, respectively, n represents the empirical constant, RL represents the dimensionless factor.

### 2.6 DFT Calculation and Analysis

The framework of the material was fixed during the sampling process. A set of low‐energy initial adsorption conformations within a 6 kcal/mol window was obtained using the GFN‐FF force field and the NCI conformational sampling method. These conformations were then coarsely optimized with the GFN1‐xTB method in xTB, yielding the lowest‐energy conformation among the adsorption structures. Subsequently, DFT‐level optimization and frequency calculations were performed on the pre‐optimized structures using the B3LYP‐D3/def2‐SVP method in Gaussian 16 A.03, resulting in precise structures of phospholipid adsorption. Single‐point energy calculations for the phospholipid, MOF material, and adsorption structures were conducted at the B3LYP‐D3/def2‐TZVP level to obtain high‐precision adsorption energies. Charge transfer between the phospholipid headgroup and the MOF metal sites was estimated using Natural Bond Orbital (NBO) natural charge analysis based on the optimized adsorption complexes. The charge‐transfer magnitude was calculated from the variation in NBO natural charges of the interacting fragments/sites and is reported in units of elementary charge (e).

### 2.7 Nontargeted Analysis Verification of Chemical Hazards in Serum

The purification method optimized here, involving phospholipid removal, was contrasted with a control method without phospholipid removal, as is typical for metabolomics. The methods are hereafter simply termed “After” and “Before” and were contrasted by preparing three aliquots of 200 µL pooled serum per method. Plasma aliquots were first placed in 2 mL propylene tubes and fortified with a labelled internal standard mixture of 10 substances in MeOH. Protein precipitation was achieved by adding 800 µL ACN at room temperature and vortexing for 20 s. After solvent addition, all samples ultrasonicated at 4°C for 5 min, then centrifuged at same temperature, at 12 000 rpm for 10 min. Thereafter, 10 mg of B‐Zr/Ti_10%_ MOF material was dispersed in 500 µL acetonitrile under ultrasonic treatment for 1 min. The spiked supernatant (500 µL) was then added to the MOF dispersion, vortexed for 30 s, and centrifuged at 10 000 rpm for 3 min to separate B‐Zr/Ti MOF. Finally, extracts were analyzed by ultrahigh‐pressure LC with HRMS acquisition in positive and negative electrospray ionization mode.

### 2.8 Targeted Analysis Verification of Chemical Hazards in Serum

In the nontarget analysis and verification of chemical hazards, 200 µL serum samples were transferred to 1.5 mL polypropylene centrifuge tubes. Then, 800 µL of acetonitrile was added, and the mixture was vortexed for 30 sec using a Thermo Fisher Scientific vortex mixer, followed by ultrasonic treatment for 5 min. Subsequently, the sample was centrifuged at 12 000 rpm at 4°C for 5 min, and the resulting supernatant was collected for further processing. Then the standard stock solutions of 500 representative chemical hazards were added to 500 µL supernatant (final concentrations were 10µg kg^−1^). Thereafter, 10 mg of B‐Zr/Ti_10%_ MOF material was dispersed in 500 µL acetonitrile under ultrasonic treatment for 1 min. The spiked supernatant (500 µL) was then added to the MOF dispersion, vortexed for 30 s, and centrifuged at 10 000 rpm for 3 min to separate B‐Zr/Ti_10%_ MOF. Finally, the supernatant was analyzed by UPLC‐MS/MS.

### 2.9 Method Performances

In order to evaluate the efficiency of sample purification and instrumental analysis methods, we measured the matrix effect (ME), accuracy, and precision. For all target chemicals, the matrix effect was calculated by the ratio of the peak area of matrix to pure solvent at three spiked levels. The accuracy and precision were expressed by the recovery rate and relative standard deviation percentage (RSD%) of each target chemical in the repeated determination of the spiked samples.

## 3 Results and Discussion

### 3.1 Preparation and Structural Characterization of B‐Zr/Ti MOF

The adsorption performance is inherently influenced by the morphology and structural properties of the adsorbent. SEM characterization revealed a pronounced morphological evolution of PCN‐777 upon Ti incorporation. As shown in Figure S1, the pristine sample (0%) exhibited well‐defined octahedral crystals with smooth surfaces and uniform particle sizes of approximately 1–2 µm, reflecting its highly crystalline framework [40]. With 5% Ti doping, the octahedral morphology was largely preserved, although slight surface wrinkling appeared, suggesting mild lattice distortion and defect formation induced by Ti^4^^+^ substitution. When the Ti content increased to 10%, partial crystal collapse occurred, accompanied by a roughened surface decorated with more small nanoparticles. The overall geometric outline, however, remained discernible, indicating that moderate Ti incorporation partially replaced Zr^4^^+^ within the Zr_6_ clusters. Owing to the smaller ionic radius and distinct coordination preference of Ti^4^^+^, local lattice distortions were generated, giving rise to coordinatively unsaturated sites (CUSs). These structural defects may effectively modulate the local electronic environment, enhancing the Lewis acidity and polarization capability of the framework [41]. In contrast, further increasing the Ti content to 25% and 50% resulted in pronounced structural degradation and amorphous aggregation, implying that excessive Ti^4^^+^ disrupts the coordination self‐assembly process and hinders MOF crystallization.

**FIGURE 2:**
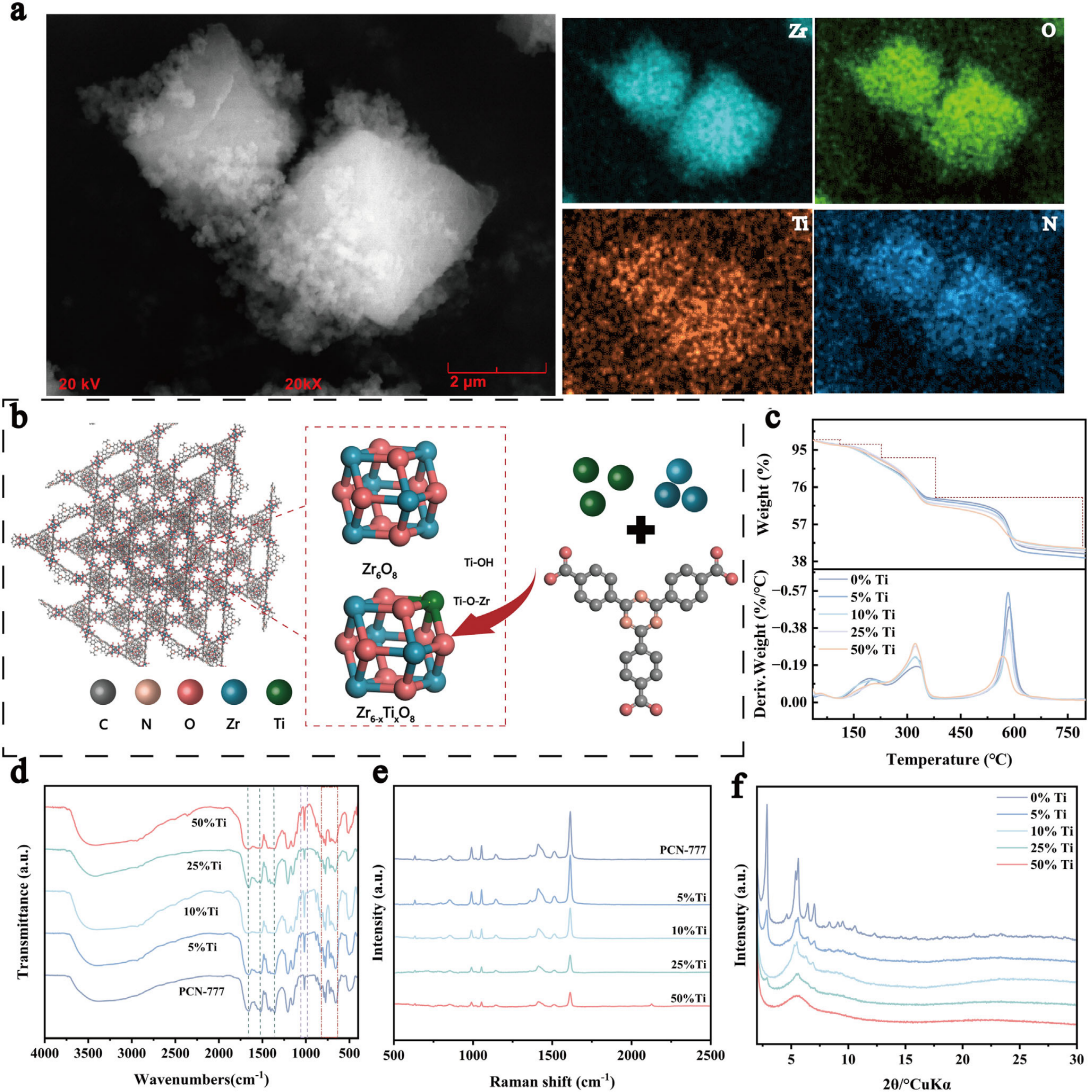
(a) EDS micrographs of Zr, O, Ti, and N in B‐Zr/Ti_10%_ MOF. (b) Schematic illustration of the formation mechanism. (c) TG curves. (d) FTIR spectra. (e) Raman spectra and (f) XRD patterns of B‐Zr/Ti MOF.

Elemental mapping (Figure 2a) further confirmed the uniform distribution of Zr, O, Ti, and N in the B‐Zr/Ti_10%_ MOF. The spatial overlap between Ti and Zr signals demonstrated the successful incorporation of Ti^4^^+^ into the Zr_6_ clusters rather than the formation of an independent TiO_2_ phase. The homogeneous dispersion of Ti within the framework suggests the formation of mixed Ti─O─Zr nodes, while the N element derived from the H_3_TATB linkers exhibited a consistent distribution, verifying the integrity of the organic–inorganic coordination network. The smaller ionic radius of Ti^4^^+^ results in a higher charge density. As a Lewis acid (an electron pair acceptor), the central metal ion exhibits a stronger ability to attract electron pairs, thereby demonstrating enhanced acidity [42]. Consequently, we propose that such structural modulation finely tunes the local electronic environment and enhances the Lewis acidity of the metal nodes. This enhancement is expected to promote interactions with phospholipid molecules, thereby improving both the adsorption affinity and selectivity of the adsorbent.

Figure 2b schematically illustrates the proposed formation mechanism of B‐Zr/Ti MOF. The pristine Zr_6_O_8_ clusters act as the SBUs in the framework, while a fraction of Zr^4^^+^ centers can be substituted by Ti^4^^+^ during the solvothermal self‐assembly with the tritopic H_3_TATB linker. The resulting Zr_6__−__x_Ti_x_O_8_ nodes are bridged through Ti─O─Zr bonds, forming mixed‐metal clusters with slightly distorted local coordination. Such substitution modulates the electronic environment of the metal nodes, introduces lattice defects, and enhances the Lewis acidity of the framework.

The thermogravimetric curve directly demonstrates the excellent stability of Ti‐doped PCN‐777. As shown in Figure 2c, the initial weight loss below 200°C accounted for 6.9–9.3 wt.% for all samples. This minor loss was mainly attributed to the evaporation of various solvent molecules (DEF, DMF, and acetone) [43]. In the 200–400°C range, a small but identifiable weight loss occurs in all samples. This process is primarily due to the gradual removal of strongly adsorbed or strongly bound solvents or modulators, as well as the dehydroxylation of µ_3_‐OH groups on Zr_6_O_8_ (or Zr_6__−__x_Ti_x_O_8_) clusters [44]. A corresponding slight shoulder peak was also discernible in the DTG curve around 280–350°C. Upon Ti incorporation, this peak became moderately more pronounced and broadened, suggesting a widened distribution of bonding energies associated with the ─OH groups surrounding the mixed‐metal nodes, as well as an increased density of transferable hydroxyl groups. The weight loss rate in the 450–600°C range (52.7–57.7 wt.%) was attributed to the collapse of the adsorbent framework, related to the decomposition of the organic ligand. The rate of mass loss slightly changes with increasing Ti content, indicating that Ti substitution affects the bonding strength of the organic ligands. When the Ti content increases, the mass loss rate stabilizes above 600°C, suggesting that a higher proportion of inorganic oxides exists in the stable Ti─Zr oxide form. Additionally, no extra mass loss steps occur at lower temperatures, indicating that the Ti binding has not introduced loosely bound substances or unstable coordinated water.

The FT‐IR spectra of Ti‐doped B‐Zr/Ti MOF samples reveal the coordination environment of the metal nodes and the interactions between the metal centers and the organic linkers. As shown in Figure 2d, the characteristic FT‐IR peaks of B‐Zr/Ti MOF exhibit remarkable similarity to the pristine PCN‐777. All samples retained the characteristic vibrational features of the triazine–carboxylate junctions, with adsorption bands at 1358, 1403, and 1654 cm^−^^1^ assigned to the asymmetric and symmetric stretching vibrations of the ─COOH groups. The adsorption peak at 1521 cm^−^^1^ originates from the triazine moiety in PCN‐777 [45]. The characteristic peaks observed at 658 and 774 cm^−^^1^ correspond to the stretching vibrations of Zr─O bonds. With the increasing of Ti content, the M─O vibration peaks slightly shift due to the weaker Ti─O bond compared to Zr─O, resulting in a lower vibrational frequency and suggesting the possible formation of Ti─O─Zr linkages [46]. Furthermore, the absence of a characteristic TiO_2_ lattice vibration near 520 cm^−^^1^ further excludes the presence of crystalline TiO_2_ impurities. These results collectively confirmed that Ti was incorporated into the framework nodes rather than existing as an external oxide phase.

The Raman spectra of B‐Zr/Ti MOF samples provide insights into the structural integrity and local environment around the metal centers upon Ti incorporation. As shown in Figure 2e, the pristine PCN‐777 exhibits two main peaks at 1610 and 1513 cm^−^^1^, corresponding to the C═C stretching vibrations of the triazine rings in the TATB linkers. The band at 1411 cm^−^^1^ is assigned to the coupled C–N/C–C stretching of the triazine ring [47]. These characteristic peaks confirm the preservation of the aromatic framework of the linkers in PCN‐777. The persistence of these vibrational modes in all Ti‐doped samples indicates that the π‐conjugated structure of the organic linkers remains intact after metal substitution. With increasing Ti content, the intensities of the peaks at 1610, 1513, and 1380 cm^−^^1^ gradually decrease, suggesting the introduction of disorder or defects within the aromatic TATB linkers. This implies that Ti incorporation may distort the conjugated system of the TATB moieties, thereby reducing the overall structural order of the framework. In the low‐frequency region (600–700 cm^−^^1^), a small band appears at 632 cm^−^^1^, which can be attributed to the Zr─O vibrational modes within the metal clusters [44]. As the Ti content increases, these peaks become broader and less intense, indicating enhanced structural disorder and defect formation induced by Ti doping.

The PXRD patterns were employed to characterize the structural features of B‐Zr/Ti MOF. As shown in Figure 2f, PCN‐777 exhibits strong diffraction peaks at approximately 2.88°, 5.60°, 6.46°, 7.02°, and 9.50°, which are consistent with its reported structure [48]. The PXRD patterns of B‐Zr/Ti MOF with different Ti contents display high similarity to that of pristine PCN‐777 in both crystallinity and phase purity. The undoped PCN‐777 shows distinct low‐angle reflections (2θ ≈ 4–9°) characteristic of its large‐pore cubic structure. The B‐Zr/Ti_5%_ MOF and B‐Zr/Ti_10%_ MOF retained the same set of low‐angle peaks, with only minor attenuation and peak broadening, indicating that the PCN‐777 topology is well preserved. At higher Ti concentrations, however, the first low‐angle reflection becomes noticeably broadened and weakened, accompanied by an increase in diffuse background intensity, suggesting reduced crystallinity and partial amorphization of the framework. In addition, the main low‐angle peaks shift slightly toward higher 2θ values as the Ti content increases, which is consistent with moderate lattice contraction caused by partial substitution of the larger Zr^4^^+^ ions with smaller Ti^4^^+^ ions in the metal cluster nodes [41, 49, 50]. Importantly, no diffraction maxima corresponding to anatase TiO_2_ (e.g., 2θ ≈ 25.3° and 37.8°, Cu Kα) are observed after Ti doping, further confirming that no TiO_2_ phase is formed. Therefore, Ti can be effectively incorporated into the Zr_6_O_8_ nodes to form mixed‐metal Zr_6__−__x_Ti_x_O_8_ clusters, while excessive Ti introduction leads to framework distortion and partial collapse, consistent with the EDS and Raman results.

**FIGURE 3:**
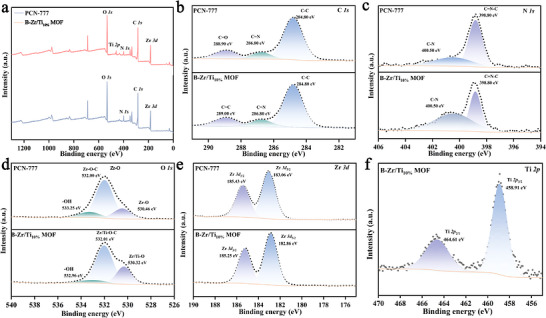
XPS spectra of PCN‐777and B‐Zr/Ti_10%_ MOF (a) survey, (b) C1 s, (c) N 1 s, (d) O 1 s, (e) Zr 3d and (f) Ti 2p.

The XPS spectra (Figure 3) was performed to investigate the bonding states, valence states, and coordination environment of the elements in B‐Zr/Ti MOF. The survey spectra of pristine PCN‐777 and B‐Zr/Ti_10%_ MOF (Figure 3a,f) clearly show the presence of C 1s, N 1s, O 1s, and Zr 3d signals, while an additional Ti 2p doublet is observed for B‐Zr/Ti_10%_ MOF, confirming the successful incorporation of Ti into the framework. The C 1s spectra (Figure 3b) exhibit three distinct peaks centered at 284.8 eV (C─C), 286.8 eV (C─N), and 289.0 eV (O─C═O/C═O), corresponding to the characteristic bonds (C─C, C─N, and C═O) within the triazine‐carboxylate linkers [51]. The N 1s spectrum (Figure 3c) shows a peak at approximately 398.8 eV, assigned to the CɐN─C group, along with a shoulder at around 400.5 eV corresponding to C─N, further confirming the intact triazine structure [52]. Three characteristic peaks are observed in PCN‐777. The peak at 530.46 eV is assigned to Zr─O bonds, the peak at 532.00 eV corresponds to Zr─O─C species, and the peak at 533.25 eV is attributable to weakly bound oxygen species (e.g., H_2_O and hydroxyl groups, OH). In contrast, for the B‐Zr/Ti_10%_ MOF, partial substitution of Zr by Ti leads to a shift of the binding energies of lattice oxygen (Zr/Ti–O), surface oxygen (Zr/Ti─O─C), and weakly bound oxygen species (OH) toward lower values, indicating charge redistribution upon Ti doping [53]. The Zr 3d spectra of both MOFs confirm the presence of Zr^4^^+^ species (Figure 3e). Moreover, the Zr 3d peaks in B‐Zr/Ti_10%_ MOF exhibit a slight shift (≈0.2 eV) toward lower binding energy compared to pristine PCN‐777, suggesting a slight increase in electron density at the Zr sites due to electronic interactions within the mixed Zr_6__−__x_Ti_x_O_8_ nodes [54]. Previous studies on mixed Zr–Ti oxides also attributed such shifts to the formation of Zr─O─Ti bonds [55].

**FIGURE 4:**
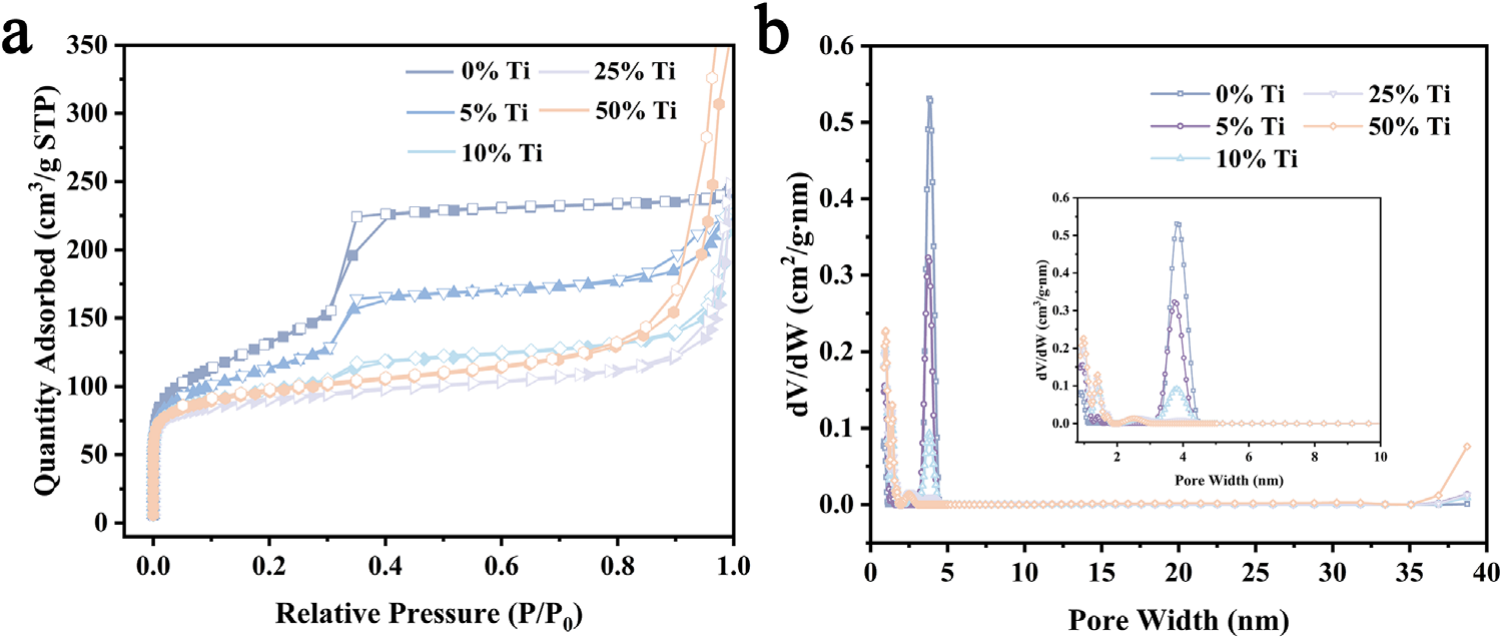
(a) Nitrogen adsorption–desorption isotherms at −196°C and (b) pore size distribution curves of different adsorbents.

The N_2_ adsorption–desorption isotherms of PCN‐777 and B‐Zr/Ti MOF are shown in Figure 4a. All adsorbents exhibit type IV isotherms with a distinct hysteresis loop at high relative pressure (P/P_0_ = 0.8–1.0), characteristic of mesoporous materials [56]. As shown in Table 1, the Brunauer–Emmett–Teller (BET) surface area of pristine PCN‐777 is 471.8 m^2^ g^−^^1^. Upon Ti incorporation (5% and 10%), the overall shape of the isotherms remains nearly unchanged, but the N_2_ uptake decreases noticeably, indicating a significant reduction in microporosity and BET surface area. At higher Ti contents, both the isotherm shape and removal capacity are markedly altered, which can be attributed to partial framework distortion and decreased crystallinity induced by Ti substitution. These structural changes lead to the loss of the ordered pore channels typical of metal–organic frameworks, consistent with the XRD results. The pore size distribution profiles (Figure 4b) show that all samples have a dominant pore size centered at approximately 3.5–4.0 nm [56], corresponding to the intrinsic mesopores of PCN‐777. The presence of a sharp and intense peak observed for pristine PCN‐777 signifies a uniform pore size distribution. With increasing Ti content, the main peak progressively broadens and decreases in intensity, reflecting the generation of structural disorder and partial pore blocking. At high Ti loadings (≥25%), a small additional contribution appears in the macropore region (>30 nm), which is likely associated with interparticle voids or framework collapse. In addition, the pore peaks of 25% and 50% Ti samples at 0–2 nm may be due to the defects of high doping, which improve MOF, resulting in partial disorder and defect micropores in the skeleton wall. Overall, these results indicate that moderate Ti incorporation (≤10%) can be achieved without significant loss of porosity, whereas excessive Ti doping leads to reduced surface area, partial amorphization, and pore structure heterogeneity. These findings are consistent with the TGA and XRD analyses, further confirming the successful incorporation of Ti into the PCN‐777 framework.

**TABLE 1:** Pore structure parameters calculated from the N_2_ adsorption–desorption isotherms.

Adsorbents	S_BET_ (m^2^/g)	Total pore volume (cm^3^/g)	Average pore size (nm)
PCN‐777(_0%Ti‐Zr_)	471.8153 m^2^/g	0.366131 cm^3^/g	3.1040 nm
PCN‐777(_5%Ti‐Zr_)	391.5661 m^2^/g	0.351709 cm^3^/g	3.5928 nm
PCN‐777(_10%Ti‐Zr_)	351.2153 m^2^/g	0.362119 cm^3^/g	4.1242 nm
PCN‐777(_25%Ti‐Zr_)	332.1914 m^2^/g	0.383821 cm^3^/g	4.6217 nm
PCN‐777(_50%Ti‐Zr_)	313.9508 m^2^/g	0.551965 cm^3^/g	7.0325 nm

### 3.2 Optimization of Adsorption and Desorption Processes

**FIGURE 5:**
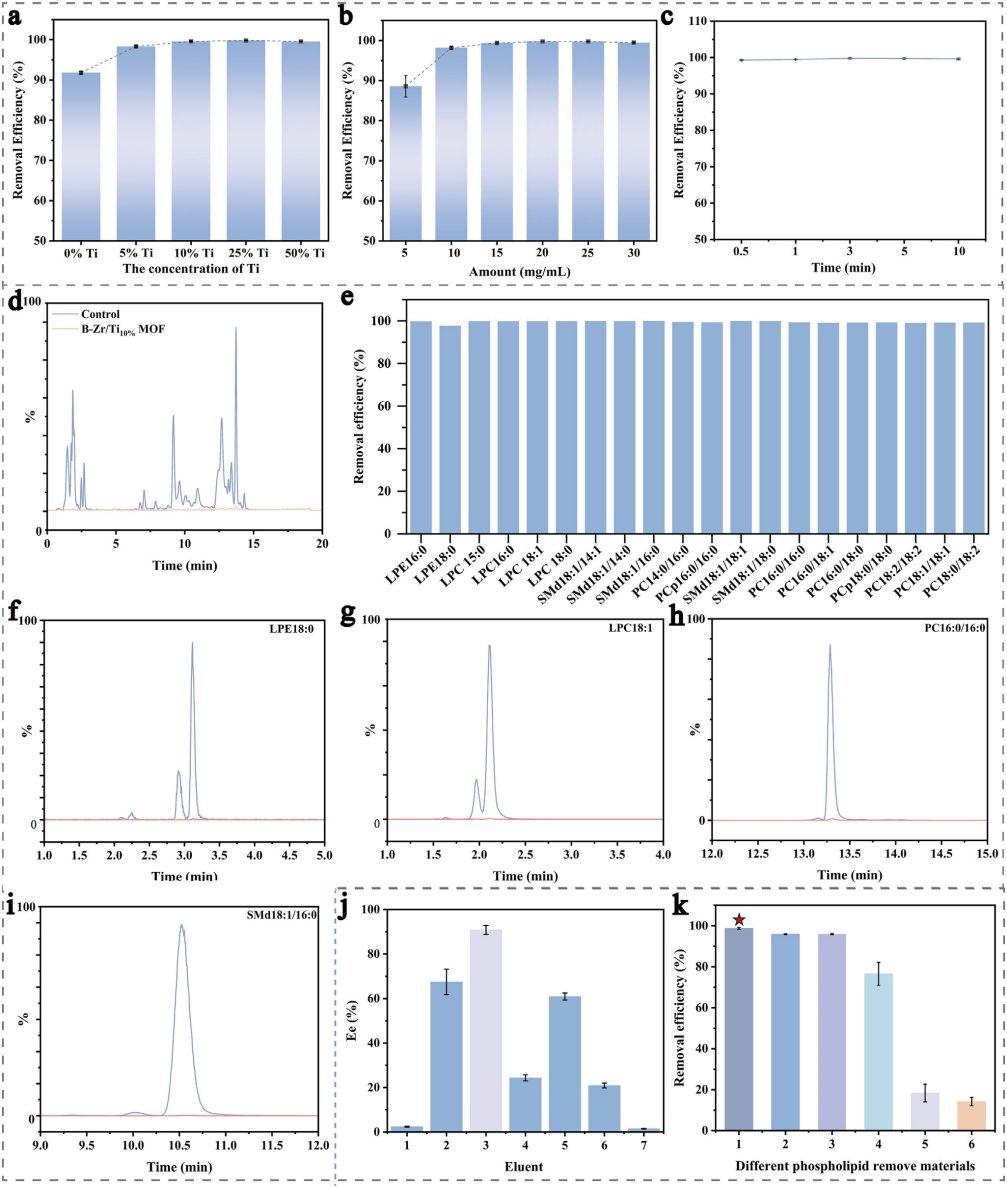
(a) Removal efficiency of phospholipids by B‐Zr/Ti_x_ MOF (x = 0%, 5%, 10%, 25%, 50%) (*n* = 3). (b) Removal efficiency of Phospholipid after treatment with different amounts of B‐Zr/Ti_10%_ MOF (*n* = 3). (c) Removal efficiency of Phospholipid after the addition of 10 mg mL^−1^ B‐Zr/Ti_10%_ MOF with different removal times (*n* = 3). (d) LC chromatograms of phospholipid removal performance of B‐Zr/Ti_10%_ MOF (m/z = 184.0). (e) Removal efficiency of B‐Zr/Ti_10%_ MOF for 20 different phospholipids. LC Chromatogram of removal performance of B‐Zr/Ti_10%_ MOF for (f) LPE, (g) LPC, (h) PC, and (i) SM. (j) Phospholipid elution efficiency of different elution solvents 1–7 correspond to acetonitrile, chloroform/methanol/ammonia (75/20/5, v/v/v), 7 M ammonia in methanol, n‐hexane/isopropanol (2/8, v/v), chloroform/methanol/water (3/5/2, v/v/v), n‐hexane/dichloromethane (4/1, v/v), and methanol, respectively (*n* = 3). (k) Comparison between B‐Zr/Ti_10%_ MOF and different commercial materials (*n* = 3). *Note*: Data are presented as mean ± SD.

To achieve optimal adsorption and desorption performance for phospholipid removal, the key parameters—including adsorbent dosage, adsorption time, desorption solvent, and reusability—were systematically evaluated and optimized. As shown in Figure 5a, the phospholipid removal efficiency increases with Ti content in B‐Zr/Ti_x_ MOF (x = 0%, 5%, 10%, 25%, and 50%). The removal efficiency reaches its maximum (>99%) at a Ti doping of 10%. However, when the Ti content was further increased to 50%, a slight decline in efficiency was observed, which can be attributed to partial framework collapse and a concomitant reduction in the number of accessible active sites. Therefore, B‐Zr/Ti_10%_ MOF was selected for subsequent optimization experiments. To determine the optimal adsorbent dosage, various amounts of B‐Zr/Ti_10%_ MOF were added to 1 mL of acetonitrile extracts from serum samples. As shown in Figure 5b, the phospholipid removal efficiency reached approximately 90% at an adsorbent dosage of 5 mg mL^−^^1^ and increased further with increasing dosage. A plateau was attained at 10 mg mL^−1^, where the removal efficiency stabilizes at 99.4%. Consequently, a dosage of 10 mg mL^−1^ was selected for subsequent experiments. Phospholipid removal is a time‐dependent process; therefore, the removal equilibrium time was optimized. As shown in Figure 5c, when the adsorbent is mixed with the serum for 0.5 min, a removal efficiency of 99.26% is achieved. Extending the contact time up to 10 min does not lead to further improvement, suggesting that equilibrium is reached rapidly. Considering the practical constraints of sample pretreatment, a removal time of 30 s was chosen for subsequent experiments.

Under the optimized adsorption conditions, B‐Zr/Ti_10%_ MOF exhibits excellent phospholipid removal performance in serum samples (Figure 5d). In addition to total phospholipid removal, the material also demonstrates outstanding efficiency toward various phospholipid species. The removal efficiencies for 20 different phospholipids all exceed 99% (Figure 5e). Furthermore, The LC chromatograms (Figure 5f–i) show that B‐Zr/Ti_10%_ MOF effectively removes LPE, LPC, PC, and SM., confirming the high efficacy of B‐Zr/Ti_10%_ MOF for phospholipid removal.

In order to explore the elution efficiency of phospholipids on B‐Zr/Ti_10%_ MOF by different solvents, the desorption efficiency of several solvent systems was tested and compared, including acetonitrile, chloroform/methanol/ammonia (75/20/5, *v*/*v*/*v*), 7 M ammonia in methanol, n‐hexane/isopropanol (2/8, *v*/*v*), chloroform/methanol/water (3/5/2, *v*/*v*/*v*), n‐hexane/dichloromethane (4/1, *v*/*v*), and methanol (Figure 5j). Among these, the 7 M ammonia in methanol exhibited the most satisfactory desorption efficiency for phospholipids in serum samples and was thus selected as the optimal eluent. Subsequently, the reusability of the adsorbent after elution was evaluated. As shown in Figure S2, the removal efficiency decreased to 67.34% after the first reuse cycle and further declined to only 26% after the second reuse cycle. Therefore, although alkaline methanol effectively eluted phospholipids, the regenerated adsorbent showed a marked loss of phospholipid removal performance in subsequent adsorption cycles. These results indicate that, under the current workflow, B‐Zr/Ti_10%_ MOF is more suitable as a single‐use dispersive clean‐up adsorbent. This use mode can also minimize residual carryover and cross‐contamination between biological samples. The reproducibility of material synthesis is crucial as it ensures the repeatability and reliability of experimental results. Five batches of B‐Zr/Ti_10%_ MOF were prepared using the same synthesis method, and the batch‐to‐batch reproducibility was evaluated by comparing their removal capacities. As shown in Figure S3, no significant differences were observed among the five batches, confirming the high reproducibility and consistency of the adsorbent. To evaluate the removal stability across different matrices, B‐Zr/Ti_10%_ MOF was further applied to three batches of human serum samples. As shown in Figure S4, the adsorbent maintained a high phospholipid clearance rate (96%–99%) in human serum, with an inter‐batch relative standard deviation (RSD) of 1.48%. Figure S5 shows the variation in removal efficiency after storage for 1, 5, 10, 15, and 30 days. The results indicate minimal changes in removal performance throughout the storage period, demonstrating the storage stability of B‐Zr/Ti_10%_ MOF. Subsequently, its stability under different pH conditions (0.1 mol/L HCl, pH 1; water, pH 7; 0.1 mol/L NaOH, pH 13) was evaluated. According to the results in Figure S6, B‐Zr/Ti_10%_ MOF exhibits excellent stability in aqueous solution. The removal capacity of B‐Zr/Ti_10%_ MOF remained unchanged and even slightly improved under acidic conditions. However, a significant decrease in removal capacity was observed under alkaline conditions, indicating that the material possesses excellent acid resistance and moderate alkali resistance. Highly selective materials can efficiently recognize and remove specific targets from complex mixtures, thereby improving the accuracy of analytical results. To evaluate the selectivity of B‐Zr/Ti_10%_ MOF toward phospholipids, 35 organophosphate esters containing phosphate groups were selected. The recoveries of these 35 organophosphate esters ranged from 70% to 120% (Table S3), indicating the strong selectivity of B‐Zr/Ti_10%_ MOF for phospholipids.

Several commercial phospholipid removal products are currently available on the market. B‐Zr/Ti_10%_ MOF was compared with five representative commercial materials under identical sample pretreatment conditions. As shown in Figure 5k, commercial products 1 and 2 exhibited removal efficiencies of approximately 95%, comparable to that achieved by B‐Zr/Ti_10%_ MOF. In contrast, products 4, 5, and 6 showed significantly lower performance, with product 4 achieving a removal rate of 76.6%, and products 5 and 6 showing efficiencies below 20%. These results clearly demonstrate that B‐Zr/Ti_10%_ MOF delivers competitive phospholipid removal performance, fully meeting the practical requirements for biological sample pretreatment.

### 3.3 Adsorption Isotherms and Kinetics

Adsorption kinetics were analyzed by fitting experimental data to theoretical models to elucidate the underlying adsorption mechanism and identify the rate‐limiting steps governing the process. Adsorption isotherms not only describe the equilibrium interaction between the adsorbent and target molecules but also provide essential physicochemical parameters, including adsorption affinity, adsorption state, and maximum adsorption capacity.

**FIGURE 6:**
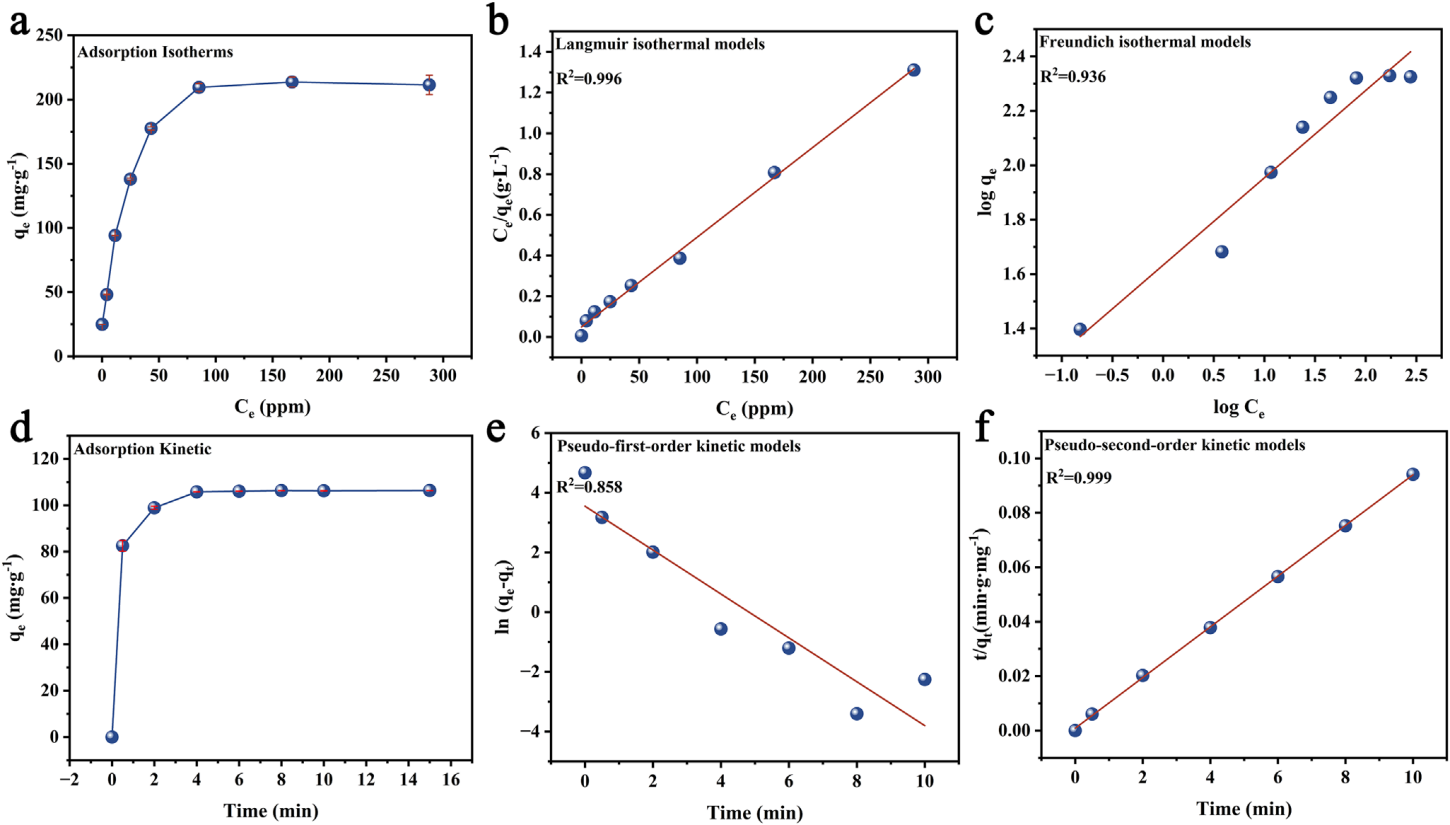
Adsorption Isotherms and kinetics of phospholipids on B‐Zr/Ti_10%_ MOF. (a) The effect of the initial concentration on the adsorption amount of B‐Zr/Ti_10%_ MOF on PC (*n* = 3), (b) the fitting curves of Langmuir and (c) Freundlich isothermal models, (d) the effect of contact time on the adsorption capacity of B‐Zr/Ti_10%_ MOF on PC (*n* = 3), and (e) the fitting curves of the pseudo‐first‐order and (f) the pseudo‐second‐order kinetic models. *Note*: Data are presented as mean ± SD. Red solid lines represent model fitting.

The phospholipid standard PC 16:0/18:1 was employed as a model compound to investigate the adsorption isotherm and kinetic behavior of B‐Zr/Ti_10%_ MOF toward phospholipids. Time‐dependent adsorption experiments were conducted to obtain kinetic profiles, and the data were fitted using pseudo‐first‐order and pseudo‐second‐order kinetic models to evaluate the influence of contact time on adsorption performance (Figure 6d). The adsorption equilibrium of PC 16:0/18:1 on B‐Zr/Ti_10%_ MOF was achieved within 4 min, with the majority of adsorption occurring within the first minute. The adsorption capacity increased gradually between 1 and 4 min before reaching equilibrium. These results indicate a rapid initial adsorption phase driven by abundant accessible active sites, followed by a progressively slower stage as the system approached dynamic equilibrium. The fitting results of the pseudo‐first‐order and pseudo‐second‐order kinetic models are presented in Figure 6e,f and Table 2. Compared with the pseudo‐first‐order model (R^2^ = 0.858), the pseudo‐second‐order model exhibited a significantly higher correlation coefficient (R^2^ = 0.999), indicating a superior fit to the experimental data. Moreover, the adsorption capacity calculated from the pseudo‐second‐order model (107.41 mg g^−^^1^) was in close agreement with the experimentally determined value (106.333 mg g^−^^1^). Collectively, these findings demonstrate that the adsorption of phospholipids onto B‐Zr/Ti_10%_ MOF follows pseudo‐second‐order kinetics, suggesting that the process is predominantly governed by chemisorption involving valence forces or electron sharing between the adsorbent and the target molecules.

**TABLE 2:** Adsorption Kinetic Parameters of PC16:0/18:1 on B‐Zr/Ti_10%_ MOF Fitted by the Pseudo‐first‐order and Pseudo‐second‐order Kinetic Models.

Analyte	q_e, exe_ (mg g^−1^)	pseudo‐first‐orderkineticmodel			pseudo‐second‐orderkineticmodel		
		q_e, cal_ (mg g^−1^)	k_1_ (min^−1^)	R^2^	q_e, cal_ (mg g^−1^)	k_2_ (g mg^−1^ min^−1^)	R^2^
PC16:0/18:1	106.333	34.73	0.7348	0.858	107.41	0.0101	0.999

In addition, the adsorption capacity of B‐Zr/Ti_10%_ MOF was evaluated using phospholipid solutions with varying initial concentrations in the optimized solvent system. Two widely applied isotherm models, the Langmuir and Freundlich models, were employed to elucidate the adsorption behavior of B‐Zr/Ti_10%_ MOF toward PC (Figure 6b,c). The corresponding isotherm parameters are summarized in Table 3. The Langmuir model exhibited a superior linear correlation (R^2^ = 0.996) compared with the Freundlich model (R^2^ = 0.936), indicating that the adsorption of phospholipids on B‐Zr/Ti_10%_ MOF predominantly follows a monolayer adsorption mechanism on a homogeneous surface. This suggests that the active sites are energetically equivalent and that lateral interactions between adsorbed molecules are negligible. The theoretical maximum adsorption capacity (q_max_) calculated from the Langmuir model was 226.76 mg g^−^^1^, which is in close agreement with the experimentally determined saturation capacity of 213.44 mg g^−^^1^. The strong consistency between theoretical and experimental values further validates the applicability of the Langmuir isotherm and supports the conclusion that monolayer adsorption dominates the interaction between B‐Zr/Ti_10%_ MOF and phospholipids.

**TABLE 3:** Adsorption Isotherm Parameters of PC16:0/18:1 on B‐Zr/Ti_10%_ MOF Fitted by the Langmuir and Freundlich Isotherm Models.

Analyte	q_e, exe_ (mg g^−1^)	Langmuir isotherm			Freundlich isotherm		
		q_m_ (mg g^−1^)	k_L_ (L mg^−1^)	R^2^	1/n	K_F_ (mg L^−1^)	R^2^
PC16:0/18:1	213.44	226.76	0.0905	0.996	0.32	42.95	0.936

^∗^ q_e, exp_: adsorption capacity obtained by experiment at equilibrium.

^∗∗^ q_e, cal_: adsorption capacity obtained by the kinetic model.

### 3.4 Adsorption Mechanism of B‐Zr/Ti_10%_ MOF for Phospholipids

**FIGURE 7:**
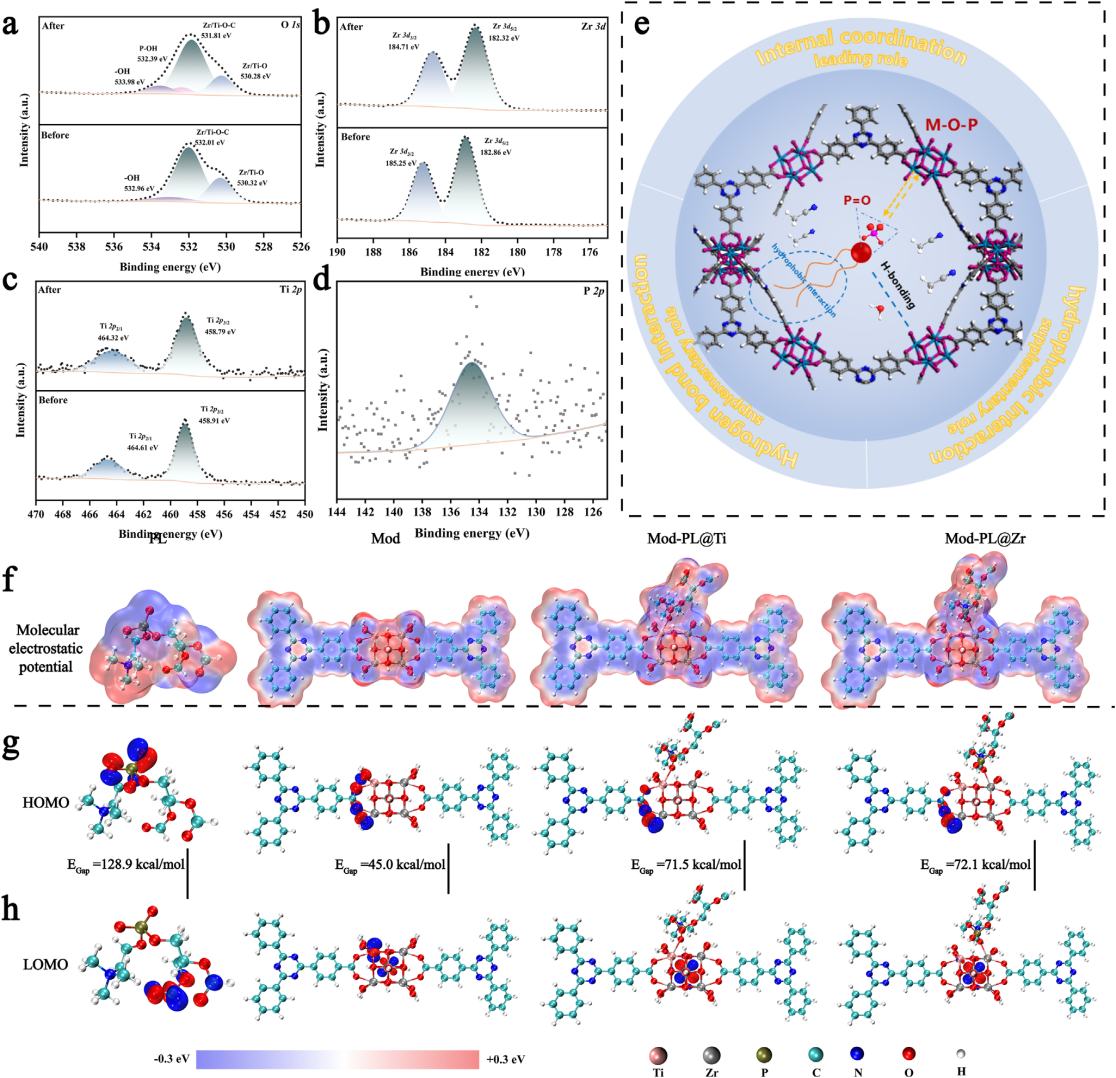
The (a) O1s, (b) Zr 3d, (c) Ti 2p, (d) P 2p XPS spectra of B‐Zr/Ti_10%_ MOF before and after adsorption. (e) Schematic diagram of the adsorption mechanism of B‐Zr/Ti MOF for the adsorption of phospholipid. (f) Molecular electrostatic potential, (g) HOMO, and (h) LUMO of phospholipid, B‐Zr/Ti MOF, B‐Zr/Ti MOF‐PL@Ti, and B‐Zr/Ti MOF‐PL@Zr.

To investigate the mechanism of phospholipid adsorption on B‐Zr/Ti_10%_ MOF, a series of characterization tests were conducted on the adsorbent before and after phospholipid adsorption. FT‐IR spectra of B‐Zr/Ti_10%_ MOF were collected before and after phospholipid adsorption. In the unused material (Figure S7), consistent with our earlier analysis, the broad band at 3000–3700 cm^−^^1^ arises from ─OH stretching. After adsorption, this ─OH envelope diminishes sharply, indicating consumption of node M─OH sites during the process [57]. In addition, two new bands appear at 2934 and 2873 cm^−^^1^, assigned to the asymmetric and symmetric stretching of ─CH_2_ groups from the fatty acyl chains of phospholipids, evidencing hydrophobic/confinement‐assisted uptake of intact hydrocarbon tails. A pronounced intensity increase is also observed in the 1350–1000 cm^−^^1^ fingerprint region, with new peaks at 1253 and 1060 cm^−^^1^ attributable to the asymmetric stretching of P═O and P─O─C, respectively [35]. Moreover, a slight enhancement in the low‐wavenumber region (600–700 cm^−^^1^) is discerned, which can be ascribed to bending vibrations of Zr─O, Ti─O─Zr, and M─O─P linkages. As shown in Figure 7, the appearance of a P 2p doublet after adsorption confirms successful capture of phospholipids. The P 2p binding energy is centered at 134.5 eV (Figure 7f), which is slightly higher than the standard P 2p value of KH_2_PO_4_ (134.0 eV), attributable to decreased electron density at phosphorus during adsorption [58]. In the O 1s spectrum (Figure 7a), the peaks associated with oxygen‐containing groups shifted to 531.81 eV and 530.28 eV, and the P‐OH peak was found after phospholipid adsorption, with a relative increase in the M─O peak area. This aligns with the conversion of surface M─OH to M─O─P through inner coordination, indicating that oxygen‐containing groups participate in the adsorption process of phospholipids onto B‐Zr/Ti_10%_ MOF. As shown in Figure 7d,e, in the Zr 3d spectrum and Ti 2p spectrum, the two fitted peaks shifted slightly toward lower binding energies after adsorption. This indicates electron transfer from the coordinating oxygen to the metal, forming Zr/Ti‐O‐P inner‐sphere complexes on the surface. The local electron density of Zr and Ti slightly increased [59, 60].

To further elucidate the interactions between phospholipids and the adsorbent, DFT calculations were performed using model compounds of both species. Electrostatic potential (ESP) analysis was conducted to identify favorable reactive sites within the adsorption system, where blue regions represent local minima of ESP and red regions denote local maxima. As shown in Figure 7f, the head group region of phospholipids, particularly oxygen‐containing functional groups such as phosphate and carbonyl moieties, exhibits pronounced negative electrostatic potential, indicative of electron‐rich Lewis basic sites. Correspondingly, the Ti/Zr clusters and their adjacent areas display stronger positive electrostatic potential, suggesting their role as the strongest hydrogen bond donors (Lewis acids). These metal‐oxo clusters thus function as Lewis acid centers, inherently enabling electrostatic attraction and site‐specific recognition toward oxygen‐containing coordinating groups [61]. Frontier molecular orbital analysis reveals the electronic‐structure basis for interfacial coupling. As shown in Figure 7g,h, the occupied orbitals of the phospholipid are mainly localized at oxygen‐containing sites in the headgroup, indicating an ability to donate electron density. In contrast, the Ti/Zr node clusters provide acceptor states that more readily receive electron density, resulting in favorable spatial donor–acceptor complementarity between the two components. Upon binding, the HOMO and LUMO, which were previously mainly localized on the framework and node, become more prominently coupled at the node–phospholipid interface, indicating more evident interfacial orbital hybridization. These results suggest that protons associated with the Ti/Zr clusters can adsorb phospholipids through Lewis acid–base interactions. The adsorption not only alters the local structure but also induces electronic coupling and charge redistribution between the node and the headgroup, rather than a purely physical adsorption process [62, 63].

**FIGURE 8:**
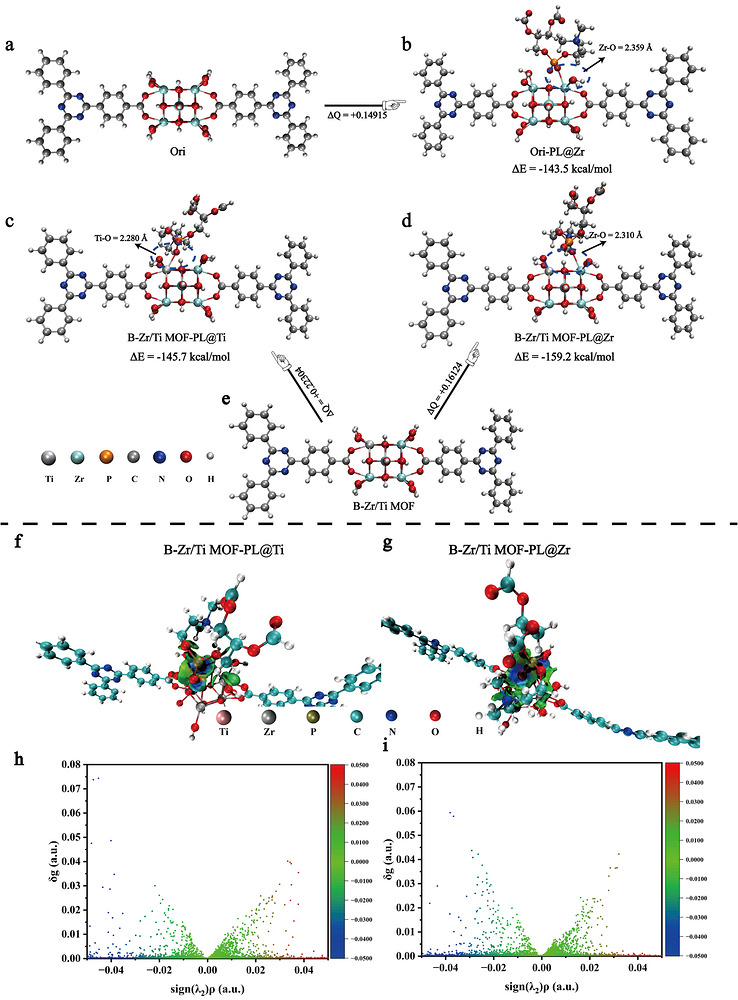
(a, b) Adsorption energy of Ori‐PL@Zr. (c–e) B‐Zr/Ti MOF‐PL@Ti and B‐Zr/Ti MOF‐PL@Zr. (f) IGMH isosurface plots of the host–guest interactions of B‐Zr/Ti MOF‐PL@Ti, (g) B‐Zr/Ti MOF‐PL@Zr, and (h,i) their corresponding scatter plots. In the IGMH analysis, the colors are mapped according to sign(λ2)ρ. Blue regions indicate attractive interactions, including metal–oxygen coordination, electrostatic attraction, or hydrogen‐bond‐like interactions; green regions correspond to weak van der Waals interactions; and red regions represent steric repulsion caused by close contacts [64].

The adsorption energy (E_ads_) was a key metric for describing the adsorption capacity of an adsorbent. A negative E_ads_ value indicated that the adsorption configuration was stable and that the adsorption process was thermodynamically favorable. Based on the optimized complex structures, the E_ads_ values of phospholipids on the B‐Zr/Ti MOF node clusters were further calculated. As shown in Figure 8, the E_ads_ of phospholipids on the PCN‐777 node cluster is −143.5 kcal/mol. In the B‐Zr/Ti MOF node cluster, the E_ads_ at the Ti site is −145.7 kcal/mol, and the E_ads_ at the Zr site is −159.2 kcal/mol, indicating that phospholipid adsorption on the B‐Zr/Ti MOF was likely dominated by chemisorption [65]. Comparison among different configurations further elucidated the binding modes before and after Ti doping. As shown by the comparison of Figure 8b–d, the Ti site exhibited the shortest bond length (2.280 Å) and the largest charge transfer (0.223 e), indicating the formation of the strongest coordination bond with the phospholipid. However, its overall adsorption energy was not the highest. In the Ori‐PL@Zr configuration, the Zr─O contact distance was 2.359 Å, whereas in the doped B‐Zr/Ti MOF‐PL@Zr configuration this distance decreased to 2.310 Å. Although this interaction was weaker than that at the Ti site, Ti doping induced electronic redistribution and improved the overall interaction environment around the Zr site (Figure 8d,g). This change enabled stronger dispersive interactions with the hydrophobic tails of the phospholipid, thereby resulting in a higher E_ads_. Overall, these calculations collectively indicated that Ti doping activated the local coordination environment of adjacent Zr sites through long‐range electronic‐structure modulation. This effect enhanced phospholipid adsorption via a fixation mechanism dominated by node‐internal coordination anchoring and assisted by synergistic weak interactions, leading to more efficient adsorption of phospholipid molecules.

### 3.5 Feasibility of Nontargeted Analysis Application

**FIGURE 9:**
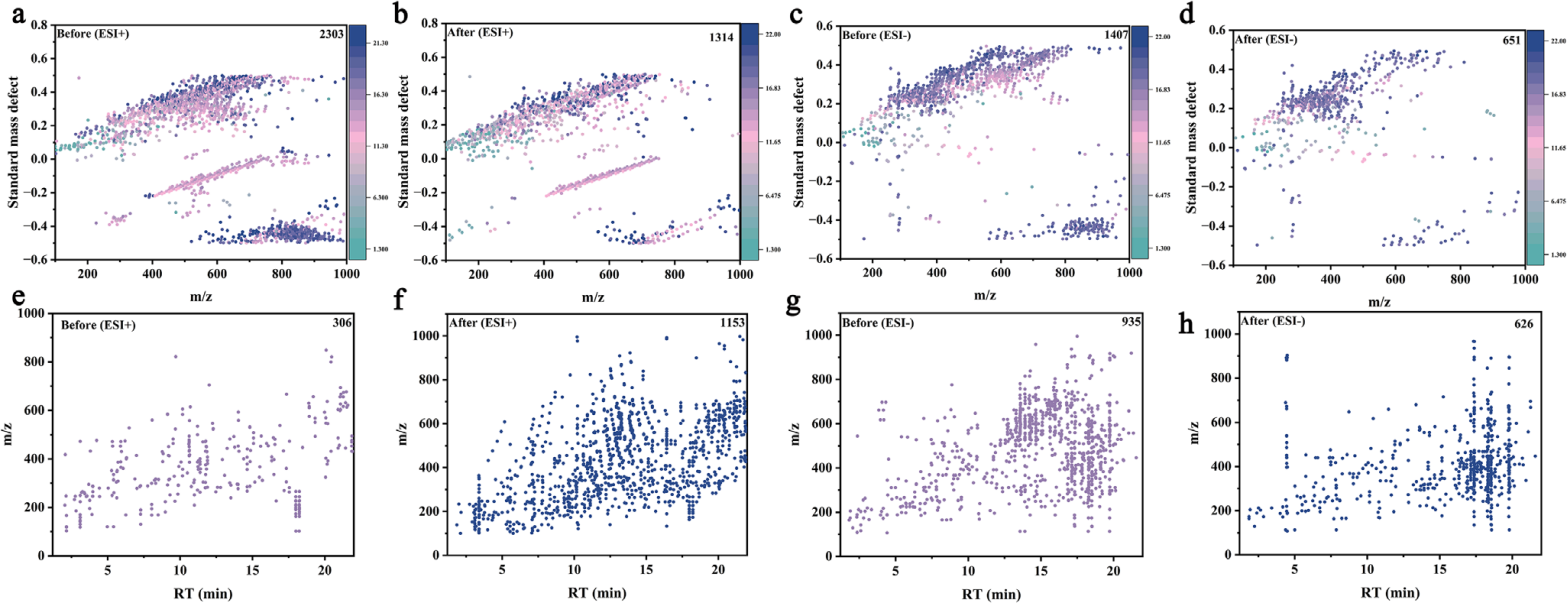
Nontargeted LC‐HRMS feature comparison between After and Before purification. Standard mass defect plots of all nontargeted features in ESI^+^ and ESI^–^ detected in serum extracts by (a,c) before purification and (b,d) after purification, features are colored by RT (1.3–22 min). Quantitative of features (e,g) before and (f,h) after removing the phospholipid characteristic fragment. Numbers in black font are total features by each method in each mode. Feature counts were obtained from three independently prepared serum extracts per group (*n* = 3). Only features detected in all three replicates.

Sample pretreatment is a critical step in nontargeted screening of chemical hazards. Detected exogenous target analytes accounted for only a very small fraction of the small‐molecule components in human serum. Therefore, mass spectrometric acquisition using full‐scan MS1 combined with DIA MS2 enabled a deeper and more comprehensive comparison by considering all nontarget features. The results are shown in the standard mass defect plot and are color‐coded by retention time (RT) (Figure 9). As expected, because phospholipids were intentionally removed using B‐Zr/Ti_10%_ MOF, fewer features were detected in the phospholipid‐depleted group than in the nondepleted group, with reductions of 989 features in ESI^+^ and 756 features in ESI^−^ (Figure 9b,d). The larger decrease in ESI^+^ could be attributed to the fact that the most abundant plasma phospholipids, namely phosphatidylcholines, preferentially ionize in ESI^+^. Feature loss in exposomics mainly occurred in the region of the mass defect plot where phospholipids were expected to appear, indicating that the phospholipid‐removal step exhibited strong selectivity.

Considering all diagnostic phospholipid fragment ions, 86% of all features in the Before group in ESI^+^ (1997 of 2303 features) and 33% in ESI^−^ (472 of 1407 features) were classified as phospholipids (Figure 9e,g). In contrast, in the After group, only 12% of ESI^+^ features (161 of 1314 features) and 3% of ESI^−^ features (25 of 651 features) could be assigned as phospholipids using the same criteria. Meanwhile, the After group showed an increase in detectable nontarget features that could not be classified as phospholipids. More specifically, the After group added 847 non‐phospholipid features in ESI^+^, showing that B‐Zr/Ti_10%_ MOF purification can reduce the interference of phospholipid‐related matrix and expand the coverage of detectable LC‐HRMS features. Overall, B‐Zr/Ti_10%_ MOF effectively removed phospholipids from serum and was successfully applied in nontargeted screening.

### 3.6 Feasibility of Chemical Hazards Analysis Application

**FIGURE 10:**
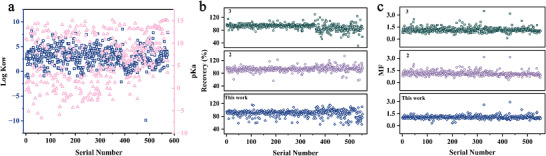
(a) Distribution diagram of dissociation constant (pKa) and n‐octanol–water partition coefficient (log Kow) of chemical hazards. (b) Average recovery (R, %) and (c) matrix effects at 10 µg kg^−1^ for serum of chemical hazards (*n* = 3).

An effective pretreatment strategy should not only be highly selective in reducing complex matrix effects (MEs) but also ensure efficient recovery of diverse target analytes across different sample types [66]. Therefore, ME reduction is a key performance metric in this workflow. As shown in Table S5 and Figure 10a, more than 500 agrochemicals and veterinary drugs with diverse physicochemical properties, including molecular weight, chemical structure, polarity, acidity and basicity, with log Kow ranging from −9.88 to 8.53 and pKa ranging from −6.61 to 15.52, were selected as model chemical hazards to evaluate the feasibility of applying B‐Zr/Ti_10%_ MOF to nontargeted analysis in serum. The MS parameters of these chemical hazards are listed in Table S6. Figure 10b,c showed the recoveries and matrix effects of chemical hazards in serum after phospholipid removal using B‐Zr/Ti_10%_ MOF and two commercial products. For serum samples purified with B‐Zr/Ti_10%_ MOF, 552 compounds achieved acceptable recoveries (r = 50%–120%), with RSDs of 0.09%–20% at 10 µg kg^−1^ All 552 compounds (100%) showed recoveries of ≥50%, meeting the qualitative requirements for nontargeted screening. In addition, 524 of the 552 chemicals (94.92%) showed ME values within 0.7–1.3, indicating only minor matrix suppression or enhancement. Meanwhile, 22 compounds (3.98%) exhibited matrix enhancement and 6 compounds (1.08%) exhibited matrix suppression. For serum treated with commercial product 2, the recoveries of the 552 chemical hazards ranged from 50% to 133%. Among the 552 chemicals, 478 (86.59%) showed ME values within 0.7–1.3, indicating only minor matrix suppression or enhancement. Meanwhile, 57 compounds (10.32%) exhibited matrix enhancement and 17 compounds (3.07%) exhibited matrix suppression. For serum treated with commercial product 3, the recoveries of the 552 chemical hazards ranged from 30% to 130%. Among the 552 chemicals, 466 (84.42%) showed ME values within 0.7–1.3, indicating only minor matrix suppression or enhancement. Meanwhile, 73 compounds (13.22%) exhibited matrix enhancement and 13 compounds (2.35%) exhibited matrix suppression. Compared with the commercial materials, B‐Zr/Ti_10%_ MOF produced weaker matrix effects while maintaining good recoveries of chemical hazards, thereby effectively reducing phospholipid‐induced matrix interference. These results indicated that B‐Zr/Ti_10%_ MOF could substantially mitigate matrix effects in phospholipid‐rich serum and shows considerable potential for nontargeted analysis of chemical hazards with diverse physicochemical properties in phospholipid‐rich biological matrices.

## 4 Conclusions

In summary, this study successfully synthesized B‐Zr/Ti MOF via a hydrothermal method, employing Zr as the metal node and H_3_TATB as the organic linker. We present a novel strategy for the removal of phospholipids from phospholipid‐rich samples. The incorporation of Ti as a secondary metal into the Zr‐based MOF framework introduced diverse adsorption sites and interaction mechanisms, which significantly enhanced both the removal capacity and selectivity for phospholipids. Experimental results confirmed the excellent binding affinity of the synthesized material towards phospholipids. The prepared B‐Zr/Ti MOF possesses suitable mesoporous characteristics and active sites for phospholipid removal, while its pore channels facilitate enhanced mass transfer rates. The material demonstrated efficient recovery for over 500 chemical hazards and outstanding phospholipid removal capability, indicating its considerable potential for the nontargeted analysis of phospholipid‐rich biological samples. These findings not only establish a new avenue for phospholipid removal but also highlight the transformative potential of Zr/Ti bimetallic MOFs, offering a promising solution to the challenges associated with phospholipid removal in serum during human biomonitoring.

## Author Contributions

**Yan Qi**: conceptualization, methodology, writing – review and editing, software, formal analysis, data curation. **Feier Bai**: investigation, validation, software, data curation. **Yanmin Liang**: conceptualization, investigation, writing – original draft, methodology, validation, visualization, writing – review and editing, software, formal analysis, data curation. **Jing Zhang**: investigation, validation, software, project administration, supervision, resources. **Hongyue Ma**: investigation, methodology, software, data curation. **Bing Shao**: conceptualization, investigation, funding acquisition, writing – review and editing, project administration, data curation, supervision, resources.

## Conflicts of Interest

The authors declare no conflicts of interest.

## Supporting information




**Supporting File**: advs76529‐sup‐0001‐SuppMat.docx.

## Data Availability

The data that support the findings of this study are available from the corresponding author upon reasonable request.
