# Supplementary material for: Ti‐Doping Activates Adjacent Zr Sites in Bimetallic MOFs for Cooperative Phospholipid Removals in Human Biomonitoring
Source: Adv Sci (Weinh). 2026 Jul 13;13(55):e76529. doi: 10.1002/advs.76529 (PMC13360115; doi:10.1002/advs.76529)
Supplement: Supplementary file 1 [file ADVS-13-e76529-s001.docx]

**Ti-Doping Activates Adjacent Zr Sites in Bimetallic MOFs for Cooperative Phospholipid removals in Human Biomonitoring**

1. **Supplementary texts**

**1.1 UPLC-MS/MS conditions for analysis of phospholipids**

For phospholipids, Waters Acquity Ultra Performance LC system coupled with a Waters Xevo TQ-XS mass was used for UPLC-MS/MS analysis. The chromatographic separation was carried out on an ACQUITY UPLC CSH C18 column (2.1×100 mm, 1.8 μm), the column temperature was maintained at 40°C, and the flow rate was 0.3 mL∙min-1. The injection volume was 2.0 μL. The mobile phases were (A) acetonitrile-water (6: 4, v/v) containing 0.1% formic acid and 10 mM ammonium formate, and (B) isopropanol-acetonitrile (9: 1, v/v) containing 0.1% (v/v) formic acid and 10 mM ammonium formate. The gradient elution procedure was 40%-43% B (0-2.0 min), 43%-50% B (2.0-2.1min), 50%-54% B (2.1-12.0min) and 54%-70% B (12.0-12.1 min). The mass spectrometer was operated by Parent (Precursor) Ion Scan (PSI), Multi-reaction Monitoring (MRM) mode and positive electrospray ionization (ESI) source. The optimized main parameters were set as follows: capillary voltage 3.0 kV; Desolvent gas 1000 L∙h^-1^; Cone hole gas 150 L∙h^-1^; Ion source temperature 150°C; Desolvent gas temperature 450°C; Ion energy 0.5.

**1.2 UPLC-MS/MS conditions for analysis of chemical hazards**

For pesticides, Waters Acquity ultra-performance liquid chromatography system combined with Waters Xevo TQ-S mass spectrometer was used for UPLC-MS/MS analysis. ACQUITY ultra-high performance liquid chromatography HSS T3 column (2.1×100 mm, 1.8 μm) was used for chromatographic separation. The column temperature was maintained at 40°C and the flow rate was 0.3 mL min. The injection volume was 2.0 μL. The mobile phase was (A): ultrapure water with 0.01% (*v*/*v*) formic acid and 2 mM ammonium acetate; (B): methanol with 0.01% (*v*/*v*) formic acid and 2 mM ammonium acetate. The liquidity gradient procedures were as follows: 0-1.0 min(3% B, 97% A), 1.0-1.5 min (3%-15% B, 97%-85% A), 1.5-2.5 min (15%-50% B, 85%-50% A), 2.5-18.0 min (50%-70% B, 50%-30% A), 18.0–23.0 min (70%-98% B, 30%-2% A), 23.0–27.0 min (98% B, 2% A), 27.0–27.1 min (98%-3% B, 2%-97% A), 27.1–30.0 min (3% B, 97% A). The mass spectrometer was operated in multi-reaction monitoring (MRM) mode and positive electrospray ionization (ESI) source. The optimized main parameters were set as follows: capillary voltage was 3.0 kV; Desolvent gas was 1000 L∙h^-1^; Cone hole gas was 150 L∙h^-1^; Ion source temperature was 150°C; Desolvent gas temperature was 450°C; Ion energy was 0.5.

For veterinary drugs, UPLC-MS/MS analysis was carried out on Waters Acquity ultra-high performance liquid chromatography system, which is equipped with Waters Xevo TQ-XS mass spectrometer. Chromatographic separation was performed using an ACQUITY UPLC BEH C18 column (2.1 × 100 mm, 1.7 μm) maintained at 40℃ at a flow rate of 0.3 mL/min. The injection volume μm 3.0 μL. The mobile phase was A:0.1% (*v*/*v*) formic acid and 0.5 mM ammonium fluoride ultrapure water, and (b) methanol/acetonitrile (*v*/*v* = 1/1). The gradient of mobile phase is as follows: 0-2.0 min (3% B, 97% A), 2.0-5.0 min (3%-15% B, 97%-85% A), 5.0-10.0 min (15% B, 85% A), 10.0-15.0 min (15%-30% B, 85%-70% A), 5.0–20.0 min (30 %-50 % B, 70%-50% A), 20.0–24.0 min (50 %-100 % B,50%-0% A), 24.0–28.0 min (100 %B,0% A), 28.0–28.5 min (100 %-3% B, 0%-97% A), 28.5–29.0 min (3 % B, 97 % A). The main parameters were optimized as follows: capillary voltage was 3.0kV; Desolvent gas was 800 L∙h^-1^; Conical gas was 150 liters L∙h^-1^; Source temperature was 150°C; Solvent removal gas temperature was 400℃; Ion energy was 0.5.

**1.3 LC-HRMS Analysis conditions for NonTargeted analysis**

The HRMS was operated with parallel full scan (i.e., MS1; 100–1000 mass-to-charge ratio (m/z), 120,000 nominal resolution) and data-independent acquisition (DIA) MS/MS (i.e., MS2; 30,000 nominal resolution) with four m/z precursor windows of equal size (237 Da), with 10 Da window overlap. Injection volumes were 5 μL . Chromatography was at 40 °C on an Acquity BEH C18 column (130 Å, 1.7 μm, 3 × 100 mm, Waters). A binary gradient elution at 0.4 mL/min used mobile phases: ESI+ (A) water containing 0.1% formic acid, (B) methanol containing 0.1% formic acid; ESI− (A) water containing 2mM ammonium acetate, (B) methanol containing 2mM ammonium acetate. The elution gradient started at 2% B, linearly increased to 99% B by 20 min, held until 25 min, and returned to initial conditions with 5 min equilibration.

**1.4 LC-HRMS data processing and analysis**

Downstream data analysis was performed following previously reported methods with slight modifications[1]. Raw data were preprocessed in MS-DIAL (version 4.80) through inter-sample feature alignment, deconvolution of MS1 and DIA MS2 spectra, and peak integration. The databases used were ESI(+)-MS/MS from authentic standards (16,232 unique compounds) and ESI(–)-MS/MS from authentic standards (8,887 unique compounds). The MS-DIAL–processed data were exported and further analyzed in Python. Only molecular features that were present in all three replicates of the pooled plasma, eluted after the analysis void volume (retention time RT > 1.3 min), and exhibited signal intensities at least five times higher than the corresponding procedural blank were included in the analysis. To compare feature overlap between “Before” and “After” groups, features were matched using a 0.002 Da m/z tolerance and a 0.9 min RT tolerance. Phospholipid-related features were classified based on characteristic diagnostic fragment ions observed in the DIA-MS/MS spectra, with a mass error tolerance of 5 ppm. The diagnostic ions used for phospholipid feature annotation are detailed in Table S4[2-4].

2. **Supplementary figures**

Figure S1. SEM images morphological of B-Zr/Ti MOF with varying Ti contents (0–50%)(a–e).

Figure S2. Recycling performance of B-Zr/Ti_10%_ MOF after elution (n = 3). Note: Data are presented as mean ± SD.

Figure S3. Removal efficiency by different synthetic batches of B-Zr/Ti_10%_ MOF (n = 3). Note: Data are presented as mean ± SD.

Figure S4. Removal efficiency of phospholipids by B-Zr/Ti_10%_ MOF in different batches of human serum (n = 3). Note: Data are presented as mean ± SD.

Figure S5. Removal efficiency of B-Zr/Ti_10%_ MOF after storage for different days (n = 3). Note: Data are presented as mean ± SD.

Figure S6. Removal efficiency of B-Zr/Ti_10%_ after different pH treatments (n = 3). Note: Data are presented as mean ± SD.

Figure S7. The FT-IR spectra of B-Zr/Ti_10%_ MOF before and after adsorption.

3. **Supplementary tables**

Table S1. Information about different commercial products.

| Product | Product form | filler |
| --- | --- | --- |
| This work | Dispersed adsorbent | B-Zr/Ti_10%_ MOF |
| 2 | Solid phase extraction 96-well plate | Polypropylene |
| 3 | Solid phase extraction 96-well plate | -- |
| 4 | solid phase extraction column | silicon zirconium skeleton composite porous material |
| 5 | Solid phase extraction 96-well plate | inorganic polymer |
| 6 | Supported Liquid Extraction 96-well plate | Specially treated diatomite |

Table S2. Mass spectrometric parameters of 20 phospholipids.

| Serial Number | Phospholipid | Retention time [min] | Ions pairs [m/z] | | Collision Energy [eV] | | Cone voltage [V] |
| --- | --- | --- | --- | --- | --- | --- | --- |
|  |  |  | Quantitative | Qualification | Quantitative | Qualification |  |
| 1 | LPE 16:0 | 2.14 | 454.30313.20 | 454.30/313.20 | 25 | 25 | 30 |
| 2 | LPE 18:0 | 3.11 | 482.30/341.23 | 482.30/82.85 | 20 | 15 | 30 |
| 3 | LPC 15:0 | 1.69 | 482.30/184.10 | 482.30/184.10 | 30 | 30 | 10 |
| 4 | LPC 16:0 | 2.01 | 496.30/183.99 | 496.30/103.93 | 25 | 25 | 30 |
| 5 | LPC 18:0 | 2.94 | 524.46/184.06 | 524.40/103.98 | 25 | 30 | 30 |
| 6 | LPC18:1 | 2.11 | 522.38/183.95 | 572.30124.95 | 20 | 35 | 25 |
| 7 | PC 14:0/16:0 | 10.43 | 706.46/183.95 | 706.46/183.95 | 25 | 25 | 40 |
| 8 | PC p-16:0/16:0 | 13.63 | 718.43/184.20 | 718.43/99.93 | 30 | 50 | 35 |
| 9 | PC 16:0/16:0 | 13.29 | 734.50/183.95 | 734.50/100.00 | 25 | 25 | 30 |
| 10 | PC 16:0/18:1 | 13.35 | 762.40/184.23 | 762.40/100.00 | 25 | 30 | 35 |
| 11 | PC 16:0/18:0 | 13.35 | 762.51/183.95 | 762.51/100.00 | 30 | 20 | 30 |
| 12 | PC p-18:0/18:0 | 13.69 | 774.60/184.10 | 774.60/184.10 | 30 | 30 | 20 |
| 13 | PC 18:2/18:2 | 10.88 | 783.22/184.02 | 783.22/184.02 | 20 | 20 | 35 |
| 14 | PC 18:1/18:1 | 13.39 | 786.51/183.99 | 786.51/124.47 | 25 | 50 | 30 |
| 15 | PC 18:0/18:2 | 13.38 | 787.31/184.16 | 787.31/100.14 | 25 | 20 | 30 |
| 16 | SM d18:1/14:1 | 5.82 | 673.50/184.10 | 673.50/184.10 | 30 | 30 | 50 |
| 17 | SM d18:1/14:0 | 7.68 | 675.50/184.10 | 675.50/184.10 | 30 | 30 | 50 |
| 18 | SM d18:1/16:0 | 10.53 | 703.54/184.00 | 703.54/184.00 | 25 | 25 | 35 |
| 19 | SM d18:1/18:1 | 10.94 | 729.54/183.95 | 729.54/100.14 | 35 | 15 | 50 |
| 20 | SM d18:1/18:0 | 10.34 | 731.30/183.99 | 731.30/183.99 | 25 | 25 | 30 |

Table S3. Structural information, recovery rate and matrix effect of 35 organicphosphate esters (n = 3).

| Serial Number | Compounds | IUPAC | Formula | R (%) | MF | CAS No | Structure |
| --- | --- | --- | --- | --- | --- | --- | --- |
| 1 | DMP | Dimethyl phosphate | C_2_H_7_O_4_P | 102.01 | 1.03 | 813-78-5 |  |
| 2 | TMP | Trimethyl phosphate | C_3_H_9_O_4_P | 95.68 | 1.21 | 512-56-1 |  |
| 3 | DEP | Diethyl phosphate | C_4_H_11_O_4_P | 102.22 | 1.06 | 598-02-7 |  |
| 4 | TEP | Triethyl phosphate | C_6_H_15_O_4_P | 101.75 | 1.05 | 78-40-0 |  |
| 5 | TCEP | Tris(2-chloroethyl) phosphate | C_6_H_12_Cl_3_O_4_P | 102.31 | 0.96 | 115-96-8 |  |
| 6 | TEEDPP | Tetraethyl ethylene diphosphonate | C_10_H_24_O_6_P_2_ | 96.27 | 1.09 | 995-32-4 |  |
| 7 | TMCPP | Tris(3-chloropropyl) phosphate | C_9_H_18_Cl_3_O_4_P | 113.26 | 0.85 | 1067-98-7 |  |
| 8 | TCEPI | Tris(2-chloroethyl) phosphate | C_9_H_18_Cl_3_O_4_P | 91.57 | 1.12 | 6145-73-9 |  |
| 9 | MDPP | Methyl diphenyl phosphate | C_13_H_13_O_4_P | 115.65 | 0.89 | 115-89-9 |  |
| 10 | DPPA | Diphenyl azidophosphate | C_12_H_10_N_3_O_3_P | 103.08 | 0.96 | 26386-88-9 |  |
| 11 | TAP | Triallyl phosphate | C9H15O4P | 109.01 | 0.99 | 1623-19-4 |  |
| 12 | V6 | 2,2-Bis(chloromethyl) trimethylene bis[bis(2-chloroethyl)phosphate] | C_13_H_24_Cl_6_O_8_P_2_ | 108.28 | 0.95 | 38051-10-4 |  |
| 13 | 4-OH-TPHP | 4-hydroxyphenyl diphenyl phosphate | C_18_H_15_O_5_P | 107.20 | 0.93 | 56806-74-7 |  |
| 14 | TCIPP | Tris(1-chloro-2-propyl) phosphate | C_9_H_18_Cl_3_O_4_P | 108.43 | 0.98 | 13674-84-5 |  |
| 15 | TDCIPP | Tis(1,3-dichloro-2-propyl) phosphate | C_9_H_15_Cl_6_O_4_P | 118.72 | 0.85 | 13674-87-8 |  |
| 16 | TBOEP | Tris(2-butoxyethyl) phosphate | C_18_H_39_O_7_P | 103.04 | 1.03 | 78-51-3 |  |
| 17 | TPPO | Triphenyl phosphine oxide | C_18_H_15_OP | 102.81 | 1.03 | 791-28-6 |  |
| 18 | TNBP | Tributyl phosphate | C_12_H_27_O_4_P | 111.75 | 0.97 | 126-73-8 |  |
| 19 | TPRP | Tripropyl phosphate | C_9_H_21_O_4_P | 109.97 | 0.96 | 513-08-6 |  |
| 20 | TBPO | Tributyl phosphine oxide | C_12_H_27_O_P_ | 74.86 | 1.01 | 814-29-9 |  |
| 21 | DOPO | 6H-dibenz(C,E)(1,2)oxaphosphorin-6-oxide | C_12_H_9_O_2_P | 109.02 | 1.04 | 35948-25-5 |  |
| 22 | TCRP | Tricresyl phosphate | C_21_H_21_O_4_P | 117.60 | 0.89 | 1330-78-5 |  |
| 23 | TDBPP | Tris-(2,3-Dibromopropyl)phosphate | C_9_H_15_Br_6_O_4_P | 112.49 | 0.96 | 126-72-7 |  |
| 24 | TPHP | Triphenyl phosphate | C_18_H_15_O_4_P | 110.63 | 0.97 | 115-86-6 |  |
| 25 | TPP | Tripentyl phosphate | C_15_H_33_O_4_P | 112.76 | 0.94 | 2528-38-3 |  |
| 26 | BDPP | Butyl diphenyl phosphate | C_16_H_19_O_4_P | 112.94 | 0.96 | 2752-95-6 |  |
| 27 | IDPP | Isopropyl diphenyl phosphate | C_15_H_17_O_4_P | 114.55 | 0.90 | 60763-39-5 |  |
| 28 | DBPP | Dibutyl phenyl phosphate | C_14_H_23_O_4_P | 112.37 | 0.95 | 2528-36-1 |  |
| 29 | CDP | Cresyl diphenyl phosphate | C_19_H_17_O_4_P | 111.11 | 0.92 | 26444-49-5 |  |
| 30 | B4tBPPP | Tris(4-tert-butylphenyl) phosphate | C_30_H_39_O_4_P | 119.44 | 0.88 | 78-33-1 |  |
| 31 | TXP | Trixylyl phosphate | C_8_H_11_O_4_P | 116.68 | 0.90 | 25155-23-1 |  |
| 32 | BEHPP | Bis(2-ethylhexyl) phenyl phosphate | C_22_H_39_O_4_P | 119.15 | 0.89 | 16368-97-1 |  |
| 33 | IDDPP | Isodecyl diphenyl phosphate | C_22_H_31_O_4_P | 117.54 | 0.86 | 29761-21-5 |  |
| 34 | DOPP | Dioctyl phenyl phosphonate | C_22_H_39_O_3_P | 109.24 | 0.91 | 1754-47-8 |  |
| 35 | TEHP | Tris(2-ethylhexyl) phosphate | C_24_H_51_O_4_P | 119.55 | 0.90 | 78-42-2 |  |

Table S4. Classification of phospholipid features.

| ESI^+^/^-^ | Phospholipid feature | Fragment Ions |
| --- | --- | --- |
| ESI^+^ | Phosphatidylcholines and sphingomyelins | ([C_5_H_15_O_4_NP]^+^, m/z 184.0733) |
|  | Phosphatidylcholines | ([C_5_H_14_NO]^+^, m/z 104.1070) |
|  | Ceramides | ([C_18_H_34_N]^+,^ m/z 264.2686) |
|  | Ceramides | ([C_18_H_36_N]^+^, m/z 266.2842) |
|  | Phosphatidylcholines and sphingomyelins | ([C_2_H_5_NaO_4_P]^+^, m/z 146.9817) |
|  | Phosphatidylcholines and sphingomyelins | ([C_5_H_13_O_3_NP]^+^, m/z 166.0627) |
|  | Phosphatidylethanolamines | ([C_2_H_8_NNaO_4_P]^+^, m/z 164.0083) |
|  | Phosphatidylcholines and sphingomyelins | ([C_2_H_6_O_4_P]^+^, m/z 124.9998) |
| ESI^-^ | All phospholipids | ([C_3_H_6_O_5_P]^-^, m/z 152.9958) |
|  | Phosphatidylcholines and sphingomyelins | ([C_4_H_11_O_4_NP]^-^, m/z 168.0431) |
|  | Phosphatidylinositols | ([C_6_H_10_O_8_P]^-^, m/z 241.0119) |
|  | Phosphatidylethanolamines | ([C_2_H_7_O_4_NP]^-^, m/z 140.0118) |
|  | Phosphatidylethanolamines | ([C_5_H_11_O_5_NP]^-^, m/z 196.0380) |
|  | Phosphatidylcholines | ([C_7_H_17_NO_6_P]^-^, m/z 242.0798) |
|  | Phosphatidylethanolamines | ([H_3_NaO_4_P]^-^, m/z 120.9660) |
|  | Phosphatidylcholines | ([C_7_H^15^NO^5^P]^-^, m/z 224.0693) |

Table S5. Structure and physicochemical properties of different agricultural and veterinary drugs.

| Serial  number | Analytes | Purity (%) | p*K*a^a)^ | log *Kow*^b)^ | CAS No. | Molecular Formula | Molecular Mass | Molecular Structures | Sizes^c)^ |
| --- | --- | --- | --- | --- | --- | --- | --- | --- | --- |
| 1 | Acephate | 99.9 | 11.00±0.46 | -0.90 | 30560-19-1 | C_4_H_10_NO_3_PS | 183.17 |  | 4.5 Å ×5.7 Å×5.9 Å |
| 2 | Acetamiprid | 99.7 | -0.44±0.10 | 2.55 | 135410-20-7 | C_10_H_11_ClN_4_ | 222.67 |  | 8.3 Å ×4.9 Å×5.3 Å |
| 3 | Acetochlor | 99.5 | 1.29±0.50 | 3.03 | 34256-82-1 | C_14_H_20_ClNO_2_ | 269.77 |  | 7.6 Å ×8.0 Å×6.4 Å |
| 4 | Alachlor | 99.4 | 1.20±0.50 | 3.52 | 15972-60-8 | C_14_H_20_ClNO_2_ | 269.77 |  | 7.7 Å ×7.2 Å×6.8 Å |
| 5 | Albendazole | 98.9 | 10.72±0.10 | 3.14 | 54965-21-8 | C_12_H_15_N_3_O_2_S | 265.33 |  | 6.6 Å ×11.3 Å×9.1 Å |
| 6 | Aldicarb | 99.9 | - ^d)^ | 1.36 | 116-06-3 | C_7_H_14_N_2_O_2_S | 190.26 |  | 7.1 Å ×7.6 Å×3.8 Å |
| 7 | Aldicarb sulfone | 99.2 | 13.44±0.46 | -0.57 | 1646-88-4 | C_7_H_14_N_2_O_4_S | 222.26 |  | 6.7 Å ×10.1 Å×4.0 Å |
| 8 | Aldicarb sulfoxide | 98.2 | 13.57±0.46 | -0.78 | 1646-87-3 | C_7_H_14_N_2_O_3_S | 206.26 |  | 9.4 Å ×5.2 Å×4.4 Å |
| 9 | Ametoctradin | 99.9 | - | 4.02 | 865318-97-4 | C_15_H_25_N_5_ | 275.39 |  | 16.1 Å ×7.1 Å×2.1 Å |
| 10 | Amidosulfuron | 99.9 | 0.12±0.40 | -1.29 | 120923-37-7 | C_9_H_15_N_5_O_7_S_2_ | 369.37 |  | 8.5 Å ×10.8 Å×6.2 Å |
| 11 | Amisulbrom | 99.9 | -5.12±0.50 | 2.00 | 348635-87-0 | C_13_H_13_BrFN_5_O_4_S_2_ | 466.31 |  | 6.7 Å ×13.9 Å×6.3 Å |
| 12 | Anilofos | 98.4 | -0.48±0.50 | 3.89 | 64249-01-0 | C_13_H_19_ClNO_3_PS_2_ | 367.85 |  | 7.2 Å ×7.6 Å×10.1 Å |
| 13 | Atrazine | 98.8 | 1.64 | 2.61 | 1912-24-9 | C_8_H_14_ClN_5_ | 215.68 |  | 9.4 Å ×7.0 Å×3.5 Å |
| 14 | Avermectin B1a | 99.2 | - | 3.82 | 71751-41-2 | C_48_H_72_O_14_ | 887.11 |  | 11.7 Å ×16.8 Å×10.2 Å |
| 15 | Azinphos-methyl | 99.6 | - | 2.53 | 86-50-0 | C_10_H_12_N_3_O_3_PS_2_ | 317.32 |  | 12.5 Å ×5.0 Å×7.4 Å |
| 16 | Azoxystrobin | 99.9 | -0.93±0.18 | 2.5 | 131860-33-8 | C_22_H_17_N_3_O_5_ | 403.39 |  | 8.9 Å ×14.0 Å×11.0 Å |
| 17 | Benalaxyl | 99.9 | 1.52±0.50 | 3.69 | 71626-11-4 | C_20_H_23_NO_3_ | 325.4 |  | 11.3 Å ×6.7 Å×6.6 Å |
| 18 | Bendiocarb | 99.3 | - | 2.55 | 22781-23-3 | C_11_H_13_NO_4_ | 223.23 |  | 9.5 Å ×6.4 Å×4.3 Å |
| 19 | Bensulfuron-methyl | 99.6 | 12.34±0.70 | 1.41 | 83055-99-6 | C_16_H_18_N_4_O_7_S | 410.4 |  | 9.6 Å ×10.3 Å×6.5 Å |
| 20 | Benzovindiflupyr | 99.4 | 12.12±0.20 | 4.36 | 1072957-71-1 | C_18_H_15_Cl_2_F_2_N_3_O | 398.23 |  | 11.0 Å ×7.3 Å×5.6 Å |
| 21 | Benzoximate | 99.9 | - | 4.20 | 29104-30-1 | C_18_H_18_ClNO_5_ | 363.79 |  | 8.3 Å ×9.8 Å×5.2 Å |
| 22 | Bifenox | 99.7 | - | 4.48 | 42576-02-3 | C_14_H_9_Cl_2_NO_5_ | 342.13 |  | 8.3 Å ×9.8 Å×5.2 Å |
| 23 | Bifenthrin | 99.9 | - | 8.15 | 83322-02-5 | C_23_H_22_ClF_3_O_2_ | 422.87 |  | 7.5 Å ×14.7 Å×4.6 Å |
| 24 | Bioresmethrin | 98.3 | - | 7.11 | 28434-01-7 | C_22_H_26_O_3_ | 338.44 |  | 14.8 Å ×9.6 Å×6.4 Å |
| 25 | Bitertanol | 99.8 | 13.40±0.20 | 4.16 | 55179-31-2 | C_20_H_23_N_3_O_2_ | 337.42 |  | 12.0 Å ×9.3 Å×7.0 Å |
| 26 | Boscalid | 99.7 | 10.75±0.70 | 4.00 | 188425-85-6 | C_18_H_12_Cl_2_N_2_O | 343.21 |  | 6.8 Å ×11.2 Å×5.6 Å |
| 27 | Bromuconazole | 99.9 | 2.75±0.10 | 3.24 | 116255-48-2 | C_13_H_12_BrCl_2_N_3_O | 377.06 |  | 6.3 Å ×7.7 Å×5.0 Å |
| 28 | Bupirimate | 95.7 | 5 | 2.70 | 41483-43-6 | C_13_H_24_N_4_O_3_S | 316.42 |  | 8.2 Å ×10.8 Å×5.0 Å |
| 29 | Buprofenzin | 99.3 | 3.02±0.20 | 4.30 | 69327-76-0 | C_16_H_23_N_3_OS | 305.44 |  | 7.3 Å ×8.2 Å×8.4 Å |
| 30 | Butachlor | 99.6 | 1.20±0.50 | 4.84 | 23184-66-9 | C_17_H_26_ClNO_2_ | 311.85 |  | 9.6 Å ×9.4 Å×8.0 Å |
| 31 | Butralin | 99.1 | -3.50±0.50 | 2.95 | 33629-47-9 | C_14_H_21_N_3_O_4_ | 295.33 |  | 9.1 Å ×7.7 Å×5.8 Å |
| 32 | Cadusafos | 98.1 | - | 3.90 | 95465-99-9 | C_10_H_23_O_2_PS_2_ | 270.39 |  | 7.2 Å ×6.7 Å×7.8 Å |
| 33 | Carbaryl | 99.5 | 12.02±0.46 | 2.36 | 63-25-2 | C_12_H_11_NO_2_ | 201.22 |  | 8.7 Å ×5.2 Å×6.8 Å |
| 34 | Carbendazim | 99.9 | 4.48 | 1.52 | 10605-21-7 | C_9_H_9_N_3_O_2_ | 191.19 |  | 6.5 Å ×7.9 Å×6.6 Å |
| 35 | Carbofuran | 99.9 | - | 2.32 | 1563-66-2 | C_12_H_15_NO_3_ | 221.25 |  | 9.2 Å ×7.5 Å×5.7 Å |
| 36 | Carbofuran-3-hydroxy | 98.7 | 12.28±0.46 | 0.76 | 16655-82-6 | C_12_H_15_NO_4_ | 237.25 |  | 9.2 Å ×7.5 Å×5.7 Å |
| 37 | Carboxin | 99.7 | 14.31±0.70 | 1.49 | 5234-68-4 | C_12_H_13_NO_2_S | 235.30 |  | 6.7 Å ×7.0 Å×5.4 Å |
| 38 | Carfentrazone-ethyl | 99.5 | -2.26±0.20 | 4.26 | 128639-02-1 | C_15_H_14_Cl_2_F_3_N_3_O_3_ | 412.19 |  | 12.5 Å ×8.2 Å×5.7 Å |
| 39 | Chlorantranilipole | 99.3 | 10.19±0.70 | 3.98 | 500008-45-7 | C_18_H_14_BrCl_2_N_5_O_2_ | 483.15 |  | 8.6 Å ×7.7 Å×7.5 Å |
| 40 | Chlorbenzuron | 99.5 | 9.93±0.23 | 3.15 | 196791-54-5 | C_14_H_10_Cl_2_N_2_O_2_ | 309.15 |  | 10.0 Å ×10.8 Å×2.5 Å |
| 41 | Chlordimeform | 97.0 | 7.89±0.50 | 2.89 | 6164-98-3 | C_10_H_13_ClN_2_ | 196.68 |  | 6.0 Å ×8.6 Å×3.6 Å |
| 42 | Chlorfenvinphos | 99.3 | - | 3.81 | 470-90-6 | C_12_H_14_Cl_3_O_4_P | 359.57 |  | 7.8 Å ×8.6 Å×7.0 Å |
| 43 | Chlorfluazuron | 99.9 | 8.10 | 5.87 | 71422-67-8 | C_20_H_9_Cl_3_F_5_N_3_O_3_ | 540.65 |  | 10.8 Å ×9.5 Å×8.4 Å |
| 44 | Chloridazon | 99.0 | 0.71±0.20 | 1.14 | 1698-60-8 | C_10_H_8_ClN_3_O | 221.64 |  | 8.6 Å ×6.6 Å×4.4 Å |
| 45 | Chlorimuron ethyl | 99.7 | 3.78±0.10 | 2.29 | 90982-32-4 | C_15_H_15_ClN_4_O_6_S | 414.82 |  | 8.9 Å ×7.7 Å×5.2 Å |
| 46 | Chlorpropham | 99.9 | 13.06±0.70 | 3.31 | 101-21-3 | C_10_H_12_ClNO_2_ | 213.66 |  | 6.8 Å ×6.3 Å×5.7 Å |
| 47 | Chlorpyrifos | 99.9 | - | 4.96 | 2921-88-2 | C_9_H_11_Cl_3_NO_3_PS | 350.59 |  | 10.1 Å ×6.1 Å×7.0 Å |
| 48 | Chlorpyriphos-methyl | 99.4 | -5.59±0.10 | 4.13 | 5598-13-0 | C_7_H_7_Cl_3_NO_3_PS | 322.53 |  | 6.0 Å ×9.9 Å×3.5 Å |
| 49 | Chlorsulfuron | 99.9 | - | 2.14 | 64902-72-3 | C_12_H_12_ClN_5_O_4_S | 357.77 |  | 13.3 Å ×11.3 Å×3.8 Å |
| 50 | Chlortoluron | 99.7 | 14.43±0.70 | 2.41 | 15545-48-9 | C_10_H_13_ClN_2_O | 212.68 |  | 8.1 Å ×6.7 Å×4.7 Å |
| 51 | Chromafenozide | 99.6 | 10.91±0.20 | 4.4 | 143807-66-3 | C_24_H_30_N_2_O_3_ | 394.51 |  | 10.2 Å ×9.5 Å×8.9 Å |
| 52 | Cinosulfuron | 99.1 | 4.23±0.10 | 1.85 | 94593-91-6 | C_15_H_19_N_5_O_7_S | 413.41 |  | 9.2 Å ×15.0 Å×4.8 Å |
| 53 | Clethodim | 96.5 | 4.28±0.25 | 4.21 | 99129-21-2 | C_17_H_26_ClNO_3_S | 359.91 |  | 12.1 Å ×12.7 Å×5.5 Å |
| 54 | Clethodim sulfone | 99.4 | 4.17±0.25 | 2.19 | 111031-17-5 | C_17_H_26_ClNO_5_S | 391.91 |  | 12.7 Å ×12.2 Å×5.5 Å |
| 55 | Clethodim sulfoxide | 93.4 | 4.22±0.25 | 2.19 | 111031-14-2 | C_17_H_26_ClNO_4_S | 375.91 |  | 10.9 Å ×11.4 Å×5.8 Å |
| 56 | Clofentezine | 99.9 | -1.68±0.31 | 2.70 | 74115-24-5 | C_14_H_8_Cl_2_N_4_ | 303.15 |  | 7.4 Å ×11.6 Å×4.2 Å |
| 57 | Clomazone | 98.2 | -1.48±0.40 | 2.50 | 81777-89-1 | C_12_H_14_ClNO_2_ | 239.7 |  | 9.7 Å ×6.2 Å×4.1 Å |
| 58 | Clothianidin | 99.8 | 2.76±0.50 | 0.64 | 210880-92-5 | C_6_H_8_ClN_5_O_2_S | 249.67 |  | 4.7 Å ×9.6 Å×5.5 Å |
| 59 | Coumaphos | 99.3 | - | 4.47 | 56-72-4 | C_14_H_16_ClO_5_PS | 362.77 |  | 9.0 Å ×11.5 Å×8.2 Å |
| 60 | Coumoxystrobin | 99.1 | - | 5.24 | 850881-70-8 | C_26_H_28_O_6_ | 436.50 |  | 11.1 Å ×15.3 Å×8.5 Å |
| 61 | Cyanazine | 99.9 | 1.46±0.41 | 2.51 | 21725-46-2 | C_9_H_13_ClN_6_ | 240.69 |  | 7.7 Å ×9.5 Å×4.6 Å |
| 62 | Cyantraniliprole | 97.2 | - | 3.43 | 736994-63-1 | C_19_H_14_BrClN_6_O_2_ | 473.71 |  | 7.5 Å ×8.6 Å×7.7 Å |
| 63 | Cyazofamid | 99.0 | -6.61±0.70 | 2.87 | 120116-88-3 | C_13_H_13_ClN_4_O_2_S | 324.79 |  | 9.8 Å ×7.7 Å×3.6 Å |
| 64 | Cyclosulfamuron | 99.9 | 5.04 | 1.88 | 136849-15-5 | C_17_H_19_N_5_O_6_S | 421.43 |  | 10.8 Å ×10.4 Å×8.7 Å |
| 65 | Cycloxydim | 95.5 | 4.32±0.25 | 3.88 | 101205-02-1 | C_17_H_27_NO_3_S | 325.47 |  | 13.4 Å ×8.9 Å×4.9 Å |
| 66 | Cyflufenamid | 99.9 | 9.34±0.46 | 5.60 | 180409-60-3 | C_20_H_17_F_5_N_2_O_2_ | 412.35 |  | 7.1 Å ×7.8 Å×10.0 Å |
| 67 | Cyflumetofen | 99.5 | - | 4.69 | 400882-07-7 | C_24_H_24_F_3_NO_4_ | 447.45 |  | 11.5 Å ×10.6 Å×8.8 Å |
| 68 | Cymoxanil | 99.9 | 9.7 | 4.24 | 57966-95-7 | C_7_H_10_N_4_O_3_ | 198.18 |  | 7.7 Å ×8.5 Å×1.8 Å |
| 69 | Cyproconazole | 99.9 | 12.59±0.29 | 2.90 | 94361-06-5 | C_15_H_18_ClN_3_O | 291.78 |  | 8.3 Å ×9.3 Å×5.1 Å |
| 70 | Deltamethrin | 98.3 | - | 6.18 | 52918-63-5 | C_22_H_19_Br_2_NO_3_ | 505.20 |  | 8.9 Å ×12.8 Å×9.2 Å |
| 71 | Demeton | 99.6 | - | - | 8065-48-3 | C_16_H_38_O_6_P_2_S_4_ | 516.68 |  | 22.8 Å ×8.8 Å×7.2 Å |
| 72 | Demeton-S-methyl | 99.4 | - | 1.01 | 919-86-8 | C_6_H_15_O_3_PS_2_ | 230.29 |  | 5.0 Å ×8.6 Å×8.5 Å |
| 73 | Demeton-S-methyl sulfone | 98.6 | - | -0.91 | 17040-19-6 | C_6_H_15_O_5_PS_2_ | 262.28 |  | 5.9 Å ×7.2 Å×7.8 Å |
| 74 | Demeton-S-sulfone | 98.3 | - | 0.07 | 2496-91-5 | C_8_H_19_O_5_PS_2_ | 290.34 |  | 8.9 Å ×9.6 Å×8.2 Å |
| 75 | Demeton-S-sulfoxide | 98.9 | - | -0.05 | 2496-92-6 | C_8_H_19_O_4_PS_2_ | 274.34 |  | 5.8 Å ×9.8 Å×9.6 Å |
| 76 | Diazinon | 99.6 | 1.21±0.30 | 3.81 | 333-41-5 | C_12_H_21_N_2_O_3_PS | 304.35 |  | 10.0 Å ×8.8 Å×8.0 Å |
| 77 | Dichlorvos | 99.9 | - | 0.60 | 62-73-7 | C_4_H_7_Cl_2_O_4_P | 220.98 |  | 5.7 Å ×5.8 Å×4.8 Å |
| 78 | Diclobutrazol | 99.5 | 13.86±0.20 | 4.01 | 75736-33-3 | C_15_H_19_Cl_2_N_3_O | 328.24 |  | 9.4 Å ×6.8 Å×7.4 Å |
| 79 | Diclofop methyl | 99.2 | - | 4.54 | 51338-27-3 | C_16_H_14_Cl_2_O_4_ | 341.19 |  | 11.4 Å ×5.0 Å×9.6 Å |
| 80 | Dicrotophos | 94.5 | - | -0.10 | 141-66-2 | C_8_H_16_NO_5_P | 237.19 |  | 7.2 Å ×7.9 Å×5.3 Å |
| 81 | Diethofencarb | 98.5 | 12.75±0.70 | 2.91 | 87130-20-9 | C_14_H_21_NO_4_ | 267.32 |  | 6.6 Å ×9.2 Å×9.0 Å |
| 82 | Diethyl aminoethyl hexanoate | 98.8 | 9.25±0.25 | 3.02 | 10369-83-2 | C_12_H_25_NO_2_ | 215.33 |  | 11.4 Å ×8.3 Å×4.4 Å |
| 83 | Difenoconazole | 99.9 | 2.94±0.12 | 4.30 | 119446-68-3 | C_19_H_17_Cl_2_N_3_O_3_ | 406.26 |  | 8.1 Å ×11.5 Å×9.0 Å |
| 84 | Diflubenzuron | 98 | 8.78±0.46 | 3.59 | 35367-38-5 | C_14_H_9_ClF_2_N_2_O_2_ | 310.68 |  | 9.9 Å ×11.2 Å×3.8 Å |
| 85 | Diflufenican | 99.9 | 9.03±0.70 | 3.53 | 83164-33-4 | C_19_H_11_F_5_N_2_O_2_ | 394.29 |  | 9.8 Å ×6.8 Å×10.1 Å |
| 86 | Dimepiperate | 99.3 | -1.24±0.20 | 4.50 | 61432-55-1 | C_15_H_21_NOS | 263.4 |  | 9.5 Å ×5.8 Å×7.8 Å |
| 87 | Dimethenamid | 99.3 | 1.16±0.50 | 2.15 | 87674-68-8 | C_12_H_18_ClNO_2_S | 275.79 |  | 6.7 Å ×5.6 Å×8.5 Å |
| 88 | Dimethoate | 99.9 | 14.40±0.46 | 0.72 | 60-51-5 | C_5_H_12_NO_3_PS_2_ | 229.26 |  | 8.3 Å ×4.5 Å×7.9 Å |
| 89 | Dimethomorph | 99.9 | - | 2.68 | 110488-70-5 | C_21_H_22_ClNO_4_ | 387.86 |  | 8.2 Å ×10.9 Å×10.5 Å |
| 90 | Dimoxystrobin | 99.9 | 11.29±0.46 | 5.36 | 149961-52-4 | C_19_H_22_N_2_O_3_ | 326.39 |  | 9.3 Å ×8.9 Å×7.5 Å |
| 91 | Diniconazole | 99.9 | 12.89±0.20 | 4.30 | 76714-88-0 | C_15_H_17_Cl_2_N_3_O | 326.22 |  | 9.6 Å ×6.9 Å×6.7 Å |
| 92 | Dinocap | 99.9 | 12.7 | 3.3 | 39300-45-3 | C_18_H_24_N_2_O_6_ | 364.39 |  | 7.9 Å ×10.8 Å×8.3 Å |
| 93 | Dinotefuran | 99.9 | 3.24±0.50 | -0.19 | 165252-70-0 | C_7_H_14_N_4_O_3_ | 202.21 |  | 5.7 Å ×8.5 Å×5.3 Å |
| 94 | Disulfoton | 96.7 | - | 4.07 | 298-04-4 | C_8_H_19_O_2_PS_3_ | 274.4 |  | 13.0 Å ×7.5 Å×1.8 Å |
| 95 | Disulfoton sulfone | 99.2 | - | 1.83 | 2497-06-5 | C_8_H_19_O_4_PS_3_ | 306.40 |  | 8.4 Å ×4.0 Å×9.8 Å |
| 96 | Disulfoton sulfoxide | 98.7 | - | 1.72 | 2497-07-6 | C_8_H_19_O_3_PS_3_ | 290.4 |  | 10.1 Å ×8.3 Å×6.7 Å |
| 97 | Diuron | 99.8 | -1 to -2 | 2.68 | 330-54-1 | C_9_H_10_Cl_2_N_2_O | 233.09 |  | 7.2 Å ×5.5 Å×6.6 Å |
| 98 | EPN | 99.5 | - | 4.47 | 2104-64-5 | C_14_H_14_NO_4_PS | 323.3 |  | 10.8 Å ×6.4 Å×6.8 Å |
| 99 | Edifenphos | 99.0 | - | 3.61 | 17109-49-8 | C_14_H_15_O_2_PS_2_ | 310.37 |  | 7.4 Å ×7.4 Å×7.5 Å |
| 100 | Emamectin B1a | 99.4 | - | - | 155569-91-8 | C_49_H_77_NO_13_ | 888.13 |  | 16.7 Å ×14.6 Å×10.3 Å |
| 101 | Enestroburin | 95.0 | - | 5.07 | 238410-11-2 | C_22_H_22_ClNO_4_ | 399.87 |  | 13.3 Å ×9.4 Å×6.8 Å |
| 102 | Epoxiconazole | 99.9 | 2.75±0.10 | 3.44 | 135319-73-2 | C_17_H_13_ClFN_3_O | 329.76 |  | 6.2 Å ×8.0 Å×8.1 Å |
| 103 | Ethion | 98.7 | - | 5.07 | 563-12-2 | C_9_H_22_O_4_P_2_S_4_ | 384.46 |  | 9.2 Å ×10.7 Å×7.2 Å |
| 104 | Ethiprole | 99.8 | - | 5.23 | 181587-01-9 | C_13_H_9_Cl_2_F_3_N_4_OS | 397.20 |  | 8.6 Å ×10.8 Å×4.5 Å |
| 105 | Ethofumesate | 98.5 | - | 2.89 | 26225-79-6 | C_13_H_18_O_5_S | 286.34 |  | 10.2 Å ×4.9 Å×9.5 Å |
| 106 | Ethoprophos | 99.7 | - | 3.14 | 13194-48-4 | C_8_H_19_O_2_PS_2_ | 242.34 |  | 8.5 Å ×6.5 Å×8.0 Å |
| 107 | Ethoxysulfuron | 99.4 | 5.28 | 2.02 | 126801-58-9 | C_15_H_18_N_4_O_7_S | 398.39 |  | 8.3 Å ×10.4 Å×8.5 Å |
| 108 | Etofenprox | 99.0 | - | 7.47 | 80844-07-1 | C_25_H_28_O_3_ | 376.49 |  | 10.9 Å ×10.6 Å×10.1 Å |
| 109 | Etoxazole | 99.1 | 2.04±0.70 | 7.21 | 153233-91-1 | C_21_H_23_F_2_NO_2_ | 359.41 |  | 12.1 Å ×9.4 Å×8.6 Å |
| 110 | Etrimfos | 94.3 | -0.67±0.32 | 2.94 | 38260-54-7 | C_10_H_17_N_2_O_4_PS | 292.29 |  | 10.0 Å ×7.3 Å×6.7 Å |
| 111 | Famoxadone | 99.4 | 0.63±0.40 | 4.65 | 131807-57-3 | C_22_H_18_N_2_O_4_ | 374.39 |  | 12.5 Å ×9.9 Å×10.1 Å |
| 112 | Fenamidone | 99.9 | 0.49±0.40 | 3.45 | 161326-34-7 | C_17_H_17_N_3_OS | 311.4 |  | 6.6 Å ×8.7 Å×9.0 Å |
| 113 | Fenaminstrobin | 99.9 | 11.27±0.46 | 6.54 | 366815-39-6 | C_21_H_21_Cl_2_N_3_O_3_ | 434.32 |  | 13.5 Å ×9.5 Å×8.5 Å |
| 114 | Fenamiphos | 99.0 | - | 3.23 | 22224-92-6 | C_13_H_22_NO_3_PS | 303.36 |  | 11.5 Å ×5.5 Å×8.4 Å |
| 115 | Fenamiphos sulphone | 99.4 | -0.48±0.70 | 1.26 | 31972-44-8 | C_13_H_22_NO_5_PS | 335.36 |  | 11.4 Å ×6.0 Å×9.3 Å |
| 116 | Fenamiphos sulphoxide | 98.6 | -0.34±0.70 | 1.13 | 31972-43-7 | C_13_H_22_NO_4_PS | 319.36 |  | 12.0 Å ×5.5 Å×9.3 Å |
| 117 | Fenarimol | 99.2 | - | 3.62 | 60168-88-9 | C_17_H_12_Cl_2_N_2_O | 331.2 |  | 6.1 Å ×8.5 Å×8.4 Å |
| 118 | Fenazaquin | 99.9 | 2.88±0.30 | 5.76 | 120928-09-8 | C_20_H_22_N_2_O | 306.4 |  | 15.8 Å ×6.5 Å×4.0 Å |
| 119 | Fenbuconazole | 99.8 | 2.34±0.10 | 3.23 | 114369-43-6 | C_19_H_17_ClN_4_ | 336.82 |  | 9.5 Å ×8.7 Å×7.9 Å |
| 120 | Fenhexamid | 99.9 | 7.73±0.36 | 3.72 | 126833-17-8 | C_14_H_17_Cl_2_NO_2_ | 302.2 |  | 7.5 Å ×6.1 Å×6.5 Å |
| 121 | Fenobucarb | 99.2 | 12.24±0.46 | 2.86 | 3766-81-2 | C_12_H_17_NO_2_ | 207.27 |  | 5.0 Å ×8.9 Å×5.8 Å |
| 122 | Fenothiocarb | 99.9 | -1.21±0.70 | 3.28 | 62850-32-2 | C_13_H_19_NO_2_S | 253.36 |  | 7.8 Å ×9.3 Å×12.6 Å |
| 123 | Fenoxanil | 99.9 | 11.44±0.46 | 4.25 | 115852-48-7 | C_15_H_18_Cl_2_N_2_O_2_ | 329.22 |  | 8.8 Å ×7.2 Å×7.3 Å |
| 124 | Fenoxaprop-ethyl | 99.3 | -0.08±0.30 | 4.95 | 71283-80-2 | C_18_H_16_ClNO_5_ | 361.78 |  | 7.6 Å ×12.6 Å×7.3 Å |
| 125 | Fenoxycarb | 99.9 | - | 4.24 | 79127-80-3 | C_17_H_19_NO_4_ | 301.34 |  | 8.0 Å ×9.1 Å×7.5 Å |
| 126 | Fenpropathrin | 99.2 | - | 5.62 | 64257-84-7 | C_22_H_23_NO_3_ | 349.43 |  | 6.8 Å ×9.0 Å×10.6 Å |
| 127 | Fenpyrazamine | 99.7 | 3.22±0.20 | 1.81 | 473798-59-3 | C_17_H_21_N_3_O_2_S | 331.43 |  | 6.8 Å ×12.0 Å×6.3 Å |
| 128 | Fenpyroximate | 99.6 | 1.53±0.10 | 5.57 | 111812-58-9 | C_24_H_27_N_3_O_4_ | 421.49 |  | 16.1 Å ×10.8 Å×6.6 Å |
| 129 | Fensulfothion | 98.8 | - | 2.35 | 115-90-2 | C_11_H_17_O_4_PS_2_ | 308.35 |  | 10.0 Å ×6.9 Å×7.9 Å |
| 130 | Fensulfothion oxon | 99.4 | - | 0.59 | 6552-21-2 | C_11_H_17_O_5_PS | 292.29 |  | 9.5 Å ×8.4 Å×9.1 Å |
| 131 | Fensulfothion oxon sulfone | 99.8 | - | 0.72 | 6132-17-8 | C_11_H_17_O_6_PS | 308.29 |  | 7.0 Å ×8.4 Å×9.3 Å |
| 132 | Fenthion | 95.2 | - | 4.09 | 55-38-9 | C_10_H_15_O_3_PS_2_ | 278.32 |  | 8.3 Å ×6.2 Å×7.7 Å |
| 133 | Fenthion sulphone | 99.1 | - | 2.05 | 3761-42-0 | C_10_H_15_O_5_PS_2_ | 310.33 |  | 9.3 Å ×8.3 Å×7.5 Å |
| 134 | Fenthion sulfoxide | 99.1 | - | 1.92 | 3761-41-9 | C_10_H_15_O_4_PS_2_ | 294.33 |  | 9.1 Å ×6.9 Å×7.9 Å |
| 135 | Fenvalerate | 96.4 | - | 6.76 | 51630-58-1 | C_25_H_22_ClNO_3_ | 419.90 |  | 14.9 Å ×5.9 Å×11.4 Å |
| 136 | Fipronil | 99.8 | -5.86±0.20 | 4.0 | 120068-37-3 | C_12_H_4_Cl_2_F_6_N_4_OS | 437.15 |  |  |
| 137 | Fipronil desulfinyl | 99.8 | -4.15±0.20 | 4.83 | 205650-65-3 | C_12_H_4_Cl_2_F_6_N_4_ | 389.08 |  |  |
| 138 | Fipronil sulphide | 99.8 | -4.16±0.20 | 6.01 | 120067-83-6 | C_12_H_4_Cl_2_F_6_N_4_S | 421.15 |  |  |
| 139 | Fipronil sulphone | 99.8 | 8.12±0.20 | 4.79 | 120068-36-2 | C_12_H_4_C_l2_F_6_N_4_O_2_S | 453.15 |  |  |
| 140 | Flonicamid | 99.9 | 10.99±0.46 | 0.50 | 158062-67-0 | C_9_H_6_F_3_N_3_O | 229.16 |  | 5.9 Å ×8.3 Å×4.9 Å |
| 141 | Florasulam | 99.9 | 4.54 | 2.13 | 145701-23-1 | C_12_H_8_F_3_N_5_O_3_S | 359.28 |  | 10.0 Å ×8.3 Å×8.5 Å |
| 142 | Fluazifop-butyl | 96.9 | 0.78±0.22 | 5.34 | 69806-50-4 | C_19_H_20_F_3_NO_4_ | 383.36 |  | 6.0 Å ×12.0 Å×15.9 Å |
| 143 | Fluazinam |  | 7.11 | 5.70 | 79622-59-6 | C_13_H_4_Cl_2_F_6_N_4_O_4_ | 465.09 |  |  |
| 144 | Flubendiamide |  | 11.59±0.70( | 4.80 | 272451-65-7 | C_23_H_22_F_7_IN_2_O_4_S | 682.39 |  |  |
| 145 | Flucetosulfuron | 99.6 | 3.5 | 0.22 | 412928-75-7 | C_18_H_22_FN_5_O_8_S | 487.46 |  | 12.5 Å ×7.6 Å×6.9 Å |
| 146 | Flucythrinate | 99.9 | - | 6.20 | 70124-77-5 | C_26_H_23_F_2_NO_4_ | 451.5 |  | 12.9 Å×13.0 Å×7.5 Å |
| 147 | Fludioxonil | 99.9 | 14.10±0.50 | 3.83 | 131341-86-1 | C_12_H_6_F_2_N_2_O_2_ | 248.18 |  | 7.2 Å ×8.1 Å×3.8 Å |
| 148 | Flufenacet | 99.9 | 0.31±0.50 | 3.20 | 142459-58-3 | C_14_H_13_F_4_N_3_O_2_S | 363.33 |  | 11.2 Å ×6.7 Å×7.3 Å |
| 149 | Flufenoxuron | 99.9 | 8.68±0.46 | 5.97 | 101463-69-8 | C_21_H_11_ClF_6_N_2_O_3_ | 488.77 |  | 10.7 Å ×9.5 Å×8.3 Å |
| 150 | Flumetralin | 99.9 | -2.63±0.50 | 6.09 | 62924-70-3 | C_16_H_12_ClF_4_N_3_O_4_ | 421.73 |  | 8.9 Å ×7.9 Å×6.6 Å |
| 151 | Flumetsulam | 99.9 | 4.6 | 1.50 | 98967-40-9 | C_12_H_9_F_2_N_5_O_2_S | 325.29 |  | 8.9 Å ×7.9 Å×7.6 Å |
| 152 | Flumorph | 96.5 | -1.20±0.20 | 1.92 | 211867-47-9 | C_21_H_22_FNO_4_ | 371.40 |  | 9.0 Å ×10.7 Å×8.4 Å |
| 153 | Fluopicolide | 98.2 | - | 4.62 | 239110-15-7 | C_14_H_8_Cl_3_F_3_N_2_O | 383.58 |  | 8.0 Å ×7.4 Å×10.4 Å |
| 154 | Fluopyram | 99.9 | - | 4.78 | 658066-35-4 | C_16_H_11_ClF_6_N_2_O | 396.71 |  | 8.1 Å ×9.3 Å×12.3 Å |
| 155 | Fluoroglycofen-ethyl | 99.9 | - | 4.50 | 77501-90-7 | C_18_H_13_ClF_3_NO_7_ | 447.75 |  | 13.8 Å ×8.5 Å×5.3 Å |
| 156 | Flurtamone | 99.9 | 2.97±0.40 | 2.87 | 96525-23-4 | C_18_H_14_F_3_NO_2_ | 333.31 |  | 9.3 Å ×10.0 Å×5.8 Å |
| 157 | Flusilazole | 99.7 | 2.5 | 3.70 | 85509-19-9 | C_16_H_15_F_2_N_3_Si | 315.40 |  | 8.8 Å ×8.2 Å×6.7 Å |
| 158 | Fluthiacet-methyl | 99.9 | -2.86±0.20 | 2.59 | 117337-19-6 | C_15_H_15_ClFN_3_O_3_S_2_ | 403.88 |  | 9.9 Å ×8.7 Å×8.9 Å |
| 159 | Flutolanil | 99.8 | 12.44±0.70 | 3.70 | 66332-96-5 | C_17_H_16_F_3_NO_2_ | 323.32 |  | 7.4 Å ×8.0 Å×8.1 Å |
| 160 | Flutriafol | 99.2 | 11.60±0.29 | 2.29 | 76674-21-0 | C_16_H_13_F_2_N_3_O | 301.30 |  | 7.4 Å ×7.3 Å×5.8 Å |
| 161 | Fluvalinate | 96.3 | -1.16±0.50 | 6.81 | 69409-94-5 | C_26_H_22_ClF_3_N_2_O_3_ | 502.91 |  | 9.9 Å ×9.2 Å×12.9 Å |
| 162 | Fluxapyroxad | 98.1 | 11.73±0.70 | 3.47 | 907204-31-3 | C_18_H_12_F_5_N_3_O | 381.31 |  | 5.8 Å ×12.1 Å×6.1 Å |
| 163 | Fonofos | 98.9 | - | 4.02 | 994-22-9 | C_10_H_15_OPS_2_ | 246.32 |  | 5.4 Å ×9.1 Å×7.7 Å |
| 164 | Forchlorfenuron | 99.5 | 12.55±0.70 | 2.42 | 68157-60-8 | C_12_H_10_ClN_3_O | 247.68 |  | 7.9 Å ×9.1 Å×7.7 Å |
| 165 | Formothion | 97.0% | -1.79±0.70 | 0.45 | 2540-82-1 | C_6_H_12_NO_4_PS_2_ | 257.27 |  | 7.0 Å ×8.7 Å×4.0 Å |
| 166 | Fosthiazate | 99.8 | - | 2.47 | 98886-44-3 | C_9_H_18_NO_3_PS_2_ | 283.34 |  | 7.1 Å ×7.4 Å×5.9 Å |
| 167 | Furathiocarb | 98 | -2.17±0.70 | 4.09 | 65907-30-4 | C_18_H_26_N_2_O_5_S | 382.48 |  | 18.2 Å ×7.4 Å×7.2 Å |
| 168 | Heptenophos | 98.5 | - | 1.41 | 23560-59-0 | C_9_H_12_ClO_4_P | 250.62 |  | 8.4 Å ×5.4 Å×4.7 Å |
| 169 | Hexaconazole | 98.8 | 12.26±0.29 | 3.66 | 79983-71-4 | C_14_H_17_Cl_2_N_3_O | 314.21 |  | 6.6 Å ×6.7 Å×9.4 Å |
| 170 | Hexaflumuron | 99.1 | 8.54±0.46 | 5.64 | 86479-06-3 | C_16_H_8_Cl_2_F_6_N_2_O_3_ | 461.14 |  | 10.0 Å ×8.0 Å×5.2 Å |
| 171 | Hexazinone | 99.7 | 2.05±0.70 | 2.15 | 51235-04-2 | C_12_H_20_N_4_O_2_ | 252.31 |  | 7.1 Å ×5.7 Å×9.2 Å |
| 172 | Hexythiazox | 99.8 | 12.77±0.20 | 5.57 | 78587-05-0 | C_17_H_21_ClN_2_O_2_S | 352.88 |  | 7.9 Å ×6.7 Å×8.0 Å |
| 173 | Imazalil | 99.3 | 6.53 | 3.82 | 35554-44-0 | C_14_H_14_Cl_2_N_2_O | 297.18 |  | 6.9 Å ×10.0 Å×6.0 Å |
| 174 | Imidacloprid | 99.9 | 7.16±0.20 | -0.41 | 138261-41-3 | C_9_H_10_ClN_5_O_2_ | 255.66 |  | 8.0 Å ×4.9 Å×7.7 Å |
| 175 | Imidaclothiz | 98.0 | 7.15±0.20 | -0.23 | 105843-36-5 | C_7_H_8_ClN_5_O_2_S | 261.69 |  | 3.0 Å ×6.1 Å×7.9 Å |
| 176 | Indoxacarb | 98.0 | -1.75±0.40 | 4.60 | 144171-61-9 | C_22_H_17_ClF_3_N_3_O_7_ | 527.84 |  | 10.5 Å ×12.3 Å×7.3 Å |
| 177 | Iodosulfuron-methyl | 99.4 | 2.31±0.10 | 2.32 | 185119-76-0 | C_13_H1_2_IN_5_O_6_S | 493.23 |  | 8.8 Å ×8.2 Å×5.3 Å |
| 178 | Ipconazole | 98 | 13.76±0.60 | 4.65 | 125225-28-7 | C_18_H_24_ClN_3_O | 333.86 |  | 7.4 Å ×6.5 Å×10.8 Å |
| 179 | Iprobenfos | 99.5 | - | 3.57 | 26087-47-8 | C_13_H_21_O_3_PS | 288.34 |  | 9.4 Å ×7.7 Å×8.3 Å |
| 180 | Iprodione | 99 | 9.19±0.20 | 2.85 | 36734-19-7 | C_13_H_13_Cl_2_N_3_O_3_ | 330.17 |  | 7.5 Å ×12.0 Å×5.6 Å |
| 181 | Iprovalicarb | 98.1 | 11.41±0.46 | 3.33 | 140923-17-7 | C_18_H_28_N_2_O_3_ | 320.43 |  | 8.5 Å ×12.3 Å×7.0 Å |
| 182 | Isazofos | 99.4 | -1.47±0.10 | 3.71 | 42509-80-8 | C_9_H_17_ClN_3_O_3_PS | 313.74 |  | 9.8 Å ×9.3 Å×6.9 Å |
| 183 | Isocarbofos | 99.8 | - | 2.71 | 24353-61-5 | C_11_H_16_NO_4_PS | 289.29 |  | 7.2 Å ×6.1 Å×6.1 Å |
| 184 | Isofenphos-methyl | 97.3 | - | 3.91 | 99675-03-3 | C_14_H_22_NO_4_PS | 331.37 |  | 7.0 Å ×8.3 Å×7.7 Å |
| 185 | Isoprocarb | 98.9 | 12.22±0.46 | 2.37 | 2631-40-5 | C_11_H_15_NO_2_ | 193.24 |  | 5.5 Å ×6.8 Å×7.8 Å |
| 186 | Isoprothiolane | 85.2 | - | 2.88 | 50512-35-1 | C_12_H_18_O_4_S_2_ | 290.40 |  | 8.8 Å ×5.8 Å×9.1 Å |
| 187 | Isoproturon | 99.1 | 15.06±0.70 | 2.87 | 34123-59-6 | C_12_H_18_N_2_O | 206.29 |  | 8.7 Å ×7.3 Å×6.2 Å |
| 188 | Isopyrazam | 99.6 | 12.27±0.40 | 4.42 | 881685-58-1 | C_20_H_23_F_2_N_3_O | 359.41 |  | 9.8 Å ×7.2 Å×9.4 Å |
| 189 | Isoxaflutole | 99.9 | -4.29±0.50 | 2.32 | 141112-29-0 | C_15_H_12_F_3_NO_4_S | 359.32 |  | 8.6 Å ×7.7 Å×8.0 Å |
| 190 | Ivermectin B1a | 97.0 | 12.42±0.70 | 4.61 | 71827-03-7 | C_48_H_74_O_14_ | 875.09 |  | 11.8 Å ×15.6 Å×10.8 Å |
| 191 | Kresoxim-methyl | 99.9 | - | 5.88 | 143390-89-0 | C_18_H_19_NO_4_ | 313.35 |  | 11.2 Å ×7.7 Å×6.8 Å |
| 192 | Lactofen | 99 | - | 6.61 | 77501-63-4 | C_19_H_15_ClF_3_NO_7_ | 461.77 |  | 13.5 Å ×8.5 Å×6.3 Å |
| 193 | Linuron | 99.7 | 12.13±0.70 | 2.91 | 330-55-2 | C_9_H_10_Cl_2_N_2_O_2_ | 249.09 |  | 7.7 Å ×5.4 Å×6.7 Å |
| 194 | Lufenuron | 99.0 | 8.49±0.46 | 6.61 | 103055-07-8 | C_17_H_8_Cl_2_F_8_N_2_O_3_ | 511.15 |  | 11.0 Å ×8.4 Å×6.3 Å |
| 195 | Malaoxon | 97.2 | - | 0.52 | 1634-78-2 | C_10_H_19_O_7_PS | 314.29 |  | 10.1 Å ×8.7 Å×7.4 Å |
| 196 | Malathion | 98.7 | - | 2.36 | 121-75-5 | C_10_H_19_O_6_PS_2_ | 330.35 |  | 5.7 Å ×10.2 Å×8.3 Å |
| 197 | Mandipropamid | 99.4 | - | 3.57 | 374726-62-2 | C_23_H_22_ClNO_4_ | 411.88 |  | 12.8 Å ×10.1 Å×11.5 Å |
| 198 | Mefenacet | 99.9 | 1.53±0.10 | 2.80 | 73250-68-7 | C_16_H_14_N_2_O_2_S | 298.36 |  | 11.5 Å ×6.3 Å×9.8 Å |
| 199 | Mepronil | 99.5 | 13.10±0.70 | 4.24 | 55814-41-0 | C_17_H_19_NO_2_ | 269.34 |  | 4.8 Å ×5.2 Å×12.2 Å |
| 200 | Metaflumizone | 99.6 | 10.82±0.46 | 7.72 | 139968-49-3 | C_24_H_16_F_6_N_4_O_2_ | 506.4 |  | 12.3 Å ×11.9 Å×5.4 Å |
| 201 | Metalaxyl | 98.7 | 1.41±0.50 | 1.65 | 57837-19-1 | C_15_H_21_NO_4_ | 279.34 |  | 6.6 Å ×7.7 Å×10.0 Å |
| 202 | Metamifop | 98.3 | 0.16±0.50 | 5.21 | 256412-89-2 | C_23_H_18_ClFN_2_O_4_ | 440.85 |  | 11.9 Å ×14.3 Å×6.7 Å |
| 203 | Metamitron | 99.3 | 1.54±0.20 | 1.44 | 41394-05-2 | C_10_H_10_N_4_O | 202.21 |  | 3.1 Å ×5.2 Å×9.7 Å |
| 204 | Metazachlor | 99.9 | 1.54±0.10 | 2.13 | 67129-08-2 | C_14_H_16_ClN_3_O | 277.75 |  | 6.0 Å ×8.9 Å×5.9 Å |
| 205 | Metconazole | 99.9 | 13.82±0.60 | 3.93 | 125116-23-6 | C_17_H_22_ClN_3_O | 319.83 |  | 7.8 Å ×9.2 Å×7.9 Å |
| 206 | Methacrifos | 98 | - | 2.53 | 62610-77-9 | C_7_H_13_O_5_PS | 240.21 |  | 8.3 Å ×6.8 Å×1.8 Å |
| 207 | Methamidophos | 99.2 | - | -0.93 | 10265-92-6 | C_2_H_8_NO_2_PS | 141.13 |  | 4.3 Å ×4.7 Å×5.1 Å |
| 208 | Methidathion | 99 | -4.17±0.40 | 2.20 | 950-37-8 | C_6_H_11_N_2_O_4_PS_3_ | 302.32 |  | 7.4 Å ×8.5 Å×6.8 Å |
| 209 | Methiocarb | 95.0 | 12.16±0.46 | 2.92 | 2032-65-7 | C_11_H_15_NO_2_S | 225.31 |  | 5.0 Å ×5.8 Å×9.2 Å |
| 210 | Methiocarb sulfone | 98.6 | 11.96±0.46 | 0.84 | 2179-25-1 | C_11_H_15_NO_4_S | 257.31 |  | 8.6 Å ×7.5 Å×4.8 Å |
| 211 | Methiocarb sulfoxide | 99.6 | 12.03±0.46 | 0.70 | 2635-10-1 | C_11_H_15_NO_3_S | 241.31 |  | 8.2 Å ×8.1 Å×4.7 Å |
| 212 | Methomyl | 99.2 | - | 0.61 | 16752-77-5 | C_5_H_10_N_2_O_2_S | 162.21 |  | 5.9 Å ×6.0 Å×1.8 Å |
| 213 | Methoxyfenozide | 98.7 | 10.43±0.4 | 3.70 | 161050-58-4 | C_22_H_28_N_2_O_3_ | 368.48 |  | 8.8 Å ×6.7 Å×10.0 Å |
| 214 | Metolachlor | 99.2 | 1.45±0.50 | 3.24 | 87392-12-9 | C_15_H_22_ClNO_2_ | 283.79 |  | 7.8 Å ×7.2 Å×6.9 Å |
| 215 | Metolcarb | 99.8 | 12.38±0.46 | 1.72 | 1129-41-5 | C_9_H_11_NO_2_ | 165.19 |  | 4.9 Å ×6.8 Å×6.6 Å |
| 216 | Metrafenone | 97.4 | - | 4.72 | 220899-03-6 | C_19_H_21_BrO_5_ | 409.28 |  | 8.6 Å ×8.7 Å×8.4 Å |
| 217 | Metribuzin | 98 | -0.16±0.20 | 1.70 | 21087-64-9 | C_8_H_14_N_4_OS | 214.29 |  | 6.6 Å ×5.9 Å×6.8 Å |
| 218 | Metsulfuron-methyl | 99.3 | 2.55±0.10 | 2.20 | 74223-64-6 | C_14_H_15_N_5_O_6_S | 381.36 |  | 7.5 Å ×7.7 Å×8.2 Å |
| 219 | Mevinphos | 97.9 | - | 0.76 | 26718-65-0 | C_7_H_13_O_6_P | 224.15 |  | 4.2 Å ×4.6 Å×10.6 Å |
| 220 | Molinate | 99.4 | -1.22±0.20 | 2.91 | 2212-67-1 | C_9_H_17_NOS | 187.30 |  | 7.9 Å ×5.3 Å×6.6 Å |
| 221 | Monocrotophos | 97.2 | 13.98±0.46 | -0.31 | 2157-98-4 | C_7_H_14_NO_5_P | 223.16 |  | 5.5 Å ×8.2 Å×6.3 Å |
| 222 | Myclobutanil | 99.9% | 2.30±0.10 | 2.94 | 88671-89-0 | C_15_H_17_ClN_4_ | 288.78 |  | 10.7 Å ×7.7 Å×4.5 Å |
| 223 | Napropamide | 99.9 | -0.82±0.70 | 3.33 | 15299-99-7 | C_17_H_21_NO_2_ | 271.35 |  | 10.5 Å ×7.1 Å×7.1 Å |
| 224 | Nitenpyram | 98.8 | 2.46±0.70 | 0.40 | 150824-47-8 | C_11_H_15_ClN_4_O_2_ | 270.72 |  | 6.5 Å ×7.7 Å×7.6 Å |
| 225 | Novaluron | 99 | 8.68±0.46 | 5.27 | 116714-46-6 | C_17_H_9_ClF_8_N_2_O_4_ | 492.71 |  | 11.9 Å ×7.4 Å×14.6 Å |
| 226 | Omethoate | 99.2 | - | -0.79 | 1113-02-6 | C_5_H_12_NO_4_PS | 213.19 |  | 4.8 Å ×7.6 Å×7.5 Å |
| 227 | Orthosulfamuron | 99.2 | - | 0.14 | 213464-77-8 | C_16_H_20_N_6_O_6_S | 424.43 |  | 9.4 Å ×9.8 Å×8.3 Å |
| 228 | Oxadiargyl | 98.9 | -2.99±0.40 | 3.95 | 39807-15-3 | C_15_H_14_Cl_2_N_2_O_3_ | 341.19 |  | 8.7 Å ×9.7 Å×17.6 Å |
| 229 | Oxadiazon | 99.9 | -2.73±0.40 | 4.81 | 19666-30-9 | C_15_H_18_Cl_2_N_2_O_3_ | 345.22 |  | 9.2 Å ×7.7 Å×8.7 Å |
| 230 | Oxadixyl | 99.9 | 1.16±0.20 | 0.8 | 77732-09-3 | C_14_H_18_N_2_O_4_ | 278.31 |  | 6.6 Å ×6.4 Å×8.1 Å |
| 231 | Oxamyl | 99.4 | - | -0.47 | 23135-22-0 | C_7_H_13_N_3_O_3_S | 219.26 |  | 4.6 Å ×9.2 Å×5.6 Å |
| 232 | Oxamyl-oxime | 99.9 | 9.39±0.10 | -0.71 | 30558-43-1 | C_5_H_10_N_2_O_2_S | 162.21 |  | 2.5 Å ×6.1 Å×5.9 Å |
| 233 | Oxaziclomefone | 99.9 | -2.24±0.60 | 5.15 | 153197-14-9 | C_20_H_19_Cl_2_NO_2_ | 376.28 |  | 7.8 Å ×7.4 Å×11.5 Å |
| 234 | Oxydemeton-methyl | 98.9 | - | -0.74 | 301-12-2 | C_6_H_15_O_4_PS_2_ | 246.28 |  | 7.4 Å ×10.7 Å×7.2 Å |
| 235 | Oxyfluorfen | 99.6 | - | 5.21 | 42874-03-3 | C_15_H_11_ClF_3_NO_4_ | 361.7 |  | 5.7 Å ×11.3 Å×7.6 Å |
| 236 | Paclobutrazol | 99.1 | 13.92±0.20 | 3.36 | 76738-62-0 | C_15_H_20_ClN_3_O | 293.80 |  | 7.7 Å ×6.9 Å×7.8 Å |
| 237 | Parathion | 99.8 | - | 3.73 | 56-38-2 | C_10_H_14_NO_5_PS | 291.26 |  | 8.3 Å ×6.0 Å×7.3 Å |
| 238 | Penconazole | 99.0 | 2.80±0.10 | 4.4 | 66246-88-6 | C_13_H_15_Cl_2_N_3_ | 284.18 |  | 10.9 Å ×7.9 Å×7.31Å |
| 239 | Pencycuron | 97.0 | 14.80±0.70 | 4.8 | 66063-05-6 | C_19_H_21_ClN_2_O | 328.84 |  | 7.7 Å ×8.7 Å×9.8Å |
| 240 | Pendimethalin | 99.4 | -2.24±0.33 | 2.62 | 40487-42-1 | C_13_H_19_N_3_O_4_ | 281.31 |  | 7.8 Å ×6.8 Å×5.8 Å |
| 241 | Penflufen | 99.9 | 11.69±0.70 | 4.35 | 494793-67-8 | C_18_H_24_FN_3_O | 317.40 |  | 6.2 Å ×7.9 Å×8.1 Å |
| 242 | Penoxsulam | 99.6 | 5.1 | 2.95 | 219714-96-2 | C_16_H_14_F_5_N_5_O_5_S | 483.37 |  | 11.2 Å ×6.6 Å×6.9 Å |
| 243 | Penthiopyrad | 99.9 | 12.77±0.70 | 4.95 | 183675-82-3 | C_16_H_20_F_3_N_3_OS | 359.41 |  | 5.6 Å ×9.2 Å×9.1 Å |
| 244 | Permethrin | 99.2 | - | 7.43 | 52645-53-1 | C_21_H_20_Cl_2_O_3_ | 391.29 |  | 8.6 Å ×12.7 Å×5.6 Å |
| 245 | Phenamacril | 98.4 | - | 1.63 | 3336-69-4 | C_12_H_12_N_2_O_2_ | 216.24 |  | 6.9 Å ×8.6 Å×5.8 Å |
| 246 | Phenmedipham | 99.7 | 13.03±0.70 | 3.59 | 13684-63-4 | C_16_H_16_N_2_O_4_ | 300.31 |  | 9.9 Å ×7.7 Å×7.0 Å |
| 247 | Phenthoate | 98.2 | - | 3.47 | 2597-03-7 | C_12_H_17_O_4_PS_2_ | 320.36 |  | 11.2 Å ×6.4 Å×7.6 Å |
| 248 | Phorate | 99.0 | - | 3.3 | 298-02-2 | C_7_H_17_O_2_PS_3_ | 260.377 |  | 11.3 Å ×5.6 Å×7.9Å |
| 249 | Phorate sulfone | 99.9 | - | 1.94 | 2588-04-7 | C_7_H_17_O_4_PS_3_ | 292.38 |  | 6.8 Å ×7.0 Å×10.3 Å |
| 250 | Phorate sulfoxide | 99.0 | - | 0.58 | 2588-05-8 | C_7_H_17_O_4_PS_2_ | 260.31 |  | 9.1 Å ×6.2 Å×7.5 Å |
| 251 | Phosalone | 98.3 | -1.75±0.20 | 3.68 | 2310-17-0 | C_12_H_15_ClNO_4_PS_2_ | 367.80 |  | 5.2 Å ×12.7 Å×8.9 Å |
| 252 | Phosfolan | 98 | - | 1.17 | 947-02-4 | C_7_H_14_NO_3_PS_2_ | 255.29 |  | 7.6 Å ×6.9 Å×6.5 Å |
| 253 | Phosfolan methyl | 99.8 | - | 0.19 | 5120-23-0 | C_5_H_10_NO_3_PS_2_ | 227.24 |  | 6.3 Å ×4.8 Å×8.1 Å |
| 254 | Phosmet | 99.9 | -2.63±0.20 | 2.78 | 732-11-6 | C_11_H_12_NO_4_PS_2_ | 317.31 |  | 9.2 Å ×8.4 Å×8.3 Å |
| 255 | Phosmet-Oxon | 99.7 | - | 2.92 | 3785-33-9 | C_10_H_11_BrO_2_ | 243.10 |  | 9.6 Å ×7.4 Å×2.3 Å |
| 256 | Phosphamidon | 98.0 | - | 1.38 | 13171-21-6 | C_10_H_19_ClNO_5_P | 299.69 |  | 7.0 Å ×8.3 Å×9.2 Å |
| 257 | Phoxim | 98.0 | - | 4.17 | 14816-18-3 | C_12_H_15_N_2_O_3_PS | 298.29 |  | 7.5 Å ×6.5 Å×8.7 Å |
| 258 | Picolinafen | 99.9 | 10.28±0.70 | 5.37 | 137641-05-5 | C_19_H_12_F_4_N_2_O_2_ | 376.3 |  | 9.8 Å ×10.7 Å×5.6 Å |
| 259 | Picoxystrobin | 99.7 | -1.09±0.24 | 3.67 | 117428-22-5 | C_18_H_16_F_3_NO_4_ | 367.32 |  | 6.9 Å ×10.5 Å×7.5 Å |
| 260 | Pirimicarb | 99.9 | 4.34 | 1.7 | 23103-98-2 | C_11_H_18_N_4_O_2_ | 238.29 |  | 4.6 Å ×7.5 Å×8.5 Å |
| 261 | Piperonyl butoxide | 98 | - | 4.750 | 51-03-6 | C_19_H_30_O_5_ | 338.44 |  | 17.7 Å ×7.3 Å×8.5 Å |
| 262 | Pirimicarb desmethyl | 99.7 | - | 0.85 | 30614-22-3 | C_10_H_16_N_4_O_2_ | 224.26 |  | 6.0 Å ×7.6 Å×8.7 Å |
| 263 | Pirimiphos-methyl | 99.4 | 3.71 | 4.20 | 29232-93-7 | C_11_H_20_N_3_O_3_PS | 305.33 |  | 5.2 Å ×9.0 Å×7.9 Å |
| 264 | Pretilachlor | 98 | 1.41±0.50 | 4.29 | 51218-49-6 | C_17_H_26_ClNO_2_ | 311.85 |  | 6.5 Å ×7.8 Å×12.3 Å |
| 265 | Probenazole | 99.3 | -2.43±0.20 | 2.89 | 27605-76-1 | C_10_H_9_NO_3_S | 223.25 |  | 7.4 Å ×6.6 Å×7.6 Å |
| 266 | Prochloraz | 99.9 | 3.8 | 4.13 | 67747-09-5 | C_15_H_16_Cl_3_N_3_O_2_ | 376.67 |  | 7.1 Å ×12.9 Å×5.9 Å |
| 267 | Procymidone | 99.9 | -2.67±0.60 | 2.59 | 32809-16-8 | C_13_H_11_Cl_2_NO_2_ | 284.14 |  | 8.9 Å ×5.3 Å×3.9 Å |
| 268 | Profenofos | 99.4 | - | 4.68 | 41198-08-7 | C_11_H_15_BrClO_3_PS | 373.63 |  | 7.7 Å ×7.3 Å×8.5 Å |
| 269 | Promecarb | 99.7 | 12.37±0.46 | 3.1 | 2631-37-0 | C_12_H_17_NO_2_ | 207.27 |  | 5.9 Å ×6.5 Å×8.4 Å |
| 270 | Prometryn | 99.8 | 3.76±0.41 | 3.73 | 7287-19-6 | C_10_H_19_N_5_S | 241.36 |  | 5.9 Å ×8.5 Å×8.1 Å |
| 271 | Propachlor | 98.7 | 0.30±0.50 | 2.18 | 1918-16-7 | C_11_H_14_ClNO | 211.69 |  | 7.2 Å ×7.7 Å×4.8 Å |
| 272 | Propamocarb | 99.7 | 12.73±0.46 | 1.12 | 24579-73-5 | C_9_H_20_N_2_O_2_ | 188.27 |  | 7.8 Å ×8.0 Å×8.6 Å |
| 273 | Propanil | 99.8 | 13.58±0.70 | 3.070 | 709-98-8 | C_9_H_9_Cl_2_NO | 218.08 |  | 6.3 Å ×6.6 Å×2.5 Å |
| 274 | Propaquizafop | 98.0 | -1.41±0.48 | 4.59 | 111479-05-1 | C_22_H_22_ClN_3_O_5_ | 443.88 |  | 16.1 Å ×9.7 Å×12.3 Å |
| 275 | Propargite | 99.3 | - | 5.57 | 2312-35-8 | C_19_H_26_O_4_S | 350.47 |  | 12.6 Å ×11.2 Å×7.3 Å |
| 276 | Propiconazole | 99.8 | 2.94±0.12 | 3.72 | 60207-90-1 | C_15_H_17_Cl_2_N_3_O_2_ | 342.22 |  | 6.1 Å ×10.5 Å×6.5 Å |
| 277 | Propoxur | 99.9 | 12.28±0.46 | 1.90 | 114-26-1 | C_11_H_15_NO_3_ | 209.24 |  | 3.5 Å ×6.4 Å×8.4 Å |
| 278 | Propyrisulfuron | 99.6 | 11.82±0.70 | 2.22 | 570415-88-2 | C_16_H_18_ClN_7_O_5_S | 455.88 |  | 10.1 Å ×10.7 Å×5.0 Å |
| 279 | Propyzamide | 99.6 | 12.30±0.46 | 3.58 | 23950-58-5 | C_12_H_11_Cl_2_NO | 256.13 |  | 6.7 Å ×7.8 Å×8.3 Å |
| 280 | Proquinazid | 99.9 | - | 3.86 | 189278-12-4 | C_14_H_17_IN_2_O_2_ | 372.20 |  | 7.7 Å ×10.2 Å×7.2 Å |
| 281 | Prosulfocarb | 99.9 | -1.22±0.70 | 4.65 | 52888-80-9 | C_14_H_21_NOS | 251.39 |  | 8.1 Å ×9.8 Å×8.6 Å |
| 282 | Pyraclostrobin | 99.6 | -0.23±0.10 | 5.45 | 175013-18-0 | C_19_H_18_ClN_3_O_4_ | 387.82 |  | 13.6 Å ×9.7 Å×5.9 Å |
| 283 | Pyraflufen-Ethyl | 99.8 | 2.93±0.10 | 4.09 | 129630-17-7 | C_13_H_9_Cl_2_F_3_N_2_O_4_ | 385.12 |  | 10.5 Å ×8.2 Å×5.4 Å |
| 284 | Pyrametostrobin | 98.8 | 2.63±0.10 | 5.90 | 915410-70-7 | C_21_H_23_N_3_O_4_ | 381.42 |  | 6.4 Å ×12.5 Å×11.8 Å |
| 285 | Pyraoxystrobin | 98.9 | - | 4.91 | 862588-11-2 | C_22_H_21_ClN_2_O_4_ | 412.87 |  | 10.3 Å ×13.1 Å×6.1 Å |
| 286 | Pyrazosulfuron-ethyl | 99.5 | 12.06±0.70 | 0.25 | 93697-74-6 | C_14_H_18_N_6_O_7_S | 414.39 |  | 10.4 Å ×12.8 Å×9.9 Å |
| 287 | Pyrethrins I | 93.5 | - | 6.28 | 121-21-1 | C_21_H_28_O_3_ | 328.45 |  | 6.1 Å ×12.2 Å×7.5 Å |
| 288 | Pyribenzoxim | 99.9 | -0.02±0.30 | 5.49 | 168088-61-7 | C_32_H_27_N_5_O_8_ | 609.59 |  | 11.3 Å ×10.3 Å×7.7 Å |
| 289 | Pyridaben | 99.9 | -2.69±0.20 | 5.47 | 96489-71-3 | C_19_H_25_ClN_2_OS | 264.93 |  | 13.5 Å ×11.2 Å×5.7 Å |
| 290 | Pyridalyl | 96.7 | 1.46±0.22 | 7.59 | 179101-81-6 | C_18_H_14_Cl_4_F_3_NO_3_ | 491.12 |  | 14.3 Å ×14.0 Å×3.0 Å |
| 291 | Pyridaphenthion | 95.7 | -2.09±0.40 | 3.66 | 119-12-0 | C_14_H_17_N_2_O_4_PS | 340.33 |  | 7.0 Å ×10.0 Å×7.1 Å |
| 292 | Pyriftalid | 98.0 | -0.47±0.50 | 2.45 | 135186-78-6 | C_15_H_14_N_2_O_4_S | 318.35 |  | 10.1 Å ×9.6 Å×6.7 Å |
| 293 | Pyrimethanil | 99.9 | 3.52 | 3.19 | 53112-28-0 | C_12_H_13_N_3_ | 199.25 |  | 3.7 Å ×8.9 Å×6.4 Å |
| 294 | Pyriproxyfen | 99.6 | 3.2 | 5.55 | 95737-68-1 | C_20_H_19_NO_3_ | 321.38 |  | 10.4 Å ×8.3 Å×7.7 Å |
| 295 | Pyrisoxazole | 98.6 | 4.91±0.12 | 3.38 | 847749-37-5 | C_16_H_17_ClN_2_O | 288.77 |  | 8.5 Å ×7.9 Å×8.3 Å |
| 296 | Quinalphos | 99.9 | -1.05±0.30 | 4.44 | 13593-03-8 | C_12_H_15_N_2_O_3_PS | 298.30 |  | 8.0 Å ×6.2 Å×10.6 Å |
| 297 | Quizalofop-ethyl | 99.9 | -1.39±0.48 | 4.35 | 76578-14-8 | C_19_H_17_ClN_2_O_4_ | 372.80 |  | 9.5 Å ×7.3 Å×11.1 Å |
| 298 | Rotenone | 99.3 | - | 4.31 | 83-79-4 | C_23_H_22_O_6_ | 394.42 |  | 5.3 Å ×9.2 Å×16.1 Å |
| 299 | Saflufenacil | 98.5 | 4.24±0.40 | 1.48 | 372137-35-4 | C_17_H_17_ClF_4_N_4_O_5_S | 500.85 |  | 8.8 Å ×11.0 Å×12.2 Å |
| 300 | Sedaxane | 99.9 | - | 3.66 | 874967-67-6 | C_18_H_19_F_2_N_3_O | 331.36 |  | 7.9 Å ×6.9 Å×7.2 Å |
| 301 | Sethoxydim | 96.8 | 4.38±0.25 | 3.99 | 74051-80-2 | C_17_H_29_NO_3_S | 327.48 |  | 13.4 Å ×11.4 Å×5.5 Å |
| 302 | Silthiofam | 99.9 | - | 5.29 | 175217-20-6 | C_13_H_21_NOSSi | 267.46 |  | 7.5 Å ×8.7 Å×7.4 Å |
| 303 | Simazine | 99.5 | 2.71±0.10 | 2.18 | 122-34-9 | C_7_H_12_ClN_5_ | 201.66 |  | 8.4 Å ×7.8 Å×5.5 Å |
| 304 | Simetryn | 99.9 | 4.05±0.10 | 2.90 | 1014-70-6 | C_8_H_15_N_5_S | 213.30 |  | 8.4 Å ×7.8 Å×6.5 Å |
| 305 | Spinetoram (J) | 95.0 | - | 4.01 | 187166-40-1 | C_42_H_69_NO_10_ | 748.00 |  | 15.5 Å ×17.0 Å×11.3 Å |
| 306 | Spinetoram (L) | 99.0 | 8.62±0.60 | - | 187166-15-0 | C_43_H_69_NO_10_ | 760.01 |  | 20.1 Å ×14.7 Å×7.4 Å |
| 307 | Spinosad A | 97.2 | 8.1 | - | 131929-60-7 | C_41_H_65_NO_10_ | 731.96 |  | 9.8 Å ×13.2 Å×18.8 Å |
| 308 | Spinosad D | 99.5 | 7.8 | 3.85 | 131929-63-0 | C_42_H_67_NO_10_ | 745.98 |  | 17.5 Å ×14.5 Å×10.4 Å |
| 309 | Spirodiclofen | 99.2 | - | 6.21 | 148477-71-8 | C_21_H_24_Cl_2_O_4_ | 411.32 |  | 7.9 Å ×8.3 Å×10.0 Å |
| 310 | Spirotetramat | 95.8 | - | 3.67 | 203313-25-1 | C_21_H_27_NO_5_ | 373.44 |  | 10.2 Å ×9.1 Å×6.6 Å |
| 311 | Spirotetramat enol | 99.2 | 4.50±1.00 | 1.28 | 203312-38-3 | C_18_H_23_NO_3_ | 301.38 |  | 4.5 Å ×11.3 Å×9.9 Å |
| 312 | Spirotetramat ketohydroxy | 99.9 | - | 2.91 | 1172134-11-0 | C_18_H_23_NO_4_ | 317.38 |  | 10.1 Å ×8.3 Å×6.8 Å |
| 313 | Spirotetramat monohydroxy | 99.7 | 13.66±0.60 | 2.27 | 1172134-12-1 | C_18_H_25_NO_3_ | 301.40 |  | 8.0 Å ×9.0 Å×7.1 Å |
| 314 | Spirotetramat-enol-glucoside | 99.9 | 12.01±0.70 | 0.44 | 1172614-86-6 | C_24_H_33_NO_8_ | 463.53 |  | 11.7 Å ×10.1 Å×5.5 Å |
| 315 | Sulfentrazone | 99.9 | 6.56 | 2.65 | 122836-35-5 | C_11_H_10_Cl_2_F_2_N_4_O_3_S | 387.19 |  | 10.1 Å ×9.8 Å×6.2 Å |
| 316 | Sulfotep | 95.0 | - | 3.98 | 3689-24-5 | C_8_H_20_O_5_P_2_S_2_ | 322.31 |  | 8.9 Å ×8.4 Å×5.7 Å |
| 317 | Sulfoxaflor | 99.5 | - | 3.14 | 946578-00-3 | C_10_H_10_F_3_N_3_OS | 277.27 |  | 6.3 Å ×5.1 Å×9.2 Å |
| 318 | Tebuconazole | 98.7 | - | 3.89 | 80443-41-0 | C_16_H_22_ClN_3_O | 307.82 |  | 7.4 Å ×10.0 Å×6.7 Å |
| 319 | Tebufenozide | 98.2 | 10.89±0.46 | 4.25 | 112410-23-8 | C_22_H_28_N_2_O_2_ | 352.48 |  | 7.7 Å ×11.9 Å×9.2 Å |
| 320 | Tebuthiuron | 99.9 | 13.36±0.46 | 1.79 | 34014-18-1 | C_9_H_16_N_4_OS | 228.31 |  | 6.2 Å ×6.6 Å×8.3 Å |
| 321 | Teflubenzuron | 98.6 | 8.16±0.46 | 4.64 | 83121-18-0 | C_14_H_6_Cl_2_F_4_N_2_O_2_ | 381.11 |  | 7.9 Å ×7.7 Å×5.2 Å |
| 322 | Terbufos sulfone | 98.8 | - | 2.46 | 56070-16-7 | C_9_H_21_O_4_PS_3_ | 320.43 |  | 9.7 Å ×5.2 Å×8.0 Å |
| 323 | Terbufos sulfoxide | 98.8 | - | 2.35 | 10548-10-4 | C_9_H_21_O_3_PS_3_ | 304.43 |  | 7.0 Å ×5.6 Å×11.0 Å |
| 324 | Terbuthylazine | 99.9 | 2.69±0.10 | 3.27 | 5915-41-3 | C_9_H_16_ClN_5_ | 229.71 |  | 6.4 Å ×5.8 Å×7.1 Å |
| 325 | Tetraconazole | 99.0 | 2.68±0.10 | 3.56 | 112281-77-3 | C_13_H_11_Cl_2_F_4_N_3_O | 372.14 |  | 6.8 Å ×8.4 Å×8.6 Å |
| 326 | Thiabendazole | 99.9 | 4.7 | 2.00 | 148-79-8 | C_10_H_7_N_3_S | 201.25 |  | 5.2 Å ×3.8 Å×9.7 Å |
| 327 | Thiacloprid | 99.5 | 0.01±0.10 | 2.33 | 111988-49-9 | C_10_H_9_ClN_4_S | 252.72 |  | 7.0 Å ×5.7 Å×6.2 Å |
| 328 | Thiamethoxam | 99.6 | 0.99±0.10 | 0.8 | 153719-23-4 | C_8_H_10_ClN_5_O_3_S | 291.71 |  | 8.2 Å ×5.8 Å×6.9 Å |
| 329 | Thidiazuron | 99.9 | 12.06±0.70 | 2.1 | 51707-55-2 | C_9_H_8_N_4_OS | 220.25 |  | 6.5 Å ×5.3 Å×7.1 Å |
| 330 | Thifensulfuron methyl | 98.8 | 4.0 | 1.27 | 79277-27-3 | C_12_H_13_N_5_O_6_S_2_ | 387.39 |  | 9.9 Å ×7.9 Å×7.2 Å |
| 331 | Thifluzamide | 99.9 | 8.24±0.70 | 4.91 | 130000-40-7 | C_13_H_6_Br_2_F_6_N_2_O_2_S | 528.06 |  | 8.3 Å ×8.9 Å×7.2 Å |
| 332 | Thiophanate-methyl | 99.9 | 7.28 | 1.10 | 23564-05-8 | C_12_H_14_N_4_O_4_S_2_ | 342.39 |  | 7.0 Å ×6.7 Å×7.6 Å |
| 333 | Tolclofos-methyl | 97.0 | - | 4.56 | 57018-04-9 | C_9_H_11_Cl_2_O_3_PS | 301.13 |  | 8.4 Å ×8.6 Å×2.3 Å |
| 334 | Tolfenpyrad | 98.7 | 13.11±0.46 | 6.45 | 129558-76-5 | C_21_H_22_ClN_3_O_2_ | 383.87 |  | 9.3 Å ×13.5 Å×7.6 Å |
| 335 | Tralkoxydim | 99.9 | 4.20±0.25 | 4.87 | 87820-88-0 | C_20_H_27_NO_3_ | 329.43 |  | 13.2 Å ×8.6 Å×6.0 Å |
| 336 | Triadimefon | 98.7 | 1.41±0.11 | 2.77 | 43121-43-3 | C_14_H_16_ClN_3_O_2_ | 293.75 |  | 6.9 Å ×5.8 Å×9.9 Å |
| 337 | Triadimenol | 99.9 | 13.29±0.20 | 3.08 | 55219-65-3 | C_14_H_18_ClN_3_O_2_ | 295.77 |  | 8.0 Å ×10.0 Å×4.9 Å |
| 338 | Triallate | 99.8 | -1.48±0.70 | 4.60 | 2303-17-5 | C_10_H_16_Cl_3_NOS | 304.65 |  | 4.0 Å ×11.0 Å×7.6 Å |
| 339 | Triasulfuron |  | 4.64 | 2.44 | 82097-50-5 | C_14_H_16_ClN_5_O_5_S | 401.83 |  | 8.6 Å ×13.3 Å×4.6 Å |
| 340 | Triazophos | 98.5 | -0.15±0.50 | 3.37 | 24017-47-8 | C_12_H_16_N_3_O_3_PS | 313.31 |  | 11.0 Å ×8.3 Å×7.8 Å |
| 341 | Trichlorfon | 98.7 | 6.0 | 0.42 | 52-68-6 | C_4_H_8_Cl_3_O_4_P | 257.44 |  | 5.3 Å ×6.5 Å×4.2 Å |
| 342 | Trifloxystrobin | 99.9 | - | 6.62 | 141517-21-7 | C_20_H_19_F_3_N_2_O_4_ | 408.37 |  | 10.5 Å ×10.9 Å×8.7 Å |
| 343 | Triflumizole | 99.9 | 3.7 | 1.50 | 99387-89-0 | C_15_H_15_ClF_3_N_3_O | 345.75 |  | 3.0 Å ×9.0 Å×10.0 Å |
| 344 | Triflumuron | 99.9 | 9.79±0.46 | 4.91 | 64628-44-0 | C_15_H_10_ClF_3_N_2_O_3_ | 358.70 |  | 15.1 Å ×7.2 Å×3.9 Å |
| 345 | Triflusulfuron-methyl | 98.2 | 4.4 | 3.94 | 126535-15-7 | C_17_H_19_F_3_N_6_O_6_S | 492.43 |  | 10.7 Å ×8.3 Å×7.7 Å |
| 346 | Triticonazole | 98.3 | 13.23±0.40 | 4.11 | 131983-72-7 | C_17_H_20_ClN_3_O | 317.81 |  | 6.2 Å ×9.8 Å×4.9 Å |
| 347 | Tritosulfuron | 99.9 | 4.69 | 4.480 | 142469-14-5 | C_13_H_9_F_6_N_5_O_4_S | 445.3 |  | 11.7 Å ×6.3 Å×7.7 Å |
| 348 | Vamidothion | 99.3 | 15.22±0.46 | 1.28 | 2275-23-2 | C_8_H_18_NO_4_PS_2_ | 287.34 |  | 8.0 Å ×10.8 Å×6.3 Å |
| 349 | Zoxamide | 98.7 | 11.20±0.46 | 4.35 | 156052-68-5 | C_14_H_16_Cl_3_NO_2_ | 336.64 |  | 10.1 Å ×8.1 Å×8.4 Å |
| 350 | Benazolin-ethyl | 99.3 | -2.57±0.20 | 2.86 | 25059-80-7 | C_11_H_10_ClNO_3_S | 271.72 |  | 6.5 Å ×5.6 Å×8.4 Å |
| 351 | Cyazofamid metabolite CCIM | 99.4 | - | 3.11 | 120118-14-1 | C_11_H_8_ClN_3_ | 217.65 |  | 8.3 Å ×8.2 Å×2.8 Å |
| 352 | Fensulfothion sulfone | 99.8 | - | 2.48 | 14255-72-2 | C_11_H_17_O_5_PS_2_ | 324.35 |  | 7.4 Å ×5.7 Å×11.1 Å |
| 353 | Mesosulfuron methyl | 98.3 | 4.35 | 0.80 | 208465-21-8 | C_17_H_21_N_5_O_9_S_2_ | 503.51 |  | 11.4 Å ×10.3 Å×6.2 Å |
| 354 | Pirimicarb-desmethyl-formamido | 99.9 | 3.52±0.10 | 0.91 | 27218-04-8 | C_11_H_16_N_4_O_3_ | 252.27 |  | 9.0 Å ×7.9 Å×4.9 Å |
| 355 | Prochloraz metabolite BTS44596 | 99.0 | 10.27±0.46 | 3.87 | 139542-32-8 | C_13_H_15_Cl_3_N_2_O_3_ | 353.63 |  | 7.3 Å ×10.4 Å×7.0 Å |
| 356 | Propisochlor | 98.5 | 1.30±0.50 | 3.5 | 86763-47-5 | C_15_H_22_ClNO_2_ | 283.30 |  | 7.8 Å ×7.7 Å×6.7 Å |
| 357 | Pyrethrins II | 97.6 | - | 5.33 | 121-29-9 | C_22_H_28_O_5_ | 372.45 |  | 8.6 Å ×9.7 Å×6.7 Å |
| 358 | Pyrimorph | 99.9 | - | 3.44 | 868390-90-3 | C_22_H_25_ClN_2_O_2_ | 384.90 |  | 9.3 Å ×13.3 Å×7.7 Å |
| 359 | Spiromesifen | 99.2 | - | 6.08 | 283594-90-1 | C_23_H_30_O_4_ | 370.49 |  | 7.6 Å ×11.9 Å×7.7 Å |
| 360 | Terbufos | 99.0 | - | 3.8 | 13071-79-9 | C_9_H_21_O_2_PS_3_ | 288.431 |  | 5.9 Å ×3.9 Å×5.7 Å |
| 361 | triflumizole Metabolite FM-6-1 | 99.9 | - | 1.57 | 131549-75-2 | C_12_H_14_ClF_3_N_2_O | 294.70 |  | 9.8 Å ×5.8 Å×6.4 Å |
| 362 | Uniconazole | 99.8 | 13.07±0.20 | 3.28 | 83657-22-1 | C_15_H_18_ClN_3_O | 291.78 |  | 7.7 Å ×6.7 Å×9.0 Å |
| 363 | Xylazine | 99.0 | 7.67±0.20 | 4.52 | 7361-61-7 | C_12_H_16_N_2_S | 220.33 |  | 5.5 Å ×7.9 Å×5.7 Å |
| 364 | Pindolol | 99.0 | 9.26 | 1.48 | 13523-86-9 | C_14_H_20_N_2_O_2_ | 248.32 |  | 6.8 Å ×10.2 Å×8.1 Å |
| 365 | Alprenolol | 98.0 | 9.63 | 2.82 | 13655-52-2 | C_15_H_23_NO_2_ | 249.35 |  | 6.2 Å ×7.6 Å×8.9 Å |
| 366 | Propranolol | 95.0 | 9.53±0.01 | 2.60 | 5051-22-9 | C_16_H_21_NO_2_ | 259.34 |  | 7.5 Å ×7.7 Å×7.3 Å |
| 367 | Oxprenolol | 95.0 | - | 1.83 | 6452-71-7 | C_15_H_23_NO_3_ | 265.35 |  | 6.5 Å ×8.1 Å×11.0 Å |
| 368 | Acebutolol | 95.0 | 9.40 | 1.91 | 37517-30-9 | C_18_H_28_N_2_O_4_ | 336.43 |  | 10.3 Å ×9.9 Å×7.4 Å |
| 369 | Metoprolol | 98.0 | - | 1.69 | 37350-58-6 | C_15_H_25_NO_3_ | 267.36 |  | 5.5 Å ×8.1 Å×15.0 Å |
| 370 | Levobunolol | 98.0 | - | 2.48 | 47141-42-4 | C_17_H_25_NO_3_ | 291.39 |  | 10.6 Å ×10.0 Å×6.6 Å |
| 371 | Carazolol | 98.0 | - | 2.66 | 57775-29-8 | C_18_H_22_N_2_O_2_ | 298.38 |  | 5.7 Å ×9.0 Å×9.6 Å |
| 372 | Timolol | 95.0 | 9.20 | 1.75 | 26839-75-8 | C_13_H_24_N_4_O_3_S | 316.42 |  | 6.5 Å ×8.3 Å×5.8 Å |
| 373 | Betaxolol | 98.0 | 9.21 | 2.98 | 63659-18-7 | C_18_H_29_NO_3_ | 307.43 |  | 9.0 Å ×14.8 Å×9.2 Å |
| 374 | Droperidol | 99.0 | 7.64 | 3.46 | 548-73-2 | C_22_H_22_FN_3_O_2_ | 379.43 |  | 6.4 Å ×14.9 Å×6.3 Å |
| 375 | Carvedilol | 98.0 | 13.90±0.20 | 3.05 | 72956-09-3 | C_24_H_26_N_2_O_4_ | 406.47 |  | 11.2 Å ×13.4 Å×10.9 Å |
| 376 | Nebivolol | 98.0 | 14.29±0.20 | 3.71 | 99200-09-6 | C_22_H_25_F_2_NO_4_ | 405.43 |  | 6.8 Å ×16.7 Å×5.7 Å |
| 377 | Acepromazine | 99.0 | 9.41±0.28 | 4.24 | 61-00-7 | C_19_H_22_N_2_OS | 326.46 |  | 11.6 Å ×9.5 Å×4.3 Å |
| 378 | Chlorpromazine | 98.0 | 9.30 | 4.81 | 50-53-3 | C_17_H_19_ClN_2_S | 318.86 |  | 9.9 Å ×9.5 Å×4.5 Å |
| 379 | Nadolol | 99.0 | 9.67 | 1.17 | 42200-33-9 | C_17_H_27_NO_4_ | 309.4 |  | 9.1 Å ×10.6 Å×7.0 Å |
| 380 | Estazolam | 98.0 | - | 3.32 | 29975-16-4 | C_16_H_11_ClN_4_ | 294.74 |  | 5.0 Å ×8.6 Å×9.1 Å |
| 381 | Penbutolol | 98.0 | - | 4.20 | 36507-48-9 | C_18_H_29_NO_2_ | 291.43 |  | 6.7 Å ×8.4 Å×8.2 Å |
| 382 | Demoxepam | 98.0 | 4.5 | -2.12 | 963-39-3 | C_15_H_11_ClN_2_O_2_ | 286.71 |  | 4.8 Å ×8.2 Å×9.2 Å |
| 383 | Diazapam | 99.5 | - | 2.70 | 439-14-5 | C_16_H_13_ClN_2_O | 284.74 |  | 4.2 Å ×8.1 Å×9.7 Å |
| 384 | Nitrazepam | 95.0 | - | 2.45 | 146-22-5 | C_15_H_11_N_3_O_3_ | 281.27 |  | 4.1 Å ×8.6 Å×9.1 Å |
| 385 | Acetylkitasamycin | 98.0 | - | 2.45 | 71251-30-4 | C_43_H_69_NO_16_ | 856.02 |  | 14.4 Å ×12.0 Å×10.3 Å |
| 386 | Narasin | 98.0 | - | - | 58331-17-2 | C_43_H_71_O_11_Na | 789.04 |  | 18.7 Å ×9.8 Å×10.8 Å |
| 387 | Salinomycin | 98.0 | 6.4 | 8.53 | 53003-10-4 | C_42_H_70_O_11_ | 751.00 |  | 9.9 Å ×9.2 Å×18.7 Å |
| 388 | Monensin | 95.0 | 6.6 | 5.43 | 17090-79-8 | C_36_H_62_O_11_ | 670.87 |  | 13.4 Å ×9.7 Å×16.6 Å |
| 389 | luftong | - | - | - | - | - | - | - | - |
| 390 | Sulfanitran | 98.0 | 7.42±0.10 | 2.26 | 122-16-7 | C_14_H_13_N_3_O_5_S | 335.34 |  | 6.4 Å ×8.4 Å×9.8 Å |
| 391 | Guanabenz | 98.0 | 8.1 | 1.70 | 5051-62-7 | C_8_H_8_Cl_2_N_4_ | 231.08 |  | 5.9 Å ×8.7 Å×2.8 Å |
| 392 | Trimethoprim | 98.0 | 6.6 | 0.73 | 738-70-5 | C_14_H_18_N_4_O_3_ | 290.32 |  | 5.2 Å ×8.3 Å×10.6 Å |
| 393 | Diaveridine | 98.0 | 7.11±0.10 | 0.86 | 5355-16-8 | C_13_H_16_N_4_O_2_ | 260.29 |  | 10.9 Å ×8.3 Å×4.1 Å |
| 394 | Ethopabate | 98.0 | 14.12±0.70 | 1.90 | 59-06-3 | C_12_H_15_NO_4_ | 237.25 |  | 5.8 Å ×6.0 Å×9.3 Å |
| 395 | Sulfachlorpyridazine | 98.0 | 6.10 | 0.31 | 80-32-0 | C_10_H_9_ClN_4_O_2_S | 284.72 |  | 4.6 Å ×9.0 Å×10.0 Å |
| 396 | Sulfamonomethoxine | 98.0 | 5.94 | 0.20 | 1220-83-3 | C_11_H_12_N_4_O_3_S | 280.30 |  | 3.2 Å ×6.5 Å×13.0 Å |
| 397 | Sulfisomidine | 99.0 | 7.25 | 0.76 | 515-64-0 | C_12_H_14_N_4_O_2_S | 278.33 |  | 7.1 Å ×8.8 Å×7.0 Å |
| 398 | Sulfabenzamine | 98.0 | 10.16±0.10 | -0.55 | 138-39-6 | C_7_H_10_N_2_O_2_S | 186.23 |  | 3.3 Å ×7.9 Å×6.7 Å |
| 399 | Sulfamethizole | 99.0 | 5.45 | 0.41 | 144-82-1 | C_9_H_10_N_4_O_2_S_2_ | 270.33 |  | 3.2 Å ×4.7 Å×12.8 Å |
| 400 | Sulfamoxole | 99.0 | 7.40 | 1.03 | 729-99-7 | C_11_H_13_N_3_O_3_S | 267.30 |  | 5.1 Å ×6.7 Å×10.2 Å |
| 401 | Sulfisoxazole | 98.0 | 5.00 | 1.03 | 127-69-5 | C_11_H_13_N_3_O_3_S | 267.30 |  | 5.2 Å ×9.0 Å×6.8 Å |
| 402 | Sulfamerazine | 98.0 | 2.29 | 0.21 | 127-79-7 | C_11_H_12_N_4_O_2_S | 264.30 |  | 5.2 Å ×10.0 Å×8.8 Å |
| 403 | Sulfathiazole | 98.0 | 7.2 | 0.72 | 72-14-0 | C_9_H_9_N_3_O_2_S_2_ | 255.32 |  | 3.2 Å ×11.2 Å×7.2 Å |
| 404 | Sulfamethoxazole | 98.0 | 5.60±0.05 | 0.48 | 723-46-6 | C_10_H_11_N_3_O_3_S | 253.28 |  | 5.1 Å ×6.7 Å×8.5 Å |
| 405 | Sulfapyridine | 98.0 | 8.48 | 0.53 | 144-83-2 | C_11_H_11_N_3_O_2_S | 249.29 |  | 5.1 Å ×9.5 Å×8.9 Å |
| 406 | Sulfaguanidine | 98.0 | 2.37 | -1.07 | 57-67-0 | C_7_H_10_N_4_O_2_S | 214.24 |  | 4.5 Å ×6.9 Å×8.5 Å |
| 407 | Sulfaquinoxaline | 98.0 | 5.65±0.10 | 0.84 | 59-40-5 | C_14_H_12_N_4_O_2_S | 300.34 |  | 6.9 Å ×8.6 Å×8.6 Å |
| 408 | Sulfadimethoxine | 98.0 | 5.94 | 1.17 | 122-11-2 | C_12_H_14_N_4_O_4_S | 310.33 |  | 3.2 Å ×6.5 Å×13.0 Å |
| 409 | Sulfadoxine | 98.0 | 6.16±0.50 | -0.24 | 2447-57-6 | C_12_H_14_N_4_O_4_S | 310.33 |  | 6.1 Å ×10.2 Å×9.3 Å |
| 410 | Sulfadimidine | 98.0 | - | 0.76 | 57-68-1 | C_12_H_14_N_4_O_2_S | 278.33 |  | 3.4 Å ×6.7 Å×12.2 Å |
| 411 | Ronidazole | 97.0 | 1.2 | -0.37 | 7681-76-7 | C_6_H_8_N_4_O_4_ | 200.15 |  | 2.1 Å ×8.0 Å×7.3 Å |
| 412 | Metronidazole-hydroxy | 98.0 | 13.28±0.10 | -1.06 | 4812-40-2 | C_6_H_9_N_3_O_4_ | 187.15 |  | 2.3 Å ×6.2 Å×6.6 Å |
| 413 | Metronidazole | 98.0 | 2.62 | -0.003 | 443-48-1 | C_6_H_9_N_3_O_3_ | 171.15 |  | 2.3 Å ×6.2 Å×6.6 Å |
| 414 | Ipronidazole | 98.0 | 2.55±0.25 | - | 14885-29-1 | C_7_H_11_N_3_O_2_ | 169.18 |  | 1.4 Å ×5.3 Å×6.9 Å |
| 415 | 5-nitrobenzimidazole | 98.0 | 10.95±0.10 | 1.05 | 94-52-0 | C_7_H_5_N_3_O_2_ | 163.13 |  | 3.4 Å ×4.3 Å×6.9 Å |
| 416 | 5-chloro-1-methyl-4-nitroimidazole | 98.0 | -1.37±0.61 | 1.07 | 4897-25-0 | C_4_H_4_ClN_3_O_2_ | 161.55 |  | 1.8 Å ×6.2 Å×4.6 Å |
| 417 | Dimetridazole-2-hydroxy | 98.0 | 13.31±0.10 | -0.49 | \| 936-05-0 | C_5_H_7_N_3_O_3_ | 157.13 |  | 2.1 Å ×5.7 Å×5.9 Å |
| 418 | Dimetridazole | 98.0 | 2.81±0.25 | 0.97 | 551-92-8 | C_5_H_7_N_3_O_2_ | 141.13 |  | 2.1 Å ×5.2 Å×5.9 Å |
| 419 | 4-nitroimidazole | 98.0 | 8.31±0.10 | -0.12 | 3034-38-6 | C_3_H_3_N_3_O_2_ | 113.07 |  | 0.8 Å ×5.3 Å×4.0 Å |
| 420 | Enoxacin | 98.0 | 6.04±0.70 | -0.21 | 74011-58-8 | C_15_H_17_FN_4_O_3_ | 320.32 |  | 11.5 Å ×7.5 Å×4.1 Å |
| 421 | Kitasamycin | 98.0 | - | 3.00 | 16846-34-7 | C_40_H_67_NO_14_ | 785.96 |  | 9.0 Å ×9.6 Å×15.9 Å |
| 422 | Flumequine | 98.0 | 6.42 | 1.6 | 42835-25-6 | C_14_H_12_FNO_3_ | 261.25 |  | 5.8 Å ×6.6 Å×6.1 Å |
| 423 | Roxithromycin | 98.0 | 9.27±0.01 | 2.75 | 80214-83-1 | C_41_H_76_N_2_O_15_ | 837.05 |  | 10.7 Å ×16.8 Å×11.5 Å |
| 424 | Virginiamycin S1 | 98.0 | - | 0.32 | 23152-29-6 | C_43_H_49_N_7_O_10_ | 823.89 |  | 7.2 Å ×15.4 Å×14.2 Å |
| 425 | Rifampicin | 98.0 | 1.7/7.9 | 2.7 | 13292-46-1 | C_43_H_58_N_4_O_12_ | 822.94 |  | 5.1 Å ×12.3 Å×16.8 Å |
| 426 | Midecamycin | 98.0 | 6.9 | 2.74 | 35457-80-8 | [C_41_H_67_NO_15_](https://pubchem.ncbi.nlm.nih.gov/) | 813.97 |  | 10.2 Å ×18.6 Å×10.7 Å |
| 427 | Desmycosin | 95.0 | 8.36 | 1.23 | 11032-98-7 | C_39_H_65_NO_14_ | 771.93 |  | 11.1 Å ×11.7 Å×12.1 Å |
| 428 | Oleandomycin | 98.0 | 8.84 | 1.83 | 3922-90-5 | C_35_H_61_NO_12_ | 687.86 |  | 9.9 Å ×9.3 Å×11.7 Å |
| 429 | Virginiamycin M1 | 98.0 | 13.18±0.70 | 0.12 | 21411-53-0 | C_28_H_35_N_3_O_7_ | 525.59 |  | 15.2 Å ×10.1 Å×5.5 Å |
| 430 | Lincomycin | 98.0 | 7.60 | - | 154-21-2 | C_18_H_34_N_2_O_6_S | 406.54 |  | 11.5 Å ×8.9 Å×6.7 Å |
| 431 | Tylosin tartrate | 99.0 | - | 1.05 | 1405-54-5 | C_46_H_77_NO_17_ | 916.10 |  | 12.0 Å ×10.0 Å×13.8 Å |
| 432 | Acetanilide | 99.0 | 0.5 | 1.10 | 103-84-4 | C_8_H_9_NO | 135.16 |  | 6.7 Å ×4.7 Å×5.0 Å |
| 433 | Benzocaine | 99.0 | 2.5 | 1.80 | 94-09-7 | C_9_H_11_NO_2_ | 165.19 |  | 7.7 Å ×5.5 Å×5.9 Å |
| 434 | Detomidine | 98.0 | 14.44±0.10 | 3.41 | 76631-46-4 | C_12_H_14_N_2_ | 186.25 |  | 8.5 Å ×5.6 Å×7.0 Å |
| 435 | Levamisole | 98.0 | 10.00±0.40 | 2.87 | 14769-73-4 | C_11_H_12_N_2_S | 204.29 |  | 6.4 Å ×7.9 Å×4.8 Å |
| 436 | Clonidine | 98.0 | 8.10±0.50 | 1.45 | 4205-90-7 | C_9_H_9_Cl_2_N_3_ | 230.09 |  | 5.6 Å ×8.3 Å×3.9 Å |
| 437 | Lidocaine | 99.0 | 7.88 | 1.66 | 137-58-6 | C_14_H_22_N_2_O | 234.34 |  | 6.0 Å ×7.4 Å×7.1 Å |
| 438 | flurbiprofen | 98.0 | 3.80 | 3.81 | 5104-49-4 | C_15_H_13_FO_2_ | 244.27 |  | 10.1 Å ×5.0 Å×7.3 Å |
| 439 | Fenbufen | 98.0 | 4.3 | 3.18 | 36330-85-5 | C_16_H_14_O_3_ | 254.29 |  | 11.1 Å ×6.7 Å×8.7 Å |
| 440 | Diphenhydramine | 99.0 | 9.1 | 3.11 | 58-73-1 | C_17_H_21_NO | 255.35 |  | 9.4 Å ×7.7 Å×7.0 Å |
| 441 | Clenproperol | 95.0 | 13.27±0.20 | 1.55 | 38339-11-6 | [C_11_H_16_C_l2_N_2_O](https://pubchem.ncbi.nlm.nih.gov/) | 263.16 |  | 7.4 Å ×7.0 Å×7.5 Å |
| 442 | Antazoline | 98.0 | 6.65 | 3.38 | 91-75-8 | C_17_H_19_N_3_ | 265.35 |  | 8.0 Å ×8.0 Å×6.5 Å |
| 443 | Chlormezanone | 98.0 | -2.37±0.40 | -0.26 | 80-77-3 | C_11_H_12_ClNO_3_S | 273.74 |  | 5.6 Å ×5.7 Å×9.3 Å |
| 444 | Clotrimazole | 99.0 | 4.7 | 6.26 | 23593-75-1 | C_22_H_17_ClN_2_ | 344.84 |  | 8.7 Å ×8.5 Å×6.8 Å |
| 445 | Doxepin | 98.0 | 9.40±0.28 | 3.99 | 1668-19-5 | C_19_H_21_NO | 279.38 |  | 7.0 Å ×10.2 Å×7.7 Å |
| 446 | Cyproheptadine | 98.0 | 8.87 | 5.17 | 129-03-3 | C_21_H_21_N | 287.40 |  | 9.6 Å ×9.5 Å×7.0 Å |
| 447 | Clencyclohexerol | 99.5 | 13.26±0.20 | 3.8 | 157877-79-7 | C_14_H_20_Cl_2_N_2_O_2_ | 319.23 |  | 5.2Å ×10.1 Å×7.5 Å |
| 448 | Clenpenterol | 95.0 | 13.28±0.20 | 2.49 | 38339-21-8 | [C_13_H_20_Cl_2_N_2_O](https://pubchem.ncbi.nlm.nih.gov/) | 291.22 |  | 9.5 Å ×5.7 Å×5.8 Å |
| 449 | Anastrozole | 99.5 | 2.62±0.10 | 2.37 | 120511-73-1 | C_17_H_19_N_5_ | 293.37 |  | 7.5 Å ×10.1 Å×6.8 Å |
| 450 | Diclofenac | 98.0 | 4.00 | 4.01 | 15307-86-5 | C_14_H_11_Cl_2_NO_2_ | 296.15 |  | 7.1 Å ×5.6 Å×6.2 Å |
| 451 | Isoxsuprine | 98.0 | 8.0 | - | 579-56-6 | C_18_H_24_ClNO_3_ | 337.84 |  | 8.0 Å ×11.0 Å×8.7 Å |
| 452 | Fluconazole | 98.0 | 1.76±0.1 | 0.25 | 86386-73-4 | C_13_H_12_F_2_N_6_O | 306.27 |  | 8.3 Å ×6.8 Å×7.6 Å |
| 453 | Ketotifen | 98.0 | 8.84±0.20 | 3.85 | 34580-13-7 | C_19_H_19_NOS | 309.43 |  | 7.7 Å ×10.4 Å×6.9 Å |
| 454 | Bifonazole | 99.0 | 6.55±0.22 | 5.71 | 60628-96-8 | C_22_H_18_N_2_ | 310.40 |  | 11.5 Å ×7.3 Å×7.5 Å |
| 455 | Kresoxim-Methyl | 97.0 | - | 3.44 | 143390-89-0 | C_18_H_19_NO_4_ | 313.35 |  | 5.3 Å ×13.1 Å×7.3 Å |
| 456 | Clomipramine | 97.0 | 9.38 | 5.65 | 303-49-1 | C_19_H_23_ClN_2_ | 314.86 |  | 5.1 Å ×9.7 Å×10.5 Å |
| 457 | Chlorprothixene | 98.0 | 8.4 | 5.14 | 113-59-7 | C_18_H_18_ClNS | 315.86 |  | 9.4 Å ×10.0 Å×5.0 Å |
| 458 | Gliclazide | 98.0 | 6.07±0.10 | 2.12 | 21187-98-4 | C_15_H_21_N_3_O_3_S | 323.41 |  | 5.3 Å ×11.0 Å×7.3 Å |
| 459 | Citalopram | 98.0 | 9.38 | 3.74 | 59729-33-8 | C_20_H_21_FN_2_O | 324.39 |  | 10.2 Å ×8.4 Å×8.8 Å |
| 460 | Danazol | 98.0 | 13.10±0.60 | 4.21 | 17230-88-5 | C_22_H_27_NO_2_ | 337.46 |  | 11.0 Å ×7.5 Å×7.8 Å |
| 461 | Griseofulvin | 97.0 | - | 1.92 | 126-07-8 | C_17_H_17_ClO_6_ | 352.77 |  | 7.7 Å ×9.9 Å×7.7 Å |
| 462 | Bisacodyl | 98.0 | 4.69±0.10 | 3.453 | 603-50-9 | C_22_H_19_NO_4_ | 361.39 |  | 10.2 Å ×6.9 Å×8.9 Å |
| 463 | Hydrocortisone | 99.0 | - | 1.62 | 50-23-7 | C_21_H_30_O_5_ | 362.47 |  | 11.3 Å ×8.8 Å×6.1 Å |
| 464 | Bumetanide | 98.0 | 7.7 | 2.57 | 28395-03-1 | C_17_H_20_N_2_O_5_S | 364.42 |  | 10.6 Å ×8.2 Å×4.9 Å |
| 465 | Indapamide | 99.0 | 8.8 ± 0.2 | 2.67 | 26807-65-8 | C_16_H_16_ClN_3_O_3_S | 365.83 |  | 9.2 Å ×8.0 Å×7.8 Å |
| 466 | Bromhexine | 98.0 | 8.34±0.20 | 4.88 | 3572-43-8 | C_14_H_20_Br_2_N_2_ | 376.13 |  | 6.1 Å ×7.3 Å×6.5 Å |
| 467 | Doxapram | 98.0 | 7.30±0.10 | 3.42 | 309-29-5 | C_24_H_30_N_2_O_2_ | 378.51 |  | 8.3 Å ×9.9 Å×8.0 Å |
| 468 | Econazol | 98.0 | 6.68±0.12 | 5.61 | 27220-47-9 | C_18_H_15_Cl_3_N_2_O | 381.68 |  | 11.7 Å ×6.1 Å×9.7 Å |
| 469 | Betamethasone | 99.0 | 12.13±0.70 | 1.72 | 378-44-9 | C_22_H_29_FO_5_ | 392.47 |  | 13.3 Å ×8.1 Å×6.7 Å |
| 470 | Benzthiazide | 98.0 | 9.57±0.20 | 2.20 | 91-33-8 | C_15_H_14_ClN_3_O_4_S_3_ | 431.94 |  | 14.1 Å ×8.3 Å×8.1 Å |
| 471 | Glipizide | 98.0 | 5.9 | 3.35 | 29094-61-9 | C_21_H_27_N_5_O_4_S | 445.54 |  | 11.0 Å ×15.7 Å×5.1 Å |
| 472 | Glimepiride | 95.0 | 5.10±0.10 | 4.70 | 93479-97-1 | C_24_H_34_N_4_O_5_S | 490.62 |  | 12.6 Å ×12.2 Å×8.3 Å |
| 473 | Glibenclamide | 98.0 | 5.3 | 4.79 | 10238-21-8 | C_23_H_28_ClN_3_O_5_S | 494.00 |  | 16.3 Å ×17.8 Å×5.4 Å |
| 474 | Dipyridamole | 98.0 | 6.4 | 2.74 | 58-32-2 | C_24_H_40_N_8_O_4_ | 505.63 |  | 11.7 Å ×14.9 Å×4.9 Å |
| 475 | Thiabendazole | 98.0 | 12.39±0.20 | -9.88 | 56180-94-0 | C_25_H_43_NO_18_ | 654.61 |  | 16.5 Å ×10.5 Å×9.3 Å |
| 476 | Levamisole Hydrochloride | 99.0 | - | - | 16595-80-5 | C_11_H_13_ClN_2_S | 240.75 |  | 9.2 Å ×3.5 Å×6.9 Å |
| 477 | 5-hydroxythiabendazole | 98.0 | 8.76±0.40 | 1.64 | 948-71-0 | C_10_H_7_N_3_OS | 217.25 |  |  |
| 478 | 4-acetylaminoantipyrine | 98.0 | 12.84±0.20 | -0.13 | 83-15-8 | C_13_H_15_N_3_O_2_ | 245.28 |  | 8.6 Å ×9.3 Å×3.6 Å |
| 479 | Albendazole-2-Aminosulfone | 98.0 | 9.90±0.10 | - | 80983-34-2 | C_10_H_13_N_3_O_2_ S | 239.29 |  |  |
| 480 | sudan 1 | 98.0 | 13.50±0.40 | 5.51 | 842-07-9 | C_16_H_12_N_2_O | 248.28 |  | 7.8 Å ×6.0 Å×9.3 Å |
| 481 | Oxibendazole | 98.0 | 10.60±0.10 | 1.98 | 20559-55-1 | C_12_H_15_N_3_O_3_ | 249.27 |  |  |
| 482 | Albendazole | 98.0 | 10.72±0.10 | 2.7 | 54965-21-8 | C_12_H_15_N_3_O_2_S | 265.33 |  |  |
| 483 | Disperse yellow 3 | 98.0 | 8.74±0.43 | 3.99 | 2832-40-8 | C_15_H_15_N_3_O_2_ | 269.30 |  | 11.7 Å ×10.2 Å×4.6 Å |
| 484 | Tolbutamide | 98.0 | 5.32 | 2.41 | 64-77-7 | C_12_H_18_N_2_O_3_S | 270.35 |  | 6.2 Å ×13.3 Å×4.6 Å |
| 485 | Sudan 2 | 98.0 | 13.52±0.50 | 6.60 | 3118-97-6 | C_18_H_16_N_2_O | 276.33 |  | 8.8 Å ×6.9 Å×10.5 Å |
| 486 | Albendazole sulfoxide | 98.0 | 3.28±0.01 | 0.97 | 54029-12-8 | C_12_H_15_N_3_O_3_S | 281.33 |  | 9.4 Å ×11.0 Å×8.6 Å |
| 487 | N-acetyl dapsone | 98.0 | - | 0.80 | 565-20-8 | C_14_H_14_N_2_O_3_S | 290.34 |  | 6.7 Å ×7.0 Å×11.6 Å |
| 488 | Fenbendazole sulfone | 95.0 | 10.14±0.10 | 2.17 | 54029-20-8 | C_15_H_13_N_3_O_4_S | 331.35 |  | 8.4 Å ×12.4 Å×7.4 Å |
| 489 | 5-Hydroxymebendazole | 98.0 | 11.36±0.10 | 2.5 | 60254-95-7 | C_16_H_15_N_3_O_3_ | 297.31 |  | 8.5 Å ×10.4 Å×8.5 Å |
| 490 | Cambendazole | 98.0 | 9.99±0.10 | 2.18 | 26097-80-3 | C_14_H_14_N_4_O_2_S | 302.35 |  | 9.1 Å ×4.7 Å×5.0 Å |
| 491 | Oxfendazole | 98.0 | 10.27±0.10 | 2.13 | 53716-50-0 | C_15_H_13_N_3_O_3_S | 315.35 |  | 9.1 Å ×10.7 Å×6.5 Å |
| 492 | Fenbendazole sulfone | 95.0 | 10.14±0.10 | 2.17 | 54029-20-8 | C_15_H_13_N_3_O_4_S | 331.35 |  | 8.4 Å ×12.4 Å×7.4 Å |
| 493 | Robenidine Hydrochloride | 98.0 | - | 4.9 | 25875-50-7 | C_15_H_14_Cl_3_N_5_ | 370.66 |  | 7.3 Å ×16.0 Å×4.3 Å |
| 494 | Sudan blue 2 | 98.0 | 5.45±0.20 | 4.57 | 17354-14-2 | C_22_H_26_N_2_O_2_ | 350.45 |  | 11.9 Å ×15.1 Å×3.1 Å |
| 495 | Acid yellow 36 | 98.0 | - | - | 587-98-4 | C_18_H_14_N_3_NaO_3_S | 375.38 |  | 11.4 Å ×12.5 Å×5.6 Å |
| 496 | Indometacin | 98.0 | 4.5 | 4.27 | 53-86-1 | C_19_H_16_ClNO_4_ | 357.79 |  | 13.5 Å ×4.3 Å×5.3 Å |
| 497 | Triclabendazole | 98.0 | 7.91±0.10 | 5.53 | 68786-66-3 | C_14_H_9_Cl_3_N_2_OS | 359.66 |  | 6.7 Å ×9.7 Å×9.1 Å |
| 498 | Glipizide | 98.0 | 5.9 | 1.91 | 29094-61-9 | C_21_H_27_N_5_O_4_S | 445.54 |  | 11.0 Å ×15.7 Å×5.1 Å |
| 499 | Febantel | 99.0 | 7.60±0.46 | 1.53 | 58306-30-2 | C_2_H_22_N_4_O_6_S | 446.48 |  | 11.8 Å ×12.2 Å×8.7 Å |
| 500 | Glibenclamide | 98.0 | 5.3 | 3.75 | 10238-21-8 | C_23_H_28_ClN_3_O_5_S | 494 |  | 16.3 Å ×17.8 Å×5.4 Å |
| 501 | Valnemulin hydrochloride | 98.0 | - | - | 133868-46-9 | C_31_H_53_ClN_2_O_5_S | 601.28 |  | 15.8 Å ×7.1 Å×7.0 Å |
| 502 | Phenylethanolamine A | 98.0 | 14.02±0.20 | 3.27 | 1346746-81-3 | C_19_H_24_N_2_O_4_ | 344.41 |  | 14.6 Å ×10.0 Å×6.2 Å |
| 503 | Cimaterol | 98.0 | 13.75±0.20 | 0.35 | 54239-37-1 | [C_12_H_17_N_3_O](https://pubchem.ncbi.nlm.nih.gov/) | 219.28 |  | 7.8 Å ×7.5 Å×7.0 Å |
| 504 | Terbutaline | 95.0 | 8.70 | 0.67 | 23031-25-6 | C_12_H_19_NO_3_ | 225.28 |  | 8.3 Å ×8.1 Å×4.1 Å |
| 505 | Salbutamol | 99.0 | 9.99±0.31 | 0.64 | 34391-04-3 | C_13_H_21_NO_3_ | 239.31 |  | 9.0 Å ×7.3 Å×4.7 Å |
| 506 | Procaterol | 95.0 | - | 0.93 | 72332-33-3 | C_16_H_22_N_2_O_3_ | 290.36 |  | 7.6 Å ×9.8 Å×5.9 Å |
| 507 | Cimbuterol | 98.0 | 13.77±0.20 | 0.81 | 54239-39-3 | [C_13_H_19_N_3_O](https://pubchem.ncbi.nlm.nih.gov/) | 233.31 |  | 9.4 Å ×6.8 Å×6.5 Å |
| 508 | Clonidine | 98.0 | 8.10±0.50 | 1.57 | 4205-90-7 | C_9_H_9_Cl_2_N_3_ | 230.09 |  | 5.6 Å ×8.3 Å×3.9 Å |
| 509 | Fenoterol | 98.0 | 8.5 | 2.22 | 13392-18-2 | C_17_H_21_NO_4_ | 303.35 |  | 8.7 Å ×4.4 Å×9.9 Å |
| 510 | Clenproperol | 98.0 | 13.27±0.20 | 2.03 | 38339-11-6 | C_11_H_16_Cl_2_N_2_O | 263.16 |  | 7.4 Å ×7.0 Å×7.5 Å |
| 511 | Clorprenaline | 99.0 | 13.60±0.20 | 1.82 | 3811-25-4 | [C_11_H_16_ClNO](https://pubchem.ncbi.nlm.nih.gov/) | 213.70 |  | 7.2 Å ×7.1 Å×6.5 Å |
| 512 | Ractopamine | 98.0 | 9.97±0.26 | - | 97825-25-7 | C_18_H_23_NO_3_ | 301.38 |  | 13.1 Å ×9.4 Å×6.2 Å |
| 513 | lsoxsuprine | 95.0 | 9.96±0.26 | 2.35 | 395-28-8 | C_18_H_23_NO_3_ | 301.39 |  | 9.7 Å ×9.6 Å×8.2 Å |
| 514 | Clenbuterol | 99.0 | 13.29±0.20 | 2.00 | 37148-27-9 | [C_12_H_18_Cl_2_N_2_O](https://pubchem.ncbi.nlm.nih.gov/) | 277.19 |  | 8.9 Å ×8.1 Å×5.8 Å |
| 515 | Tulobuterol | 98.0 | 13.62±0.20 | 2.27 | 41570-61-0 | [C_12_H_18_ClNO](https://pubchem.ncbi.nlm.nih.gov/) | 227.73 |  | 6.3 Å ×7.8 Å×6.6 Å |
| 516 | Formoterol | 98.0 | 8.95±0.50 | 1.40 | 73573-87-2 | C_19_H_24_N_2_O_4_ | 344.40 |  | 11.9 Å ×10.0 Å×9.0 Å |
| 517 | Clencyclohexerol | 98.0 | 13.26±0.20 | 1.78 | 157877-79-7 | C_14_H_20_Cl_2_N_2_O_2_ | 319.23 |  | 8.2 Å ×7.0 Å×9.5 Å |
| 518 | Brombuterol | 98.0 | - | - | [21912-49-2](https://www.chemsrc.com/baike/1463215.html) | C_12_H_19_Br_2_ClN_2_O | 402.56 |  | 7.1 Å ×11.8 Å×6.7 Å |
| 519 | Clenpenterol | 98.0 | 13.28±0.20 | 2.03 | 37158-47-7 | C_13_H_21_Cl_3_N_2_O | 327.67 |  | 7.4 Å ×7.0 Å×7.5 Å |
| 520 | Bambuterol | 98.0 | 13.70±0.20 | 1.49 | 81732-65-2 | [C_18_H_29_N_3_O_5_](https://pubchem.ncbi.nlm.nih.gov/) | 367.45 |  | 7.9 Å ×10.7 Å×9.2 Å |
| 521 | Clenhexerol | 98.0 | 13.29±0.20 | 2.98 | 38339-23-0 | C_14_H_22_Cl_2_N_2_O | 305.24 |  | 9.9 Å ×7.1 Å×4.7 Å |
| 522 | Cyproheptadine | 98.0 | 8.87 | 4.69 | 129-03-3 | C_21_H_21_N | 287.4 |  | 9.6 Å ×9.5 Å×7.0 Å |
| 523 | Salmeterol | 99.0 | 9.99±0.31 | 4.15 | 89365-50-4 | C_25_H_37_NO_4_ | 415.57 |  | 18.6 Å ×9.3 Å×15.1 Å |
| 524 | Amiloride | 98.0 | 8.7 | 0.09 | 2609-46-3 | C_6_H_8_ClN_7_O | 229.63 |  | 8.8 Å ×7.2 Å×0.7 Å |
| 525 | Trenbolone | 98.0 | 14.73±0.40 | 2.65 | 10161-33-8 | C_18_H_22_O_2_ | 270.37 |  | 10.3 Å ×7.6 Å×3.7 Å |
| 526 | Levonorgestrel | 98.0 | 13.09±0.40 | 3.48 | 797-63-7 | C_21_H_28_O_2_ | 312.45 |  | 8.5 Å ×6.4 Å×10.5 Å |
| 527 | Megestrol acetate | 98.0 | - | 4.00 | 595-33-5 | C_24_H_32_O_4_ | 384.51 |  | 10.7 Å ×8.0 Å×8.0 Å |
| 528 | Androstendione | 99.0 | - | 2.76 | 63-05-8 | C_19_H_26_O_2_ | 286.41 |  | 7.6 Å ×6.4 Å×8.4 Å |
| 529 | Beclomethasone | 98.0 | 12.17±0.70 | 2.03 | 4419-39-0 | C_22_H_29_ClO_5_ | 408.92 |  | 11.4 Å ×9.3 Å×5.6 Å |
| 530 | Danazol | 98.0 | 13.10±0.60 | 0.51 | 17230-88-5 | C_22_H_27_NO_2_ | 337.46 |  | 11.0 Å ×7.5 Å×7.8 Å |
| 531 | Methylandrostendiol | 98.0 | 15.01±0.70 | 4.35 | 521-10-8 | C_20_H_32_O_2_ | 304.47 |  | 8.8 Å ×7.5 Å×9.1 Å |
| 532 | Betamethasone | 98.0 | 12.13±0.70 | 2.01 | 378-44-9 | C_22_H_29_FO_5_ | 392.47 |  |  |
| 533 | Corticosterone | 98.0 | - | 1.99 | 50-22-6 | C_21_H_30_O_4_ | 346.47 |  | 11.3 Å ×8.8 Å×6.1 Å |
| 534 | testosterone | 98.0 | 15.06±0.60 | 3.27 | 58-22-0 | C_19_H_28_O_2_ | 288.43 |  | 10.1 Å ×7.5 Å×5.5 Å |
| 535 | Indapamide | 98.0 | 8.8 ± 0.2 | 2.2 | 26807-65-8 | C_16_H_16_ClN_3_O_3_S | 365.83 |  | 9.2 Å ×8.0 Å×7.8 Å |
| 536 | Canrenone | 98.0 | - | 3.34 | 976-71-6 | C_22_H_28_O_3_ | 340.46 |  | 6.1 Å ×12.6 Å×4.6 Å |
| 537 | Chlorthalidone | 98.0 | 10.959 | 0.85 | 77-36-1 | C_14_H_11_ClN_2_O_4_S | 338.77 |  | 5.2 Å ×7.3 Å×4.4 Å |
| 538 | Triamterene | 99.0 | 6.2 | 0.80 | 396-01-0 | C_12_H_11_N_7_ | 253.26 |  | 7.0 Å ×10.0 Å×6.2 Å |
| 539 | Nandrolone Phenylpropionate | 99.0 | - | 6.02 | 62-90-8 | C_27_H_34_O_3_ | 406.57 |  | 13.4 Å ×14.0 Å×5.7 Å |
| 540 | Testosterone propionate | 99.0 | - | 4.77 | 57-85-2 | C_22_H_32_O_3_ | 344.50 |  | 9.0 Å ×6.4 Å×11.0 Å |
| 541 | Nandrolone 17-propionate | 98.0 | - | 4.31 | 7207-92-3 | C_21_H_30_O_3_ | 330.46 |  | 11.7 Å ×10.4 Å×5.7 Å |
| 542 | Methyltestosterone | 95.0 | 15.14±0.60 | 3.59 | 65-04-3 | C_20_H_30_O_2_ | 302.45 |  | 8.1 Å ×6.4 Å×9.4 Å |
| 543 | Epiandrosterone | 99.0 | 15.14±0.60 | 3.07 | 481-29-8 | C_19_H_30_O_2_ | 290.45 |  | 9.0 Å ×7.9 Å×6.2 Å |
| 544 | Testosterone | 98.0 | 15.06±0.60 | 3.27 | 58-22-0 | C_19_H_28_O_2_ | 288.43 |  | 10.1 Å ×7.5 Å×5.5 Å |
| 545 | Boldenone | 98.0 | 15.05±0.60 | 3.05 | 846-48-0 | C_19_H_26_O_2_ | 286.41 |  | 10.3 Å ×7.5 Å×5.0 Å |
| 546 | Nandrolone | 98.0 | 15.06±0.40 | 2.82 | 434-22-0 | C_18_H_26_O_2_ | 274.40 |  | 10.1 Å ×7.5 Å×5.2 Å |
| 547 | Medroxyprogesterone | 98.0 | - | 4.09 | 71-58-9 | C_24_H_34_O_4_ | 386.52 |  | 9.5 Å ×6.4 Å×11.0 Å |
| 548 | Chlormadinone acetate | 98.0 | - | 3.95 | 302-22-7 | C_23_H_29_ClO_4_ | 404.93 |  | 10.6 Å ×7.7 Å×7.8 Å |
| 549 | Testosterone | 98.0 | 15.06±0.60 | 3.32 | 58-22-0 | C_19_H_28_O_2_ | 288.43 |  | 10.1 Å ×7.5 Å×5.5 Å |
| 550 | Aspirin | 99.0 | 3.5 | 1.13 | 50-78-2 | C_9_H_8_O_4_ | 180.16 |  | 5.3 Å ×7.4 Å×4.3 Å |
| 551 | Rafoxanide | 98.0 | 6.04±0.48 | 7.82 | 22662-39-1 | C_19_H_11_Cl_2_I_2_NO_3_ | 626.01 |  | 16.1 Å ×15.0 Å×2.8 Å |
| 552 | Doramectin | 98.0 | 12.42±0.70 | - | 117704-25-3 | C_50_H_74_O_14_ | 899.11 |  | 20.1 Å ×16.9 Å×7.8 Å |

Table S6. Mass spectrometric parameters of pesticides and veterinary drugs.

| Serial  number | Analytes | *tR*  [min] | Ions pairs [m/z] | | Collision energy [eV] | | Cone voltage [V] |
| --- | --- | --- | --- | --- | --- | --- | --- |
|  |  |  | Quantitative | Qualification | Quantitative | Qualification |  |
| 1 | Acephate | 2.99 | 184.02/143.00 | 184.02/125.00 | 8 | 20 | 18 |
| 2 | Acetamiprid | 3.92 | 223.07/126.00 | 223.07/56.10 | 20 | 20 | 34 |
| 3 | Acetochlor | 13.62 | 270.10/148.00 | 270.10/224.00 | 20 | 10 | 25 |
| 4 | Alachlor | 13.67 | 270.13/238.10 | 270.13/162.10 | 12 | 20 | 20 |
| 5 | Albendazole | 9.48 | 265.90/191.20 | 265.20/234.10 | 32 | 20 | 36 |
| 6 | Aldicarb | 4.69 | 208.10/116.10 | 208.10/89.00 | 7 | 16 | 10 |
| 7 | Aldicarb sulfone | 3.29 | 223.09/86.00 | 223.09/76.00 | 20 | 20 | 30 |
| 8 | Aldicarb sulfoxide | 3.29 | 207.08/89.00 | 207.08/132.00 | 15 | 10 | 20 |
| 9 | Ametoctradin | 18.67 | 276.22/149.00 | 276.22/176.00 | 36 | 38 | 20 |
| 10 | Amidosulfuron | 6.02 | 370.04/261.00 | 370.04/218.00 | 15 | 25 | 10 |
| 11 | Amisulbrom | 20.17 | 466.00/108.02 | 466.00/226.97 | 20 | 20 | 25 |
| 12 | Anilofos | 16.60 | 368.00/124.90 | 368.00/198.90 | 30 | 15 | 25 |
| 13 | Atrazine | 7.44 | 216.10/174.10 | 216.10/96.10 | 18 | 22 | 39 |
| 14 | Avermectin B1a | 23.25 | 890.40/305.20 | 890.40/567.20 | 26 | 14 | 40 |
| 15 | Azinphos-methyl | 8.91 | 318.01/125.00 | 318.01/261.00 | 24 | 6 | 20 |
| 16 | Azoxystrobin | 10.25 | 404.12/372.00 | 404.12/329.00 | 15 | 30 | 28 |
| 17 | Benalaxyl | 17.12 | 326.17/148.00 | 326.17/91.00 | 20 | 34 | 20 |
| 18 | Bendiocarb | 5.50 | 224.09/167.00 | 224.09/109.00 | 8 | 18 | 30 |
| 19 | Bensulfuron-methyl | 9.30 | 411.08/149.00 | 411.08/182.00 | 22 | 20 | 30 |
| 20 | Benzovindiflupyr | 16.38 | 398.00/342.00 | 398.00/378.00 | 18 | 14 | 40 |
| 21 | Benzoximate | 18.25 | 364.10/199.10 | 364.10/105.00 | 18 | 20 | 20 |
| 22 | Bifenox | 18.51 | 359.00/309.91 | 359.00/342.00 | 11 | 6 | 30 |
| 23 | Bifenthrin | 23.58 | 440.10/181.10 | 440.10/166.10 | 11 | 45 | 20 |
| 24 | Bioresmethrin | 23.02 | 339.00/171.00 | 339.00/128.00 | 15 | 40 | 30 |
| 25 | Bitertanol | 17.86 | 338.19/70.10 | 338.19/99.10 | 12 | 18 | 21 |
| 26 | Boscalid | 10.86 | 343.04/140.00 | 343.04/307.00 | 20 | 20 | 32 |
| 27 | Bromuconazole | 12.18 | 375.96/159.00 | 375.96/70.00 | 27 | 20 | 40 |
| 28 | Bupirimate | 14.53 | 317.16/108.00 | 317.16/166.00 | 25 | 25 | 10 |
| 29 | Buprofenzin | 21.13 | 306.16/201.00 | 306.16/116.00 | 10 | 15 | 5 |
| 30 | Butachlor | 21.34 | 312.20/238.20 | 312.20/57.30 | 10 | 15 | 15 |
| 31 | Butralin | 22.17 | 296.20/240.00 | 296.20/222.00 | 12 | 20 | 20 |
| 32 | Cadusafos | 19.23 | 271.10/131.00 | 271.10/159.00 | 20 | 15 | 20 |
| 33 | Carbaryl | 6.03 | 202.09/145.00 | 202.09/117.00 | 28 | 22 | 28 |
| 34 | Carbendazim | 3.87 | 192.08/160.05 | 192.08/132.00 | 18 | 30 | 25 |
| 35 | Carbofuran | 5.61 | 222.11/165.06 | 222.11/123.00 | 16 | 16 | 30 |
| 36 | Carbofuran-3-hydroxy | 3.93 | 238.11/181.00 | 238.11/163.00 | 12 | 20 | 34 |
| 37 | Carboxin | 6.15 | 236.00/143.00 | 236.00/87.00 | 15 | 25 | 30 |
| 38 | Carfentrazone-ethyl | 16.00 | 429.00/412.00 | 429.00/346.00 | 10 | 27 | 10 |
| 39 | Chlorantranilipol | 9.18 | 483.98/453.00 | 483.98/286.00 | 18 | 20 | 30 |
| 40 | Chlorbenzuron | 15.48 | 309.00/139.00 | 309.00/156.00 | 30 | 15 | 30 |
| 41 | Chlordimeform | 3.50 | 197.10/117.10 | 197.10/125.00 | 25 | 32 | 30 |
| 42 | Chlorfenvinphos | 17.14 | 358.90/99.00 | 358.90/155.00 | 30 | 10 | 25 |
| 43 | Chlorfluazuron | 22.44 | 539.90/382.90 | 539.90/158.00 | 20 | 20 | 20 |
| 44 | Chloridazon | 4.09 | 222.12/91.76 | 222.12/103.82 | 24 | 20 | 10 |
| 45 | Chlorimuron ethyl | 11.33 | 415.10/186.00 | 415.10/83.00 | 15 | 40 | 35 |
| 46 | Chlorpropham | 7.72 | 214.06/172.00 | 214.06/154.00 | 8 | 18 | 18 |
| 47 | Chlorpyrifos | 21.55 | 350.10/97.00 | 350.10/197.90 | 30 | 25 | 30 |
| 48 | Chlorpyriphos-methyl | 18.37 | 321.90/125.00 | 321.90/289.90 | 20 | 15 | 25 |
| 49 | Chlorsulfuron | 5.88 | 358.00/141.00 | 358.00/167.00 | 16 | 18 | 30 |
| 50 | Chlortoluron | 6.92 | 213.00/46.00 | 213.00/72.00 | 15 | 15 | 25 |
| 51 | Chromafenozide | 13.82 | 395.23/175.00 | 395.23/339.10 | 22 | 12 | 30 |
| 52 | Cinosulfuron | 4.96 | 414.00/183.00 | 414.00/157.00 | 16 | 24 | 35 |
| 53 | Clethodim | 20.35 | 360.14/164.00 | 360.14/206.00 | 20 | 20 | 32 |
| 54 | Clethodim sulfone | 8.37 | 392.10/164.10 | 392.10/300.10 | 27 | 13 | 20 |
| 55 | Clethodim sulfoxide | 8.60 | 376.10/206.10 | 376.10/164.10 | 15 | 21 | 10 |
| 56 | Clofentezine | 17.56 | 303.02/138.00 | 303.02/102.00 | 20 | 35 | 30 |
| 57 | Clomazone | 9.00 | 240.08/125.00 | 240.08/89.00 | 18 | 46 | 32 |
| 58 | Clothianidin | 3.77 | 250.02/169.00 | 250.02/132.00 | 14 | 18 | 30 |
| 59 | Coumaphos | 17.12 | 363.02/227.00 | 363.02/307.00 | 24 | 18 | 32 |
| 60 | Coumoxystrobin | 21.45 | 437.10/205.00 | 437.10/145.00 | 8 | 24 | 40 |
| 61 | Cyanazine | 5.12 | 241.10/214.00 | 241.10/96.00 | 18 | 30 | 41 |
| 62 | Cyantraniliprole | 6.53 | 473.00/284.00 | 473.00/442.00 | 13 | 16 | 40 |
| 63 | Cyazofamid | 13.94 | 325.00/107.90 | 325.00/261.00 | 40 | 15 | 15 |
| 64 | Cyclosulfamuron | 13.09 | 422.20/261.10 | 422.20/218.10 | 15 | 25 | 15 |
| 65 | Cycloxydim | 20.18 | 326.17/280.00 | 326.17/180.00 | 10 | 20 | 25 |
| 66 | Cyflufenamid | 18.83 | 413.19/295.11 | 413.19/203.00 | 15 | 40 | 15 |
| 67 | Cyflumetofen | 20.92 | 465.00/173.00 | 465.00/249.00 | 20 | 13 | 10 |
| 68 | Cymoxanil | 4.23 | 199.02/127.88 | 199.02/110.90 | 10 | 14 | 15 |
| 69 | Cyproconazole | 12.32 | 292.12/70.10 | 292.12/125.10 | 20 | 22 | 30 |
| 70 | Deltamethrin | 22.63 | 523.00/280.90 | 523.00/506.00 | 16 | 10 | 10 |
| 71 | Demeton | 9.32 | 259.00/61.10 | 259.00/89.00 | 33 | 10 | 30 |
| 72 | Demeton-S-methyl | 5.75 | 231.10/89.10 | 231.10/61.20 | 25 | 10 | 12 |
| 73 | Demeton-S-methyl sulfone | 3.46 | 263.02/169.00 | 263.02/121.00 | 18 | 18 | 32 |
| 74 | Demeton-S-sulfone | 4.13 | 291.00/95.00 | 291.00/235.00 | 28 | 14 | 20 |
| 75 | Demeton-S-sulfoxide | 4.04 | 275.10/104.90 | 275.10/140.80 | 16 | 24 | 25 |
| 76 | Diazinon | 17.42 | 305.11/169.00 | 305.11/97.00 | 22 | 40 | 31 |
| 77 | Dichlorvos | 5.37 | 221.00/109.00 | 221.00/79.00 | 15 | 25 | 20 |
| 78 | Diclobutrazol | 15.50 | 328.10/70.00 | 328.10/159.00 | 20 | 38 | 15 |
| 79 | Diclofop methyl | 20.80 | 357.90/120.00 | 357.90/280.90 | 25 | 10 | 25 |
| 80 | Dicrotophos | 3.60 | 238.08/112.00 | 238.08/193.00 | 18 | 10 | 28 |
| 81 | Diethofencarb | 9.97 | 268.16/226.00 | 268.16/180.00 | 15 | 22 | 30 |
| 82 | Diethyl aminoethyl hexanoate | 3.84 | 216.20/100.20 | 216.20/143.00 | 15 | 15 | 40 |
| 83 | Difenoconazole | 19.07 | 406.07/251.00 | 406.07/337.00 | 25 | 18 | 37 |
| 84 | Diflubenzuron | 14.23 | 311.04/141.00 | 311.04/158.00 | 30 | 15 | 25 |
| 85 | Diflufenican | 19.82 | 395.08/266.00 | 395.08/246.00 | 24 | 32 | 26 |
| 86 | Dimepiperate | 19.32 | 264.14/146.10 | 264.14/119.10 | 12 | 15 | 8 |
| 87 | Dimethenamid | 10.53 | 276.08/244.00 | 276.08/168.00 | 15 | 26 | 26 |
| 88 | Dimethoate | 3.99 | 230.01/199.00 | 230.01/125.00 | 10 | 20 | 24 |
| 89 | Dimethomorph | 11.30 | 388.13/301.00 | 388.13/165.00 | 22 | 30 | 30 |
| 90 | Dimoxystrobin | 15.20 | 327.10/116.10 | 327.10/205.20 | 20 | 10 | 15 |
| 91 | Diniconazole | 18.10 | 326.08/70.00 | 326.08/159.00 | 25 | 33 | 46 |
| 92 | Dinotefuran | 3.21 | 203.11/129.00 | 203.11/157.00 | 20 | 10 | 15 |
| 93 | Dinocap | 21.16 | 295.00/134.00 | 295.00/209 | 55 | 28 | 30 |
| 94 | Disulfoton | 18.27 | 275.10/61.00 | 275.10/89.00 | 32 | 10 | 2 |
| 95 | Disulfoton sulfone | 7.18 | 307.03/97.00 | 307.03/153.00 | 28 | 12 | 24 |
| 96 | Disulfoton sulfoxide | 6.91 | 291.03/157.00 | 291.03/185.00 | 20 | 18 | 24 |
| 97 | Diuron | 7.82 | 233.03/72.00 | 233.03/46.00 | 15 | 18 | 30 |
| 98 | EPN | 19.27 | 324.00/157.00 | 324.00/296.00 | 20 | 10 | 10 |
| 99 | Edifenphos | 16.37 | 311.00/109.00 | 311.00/111.00 | 35 | 25 | 30 |
| 100 | Emamectin B1a | 21.16 | 886.50/82.00 | 886.50/158.00 | 55 | 35 | 50 |
| 101 | Enestroburin | 20.98 | 400.00/137.00 | 400.00/178.00 | 27 | 14 | 20 |
| 102 | Epoxiconazole | 13.79 | 330.00/101.00 | 330.00/121.04 | 40 | 20 | 40 |
| 103 | Ethion | 21.49 | 384.99/142.90 | 384.99/199.00 | 25 | 10 | 25 |
| 104 | Ethiprole | 10.65 | 414.10/396.90 | 414.10/350.90 | 20 | 25 | 5 |
| 105 | Ethofumesate | 9.92 | 287.10/121.10 | 287.10/259.10 | 20 | 20 | 32 |
| 106 | Ethoprophos | 13.42 | 243.06/131.00 | 243.06/173.00 | 20 | 20 | 32 |
| 107 | Ethoxysulfuron | 11.84 | 399.10/261.00 | 399.10/218.00 | 18 | 24 | 25 |
| 108 | Etofenprox | 23.53 | 394.30/177.00 | 394.30/359.20 | 15 | 9 | 20 |
| 109 | Etoxazole | 22.12 | 360.18/141.10 | 360.18/304.10 | 25 | 20 | 60 |
| 110 | Etrimfos | 15.89 | 293.10/125.00 | 293.10/265.10 | 25 | 20 | 30 |
| 111 | Famoxadone | 17.42 | 392.20/238.00 | 392.20/331.10 | 20 | 10 | 30 |
| 112 | Fenamidone | 10.48 | 312.12/92.00 | 312.12/236.10 | 25 | 15 | 30 |
| 113 | Fenaminstrobin | 20.32 | 434.00/171.00 | 434.00/212.00 | 27 | 15 | 10 |
| 114 | Fenamiphos | 14.79 | 304.10/202.10 | 304.10/217.10 | 35 | 25 | 30 |
| 115 | Fenamiphos sulphone | 5.92 | 336.10/188.20 | 336.10/266.10 | 25 | 20 | 15 |
| 116 | Fenamiphos sulphoxide | 5.64 | 320.10/108.00 | 320.10/171.10 | 35 | 20 | 15 |
| 117 | Fenarimol | 12.97 | 331.04/268.00 | 331.04/81.00 | 22 | 30 | 40 |
| 118 | Fenazaquin | 22.78 | 307.18/57.10 | 307.18/161.00 | 20 | 15 | 15 |
| 119 | Fenbuconazole | 14.22 | 337.12/70.00 | 337.12/125.00 | 22 | 30 | 29 |
| 120 | Fenhexamid | 13.00 | 302.07/97.00 | 302.07/55.00 | 22 | 38 | 32 |
| 121 | Fenobucarb | 9.40 | 208.13/95.00 | 208.13/152.00 | 14 | 10 | 30 |
| 122 | Fenothiocarb | 14.56 | 254.20/72.00 | 254.20/160.10 | 27 | 15 | 30 |
| 123 | Fenoxanil | 15.08 | 329.08/86.15 | 329.08/302.15 | 20 | 10 | 25 |
| 124 | Fenoxaprop-ethyl | 20.62 | 362.13/121.10 | 362.13/288.08 | 25 | 15 | 40 |
| 125 | Fenoxycarb | 14.78 | 302.13/88.00 | 302.13/116.10 | 20 | 15 | 30 |
| 126 | Fenpropathrin | 22.23 | 350.10/97.00 | 350.10/125.00 | 34 | 14 | 30 |
| 127 | Fenpyrazamine | 12.54 | 332.14/230.10 | 332.14/272.10 | 20 | 20 | 25 |
| 128 | Fenpyroximate | 22.43 | 422.20/366.10 | 422.20/138.10 | 16 | 32 | 32 |
| 129 | Fensulfothion | 7.74 | 309.04/157.00 | 309.04/173.00 | 25 | 22 | 36 |
| 130 | Fensulfothion oxon | 4.39 | 293.00/237.00 | 293.00/265.00 | 20 | 15 | 25 |
| 131 | Fensulfothion oxon sulfone | 4.51 | 309.00/175.00 | 309.00/253.00 | 25 | 25 | 28 |
| 132 | Fenthion | 15.81 | 279.00/104.90 | 279.00/168.90 | 25 | 15 | 25 |
| 133 | Fenthion sulphone | 6.30 | 311.00/109.00 | 311.00/125.00 | 25 | 20 | 20 |
| 134 | Fenthion sulfoxide | 19.25 | 295.00/109.00 | 295.00/280.00 | 30 | 20 | 45 |
| 135 | Fenvalerate | 22.77 | 437.00/167.00 | 437.00/420.00 | 16 | 8 | 30 |
| 136 | Fipronil | 15.28 | 434.93/330.00 | 434.93/250.00 | 15 | 30 | 24 |
| 137 | Fipronil desulfinyl | 14.33 | 386.90/282.00 | 386.90/351.00 | 30 | 15 | 35 |
| 138 | Fipronil sulphide | 16.14 | 418.93/383.00 | 418.93/314.00 | 15 | 22 | 35 |
| 139 | Fipronil sulphone | 17.32 | 450.94/282.00 | 450.94/415.00 | 25 | 20 | 30 |
| 140 | Flonicamid | 3.50 | 230.00/148.05 | 230.00/203.07 | 25 | 15 | 35 |
| 141 | Florasulam | 4.41 | 360.00/108.90 | 360.00/129.00 | 50 | 35 | 40 |
| 142 | Fluazafop-P-butyl | 21.06 | 384.14/282.10 | 384.14/328.10 | 22 | 16 | 27 |
| 143 | Fluazinam | 21.31 | 462.80/398.00 | 462.80/416.00 | 15 | 17 | 20 |
| 144 | Flubendiamide | 16.91 | 681.00/254.00 | 681.00/274.00 | 28 | 14 | 60 |
| 145 | Flucetosulfuron | 9.70 | 488.12/156.07 | 488.12/273.08 | 20 | 25 | 30 |
| 146 | Flucythrinate | 22.04 | 469.00/412.00 | 469.00/199.00 | 13 | 18 | 10 |
| 147 | Fludioxonil | 10.35 | 266.00/229.00 | 266.00/158.00 | 10 | 35 | 5 |
| 148 | Flufenacet | 13.57 | 364.07/152.00 | 364.07/194.00 | 20 | 15 | 30 |
| 149 | Flufenoxuron | 22.05 | 489.04/158.00 | 489.04/141.00 | 22 | 46 | 31 |
| 150 | Flumetralin | 22.12 | 422.00/143.00 | 422.00/107.00 | 13 | 70 | 30 |
| 151 | Flumetsulam | 3.75 | 326.10/109.00 | 326.10/129.00 | 50 | 25 | 25 |
| 152 | Flumorph | 8.48 | 372.10/165.00 | 372.10/285.00 | 31 | 20 | 50 |
| 153 | Fluopicolide | 11.55 | 382.97/173.00 | 382.97/109.00 | 20 | 60 | 40 |
| 154 | Fluopyram | 13.00 | 397.00/173.20 | 397.00/208.10 | 30 | 35 | 15 |
| 155 | Fluoroglycofen-ethyl | 20.36 | 465.00/344.00 | 465.00/223.00 | 22 | 16 | 10 |
| 156 | Flurtamone | 10.16 | 334.10/178.00 | 334.10/247.00 | 45 | 25 | 40 |
| 157 | Flusilazole | 14.72 | 316.11/165.00 | 316.11/247.00 | 28 | 18 | 36 |
| 158 | Fluthiacet-methyl | 15.55 | 404.03/85.00 | 404.03/274.00 | 28 | 28 | 10 |
| 159 | Flutolanil | 11.49 | 324.11/262.10 | 324.11/282.10 | 18 | 18 | 34 |
| 160 | Flutriafol | 7.30 | 302.11/70.10 | 302.11/123.10 | 18 | 29 | 32 |
| 161 | Fluvalinate | 23.01 | 503.00/208.10 | 503.00/181.10 | 10 | 30 | 40 |
| 162 | Fluxapyroxad | 11.54 | 382.10/362.00 | 382.10/342.00 | 18 | 20 | 30 |
| 163 | Fonofos | 16.16 | 247.00/109.00 | 247.00/137.00 | 20 | 10 | 20 |
| 164 | Formothion | 4.87 | 258.00/125.00 | 258.00/199.00 | 22 | 5 | 10 |
| 165 | Forchlorfenuron | 7.91 | 248.06/129.00 | 248.06/93.00 | 15 | 35 | 36 |
| 166 | Fosthiazate | 6.78 | 284.05/104.00 | 284.05/228.00 | 21 | 15 | 19 |
| 167 | Furathiocarb | 21.09 | 383.16/195.00 | 383.16/252.00 | 20 | 15 | 30 |
| 168 | Heptenophos | 8.00 | 251.00/125.00 | 251.00/127.00 | 15 | 15 | 15 |
| 169 | Hexaconazole | 16.64 | 314.08/70.00 | 314.08/159.00 | 22 | 28 | 40 |
| 170 | Hexaflumuron | 20.34 | 460.90/158.10 | 460.90/141.10 | 15 | 40 | 45 |
| 171 | Hexazinone | 5.68 | 253.17/71.00 | 253.17/171.00 | 30 | 16 | 30 |
| 172 | Hexythiazox | 21.64 | 353.11/228.10 | 353.11/168.10 | 15 | 25 | 30 |
| 173 | Imazalil | 6.84 | 297.06/69.00 | 297.06/159.00 | 22 | 22 | 30 |
| 174 | Imidacloprid | 3.71 | 256.06/209.06 | 256.06/175.00 | 16 | 20 | 30 |
| 175 | Imidaclothiz | 3.82 | 262.00/122.00 | 262.00/181.10 | 30 | 13 | 30 |
| 176 | Indoxacarb | 20.02 | 528.08/150.00 | 528.08/249.00 | 22 | 20 | 30 |
| 177 | Iodosulfuron-methyl | 7.91 | 508.10/167.10 | 508.10/141.10 | 25 | 25 | 30 |
| 178 | Ipconazole | 19.87 | 334.17/70.00 | 334.17/125.00 | 25 | 25 | 50 |
| 179 | Iprobenfos | 15.70 | 289.00/91.00 | 289.00/205.00 | 20 | 10 | 35 |
| 180 | Iprodione | 14.19 | 330.00/245.00 | 330.00/288.00 | 14 | 10 | 35 |
| 181 | Iprovalicarb | 13.48 | 321.22/119.10 | 321.22/203.10 | 18 | 12 | 30 |
| 182 | Isazofos | 12.59 | 314.00/120.00 | 314.00/162.10 | 25 | 15 | 25 |
| 183 | Isocarbofos | 7.97 | 231.00/65.00 | 231.00/121.00 | 39 | 18 | 50 |
| 184 | Isofenphos-methyl | 16.34 | 332.00/230.90 | 332.00/272.90 | 15 | 8 | 20 |
| 185 | Isoprocarb | 7.22 | 194.11/95.10 | 194.11/137.10 | 14 | 10 | 24 |
| 186 | Isoprothiolane | 11.44 | 291.10/188.80 | 291.10/230.90 | 22 | 12 | 30 |
| 187 | Isoproturon | 7.62 | 207.15/72.00 | 207.15/165.00 | 22 | 22 | 30 |
| 188 | Isopyrazam | 19.27 | 360.20/244.13 | 360.20/320.20 | 20 | 20 | 20 |
| 189 | Isoxaflutole | 7.94 | 377.00/251.00 | 377.00/360.00 | 20 | 8 | 20 |
| 190 | Ivermectin B1a | 23.99 | 892.50/569.30 | 892.50/307.20 | 15 | 20 | 30 |
| 191 | Kresoxim-methyl | 23.06 | 314.00/116.00 | 314.00/131.00 | 30 | 20 | 30 |
| 192 | Lactofen | 21.23 | 479.20/223.00 | 479.20/343.10 | 35 | 15 | 20 |
| 193 | Linuron | 9.54 | 249.02/160.00 | 249.02/182.00 | 20 | 18 | 30 |
| 194 | Lufenuron | 21.63 | 511.00/158.00 | 511.00/141.00 | 20 | 45 | 35 |
| 195 | Malaoxon | 5.73 | 315.06/127.00 | 315.06/99.00 | 12 | 24 | 24 |
| 196 | Malathion | 11.66 | 331.05/127.00 | 331.05/285.00 | 14 | 10 | 20 |
| 197 | Mandipropamid | 11.46 | 411.80/125.00 | 411.80/328.10 | 30 | 15 | 20 |
| 198 | Mefenacet | 12.24 | 299.09/148.00 | 299.09/120.00 | 18 | 22 | 25 |
| 199 | Mepronil | 11.66 | 270.10/91.00 | 270.10/119.00 | 35 | 25 | 35 |
| 200 | Metaflumizone | 21.28 | 507.00/178.00 | 507.00/116.00 | 25 | 40 | 30 |
| 201 | Metalaxyl | 7.99 | 280.16/220.10 | 280.16/192.10 | 15 | 18 | 26 |
| 202 | Metamifop | 20.89 | 441.10/180.10 | 441.10/288.10 | 27 | 23 | 30 |
| 203 | Metamitron | 4.03 | 203.09/104.00 | 203.09/175.00 | 20 | 16 | 16 |
| 204 | Metazachlor | 7.67 | 278.10/134.10 | 278.10/210.00 | 20 | 10 | 20 |
| 205 | Metconazole | 17.26 | 320.15/70.00 | 320.15/125.00 | 25 | 30 | 20 |
| 206 | Methacrifos | 8.53 | 241.10/209.10 | 241.10/125.00 | 8 | 20 | 25 |
| 207 | Methamidophos | 2.71 | 142.00/93.90 | 142.00/124.90 | 15 | 15 | 25 |
| 208 | Methidathion | 8.48 | 302.97/85.00 | 302.97/145.00 | 20 | 10 | 18 |
| 209 | Methiocarb | 9.90 | 226.09/169.00 | 226.09/121.00 | 10 | 22 | 28 |
| 210 | Methiocarb sulfone | 3.98 | 258.07/122.10 | 258.07/107.10 | 20 | 35 | 40 |
| 211 | Methiocarb sulfoxide | 3.79 | 242.08/185.00 | 242.08/122.00 | 15 | 25 | 26 |
| 212 | Methomyl | 3.49 | 163.06/88.00 | 163.06/106.00 | 10 | 10 | 26 |
| 213 | Methoxyfenozide | 12.45 | 369.22/149.10 | 369.22/313.20 | 20 | 10 | 30 |
| 214 | Metolachlor | 14.13 | 284.14/252.10 | 284.14/176.10 | 18 | 25 | 26 |
| 215 | Metolcarb | 5.01 | 166.09/109.06 | 166.09/94.00 | 12 | 27 | 30 |
| 216 | Metrafenone | 18.60 | 409.00/209.10 | 409.00/226.90 | 15 | 30 | 20 |
| 217 | Metribuzin | 5.67 | 215.10/89.00 | 215.10/131.00 | 15 | 20 | 40 |
| 218 | Metsulfuron-methyl | 5.37 | 382.00/167.00 | 382.00/198.90 | 15 | 20 | 15 |
| 219 | Mevinphos | 4.32 | 225.10/127.10 | 225.10/193.10 | 15 | 10 | 20 |
| 220 | Molinate | 11.39 | 188.11/126.00 | 188.11/55.00 | 13 | 25 | 17 |
| 221 | Monocrotophos | 3.55 | 224.07/127.00 | 224.07/98.10 | 16 | 15 | 26 |
| 222 | Myclobutanil | 11.72 | 289.12/70.10 | 289.12/125.10 | 18 | 22 | 34 |
| 223 | Napropamide | 13.57 | 272.17/129.10 | 272.17/171.10 | 18 | 20 | 30 |
| 224 | Nitenpyram | 3.34 | 271.09/126.00 | 271.09/99.00 | 20 | 22 | 30 |
| 225 | Novaluron | 20.74 | 493.02/141.00 | 493.02/158.03 | 40 | 20 | 45 |
| 226 | Omethoate | 3.13 | 214.03/183.00 | 214.03/125.00 | 11 | 22 | 26 |
| 227 | Orthosulfamuron | 7.84 | 425.10/199.10 | 425.10/227.10 | 10 | 12 | 20 |
| 228 | Oxadiargyl | 17.77 | 358.00/223.00 | 358.00/341.00 | 20 | 8 | 30 |
| 229 | Oxadiazon | 21.29 | 345.10/177.00 | 345.10/219.90 | 25 | 15 | 35 |
| 230 | Oxadixyl | 4.79 | 279.13/219.10 | 279.13/132.00 | 10 | 34 | 40 |
| 231 | Oxamyl | 3.33 | 237.10/72.00 | 237.10/90.00 | 15 | 15 | 30 |
| 232 | Oxamyl-oxime | 3.15 | 163.05/72.00 | 163.05/90.00 | 12 | 16 | 26 |
| 233 | Oxaziclomefone | 20.78 | 376.09/190.00 | 376.09/161.00 | 16 | 30 | 30 |
| 234 | Oxydemeton-methyl | 3.40 | 247.02/169.00 | 247.02/105.00 | 15 | 25 | 26 |
| 235 | Oxyfluorfen | 20.62 | 362.00/316.00 | 362.00/237.00 | 15 | 25 | 20 |
| 236 | Paclobutrazol | 11.19 | 294.10/70.20 | 294.10/125.10 | 15 | 40 | 20 |
| 237 | Parathion | 15.00 | 291.90/110.00 | 291.90/236.00 | 25 | 15 | 15 |
| 238 | Penconazole | 15.33 | 284.07/70.00 | 284.07/159.00 | 18 | 30 | 34 |
| 239 | Pencycuron | 18.67 | 329.14/125.10 | 329.14/218.10 | 22 | 18 | 30 |
| 240 | Pendimethalin | 21.64 | 282.15/212.10 | 282.15/194.10 | 12 | 18 | 21 |
| 241 | Penflufen | 15.67 | 318.00/141.00 | 318.00/234.00 | 30 | 15 | 15 |
| 242 | Penoxsulam | 6.35 | 484.07/164.10 | 484.07/195.10 | 35 | 25 | 45 |
| 243 | Penthiopyrad | 16.23 | 360.10/177.10 | 360.10/276.00 | 30 | 15 | 25 |
| 244 | Permethrin | 23.36 | 408.00/183.00 | 408.00/355.00 | 16 | 7 | 20 |
| 245 | Phenamacril | 5.23 | 217.00/104.00 | 217.00/189.00 | 21 | 10 | 40 |
| 246 | Phenmedipham | 9.94 | 301.11/168.00 | 301.11/136.00 | 15 | 20 | 30 |
| 247 | Phenthoate | 15.58 | 321.00/135.00 | 321.00/163.00 | 20 | 12 | 30 |
| 248 | Phorate | 17.36 | 261.00/75.10 | 261.00/199.00 | 15 | 5 | 15 |
| 249 | Phorate sulfone | 7.25 | 293.01/115.00 | 293.01/171.00 | 20 | 20 | 24 |
| 250 | Phorate sulfoxide | 6.92 | 277.02/143.00 | 277.02/171.00 | 20 | 18 | 24 |
| 251 | Phosalone | 18.05 | 367.99/181.90 | 367.99/110.90 | 18 | 42 | 22 |
| 252 | Phosfolan | 4.52 | 256.02/140.00 | 256.02/168.00 | 20 | 20 | 26 |
| 253 | Phosfolan methyl | 3.58 | 228.00/168.00 | 228.00/61.00 | 20 | 22 | 25 |
| 254 | Phosmet | 9.14 | 318.00/160.00 | 318.00/77.00 | 25 | 30 | 10 |
| 255 | Phosmet-Oxon | 4.74 | 302.00/160.00 | 302.00/77.00 | 15 | 50 | 15 |
| 256 | Phosphamidon | 5.03 | 300.08/127.00 | 300.08/174.00 | 20 | 15 | 25 |
| 257 | Phoxim | 17.65 | 299.00/129.00 | 299.00/153.00 | 15 | 10 | 25 |
| 258 | Picolinafen | 21.12 | 377.09/359.00 | 377.09/266.00 | 20 | 29 | 38 |
| 259 | Picoxystrobin | 15.34 | 368.00/145.10 | 368.00/205.10 | 27 | 13 | 10 |
| 260 | Pirimicarb | 6.46 | 239.15/182.10 | 239.15/72.00 | 20 | 30 | 30 |
| 261 | Piperonyl butoxide | 21.40 | 356.24/177.00 | 356.24/119.00 | 10 | 35 | 20 |
| 262 | Pirimicarb desmethyl | 4.29 | 225.23/71.73 | 225.23/167.95 | 16 | 14 | 2 |
| 263 | Pirimiphos-methyl | 17.97 | 306.10/108.10 | 306.10/164.10 | 32 | 25 | 36 |
| 264 | Pretilachlor | 20.13 | 312.00/176.00 | 312.00/252.00 | 25 | 15 | 30 |
| 265 | Probenazole | 5.07 | 224.03/41.20 | 224.03/196.10 | 10 | 15 | 20 |
| 266 | Prochloraz | 17.47 | 376.03/265.90 | 376.03/307.90 | 15 | 10 | 25 |
| 267 | Procymidone | 12.67 | 284.10/256.00 | 284.10/95.00 | 15 | 23 | 40 |
| 268 | Profenofos | 20.40 | 372.90/127.90 | 372.90/302.60 | 45 | 20 | 40 |
| 269 | Promecarb | 10.57 | 208.14/109.00 | 208.14/151.00 | 18 | 10 | 30 |
| 270 | Prometryn | 12.92 | 242.20/158.00 | 242.20/200.10 | 25 | 15 | 40 |
| 271 | Propachlor | 7.72 | 212.08/170.00 | 212.08/94.00 | 18 | 20 | 31 |
| 272 | Propamocarb | 3.05 | 189.16/102.00 | 189.16/144.00 | 17 | 12 | 30 |
| 273 | Propanil | 9.62 | 218.00/127.00 | 218.00/161.90 | 25 | 15 | 25 |
| 274 | Propaquizafop | 21.18 | 444.20/100.04 | 444.20/163.10 | 30 | 50 | 35 |
| 275 | Propargite | 22.08 | 368.19/175.00 | 368.19/231.00 | 20 | 20 | 30 |
| 276 | Propiconazole | 16.51 | 342.08/159.00 | 342.08/69.00 | 20 | 20 | 37 |
| 277 | Propoxur | 5.53 | 210.11/111.00 | 210.11/168.00 | 15 | 10 | 30 |
| 278 | Propyrisulfuron | 12.06 | 456.00/196.00 | 456.00/261.00 | 14 | 16 | 20 |
| 279 | Propyzamide | 11.11 | 256.03/173.00 | 256.03/190.00 | 22 | 18 | 31 |
| 280 | Proquinazid | 22.41 | 373.00/289.00 | 373.00/331.00 | 25 | 15 | 35 |
| 281 | Prosulfocarb | 20.02 | 252.14/91.00 | 252.14/128.00 | 20 | 15 | 29 |
| 282 | Pyraclostrobin | 17.84 | 388.11/194.00 | 388.11/164.00 | 15 | 23 | 30 |
| 283 | Pyraflufen-Ethyl | 17.00 | 413.10/339.10 | 413.10/289.10 | 20 | 30 | 45 |
| 284 | Pyrametostrobin | 15.83 | 382.10/163.00 | 382.10/194.00 | 25 | 10 | 20 |
| 285 | Pyraoxystrobin | 18.25 | 413.10/145.00 | 413.10/206.00 | 25 | 10 | 20 |
| 286 | Pyrazosulfuron-ethyl | 12.33 | 415.00/182.00 | 415.00/82.90 | 20 | 45 | 30 |
| 287 | Pyrethrins I | 22.25 | 329.10/133.10 | 329.10/161.10 | 15 | 10 | 25 |
| 288 | Pyribenzoxim | 21.53 | 610.28/180.03 | 610.28/413.08 | 12 | 10 | 20 |
| 289 | Pyridaben | 22.80 | 365.15/147.10 | 365.15/309.10 | 22 | 15 | 28 |
| 290 | Pyridalyl | 23.95 | 492.09/183.03 | 492.09/164.09 | 15 | 30 | 10 |
| 291 | Pyridaphenthion | 12.36 | 341.00/92.00 | 341.00/189.00 | 35 | 25 | 25 |
| 292 | Pyriftalid | 9.35 | 319.11/139.05 | 319.11/179.10 | 30 | 30 | 15 |
| 293 | Pyrimethanil | 9.47 | 200.12/107.00 | 200.12/82.00 | 24 | 24 | 51 |
| 294 | Pyriproxyfen | 21.37 | 322.20/95.90 | 322.20/184.90 | 15 | 20 | 15 |
| 295 | Pyrisoxazole | 10.14 | 289.00/120 | 289.00/151.00 | 23 | 12 | 30 |
| 296 | Quinalphos | 15.25 | 299.00/96.90 | 299.00/162.90 | 30 | 24 | 25 |
| 297 | Quizalofop-ethyl | 20.60 | 373.00/91.10 | 373.00/299.10 | 30 | 20 | 35 |
| 298 | Rotenone | 14.71 | 395.15/213.10 | 395.15/192.10 | 24 | 25 | 37 |
| 299 | Saflufenacil | 10.03 | 518.00/349.03 | 518.00/501.00 | 31 | 9 | 30 |
| 300 | Sedaxane | 12.27 | 332.10/159.10 | 332.10/292.20 | 17 | 15 | 40 |
| 301 | Sethoxydim | 21.06 | 328.19/178.00 | 328.19/282.00 | 20 | 10 | 34 |
| 302 | Silthiofam | 15.40 | 268.10/139.00 | 268.10/252.10 | 19 | 7 | 20 |
| 303 | Simazine | 5.69 | 202.08/132.00 | 202.08/124.00 | 18 | 18 | 40 |
| 304 | Simetryn | 6.90 | 214.00/95.90 | 214.00/124.00 | 25 | 20 | 50 |
| 305 | Spinetoram (J) | 20.32 | 748.53/98.07 | 748.53/142.16 | 64 | 30 | 35 |
| 306 | Spinetoram (L) | 21.08 | 760.53/98.07 | 760.53/142.16 | 66 | 30 | 35 |
| 307 | Spinosad A | 18.47 | 732.30/98.10 | 732.30/142.10 | 70 | 30 | 30 |
| 308 | Spinosad D | 20.02 | 746.30/98.10 | 746.30/142.10 | 70 | 31 | 51 |
| 309 | Spirodiclofen | 22.56 | 411.11/313.10 | 411.11/71.10 | 10 | 15 | 31 |
| 310 | Spirotetramat | 13.60 | 374.30/302.23 | 374.30/216.09 | 16 | 32 | 24 |
| 311 | Spirotetramat enol | 6.86 | 302.00/270.00 | 302.00/216.00 | 20 | 25 | 45 |
| 312 | Spirotetramat ketohydroxy | 8.43 | 318.00/214.00 | 318.00/268.00 | 25 | 20 | 18 |
| 313 | Spirotetramat monohydroxy | 5.52 | 304.00/119.00 | 304.00/254.00 | 35 | 15 | 30 |
| 314 | Spirotetramat-enol-glucoside | 3.64 | 464.10/302.20 | 464.10/216.10 | 12 | 45 | 10 |
| 315 | Sulfentrazone | 5.96 | 404.00/307.00 | 404.00/387.00 | 26 | 12 | 10 |
| 316 | Sulfotep | 16.25 | 323.03/171.00 | 323.03/153.00 | 18 | 18 | 28 |
| 317 | Sulfoxaflor | 4.07 | 278.03/153.87 | 278.03/173.89 | 30 | 10 | 25 |
| 318 | Tebuconazole | 15.91 | 308.15/70.10 | 308.15/125.00 | 22 | 40 | 40 |
| 319 | Tebufenozide | 15.48 | 353.22/133.00 | 353.22/297.10 | 20 | 12 | 30 |
| 320 | Tebuthiuron | 5.93 | 229.11/172.00 | 229.11/116.00 | 18 | 26 | 30 |
| 321 | Teflubenzuron | 20.96 | 381.00/158.00 | 381.00/141.00 | 15 | 35 | 30 |
| 322 | Terbufos sulfone | 9.88 | 321.04/171.00 | 321.04/143.00 | 12 | 22 | 20 |
| 323 | Terbufos sulfoxide | 9.87 | 305.05/97.00 | 305.05/187.00 | 40 | 15 | 20 |
| 324 | Terbuthylazine | 10.48 | 230.12/174.00 | 230.12/96.00 | 18 | 25 | 34 |
| 325 | Tetraconazole | 13.44 | 372.03/159.00 | 372.03/70.00 | 25 | 22 | 41 |
| 326 | Thiabendazole | 4.28 | 202.04/175.00 | 202.04/131.00 | 25 | 30 | 51 |
| 327 | Thiacloprid | 4.21 | 253.03/126.00 | 253.03/186.00 | 20 | 20 | 32 |
| 328 | Thiamethoxam | 3.48 | 292.03/211.07 | 292.03/132.00 | 18 | 20 | 30 |
| 329 | Thidiazuron | 5.51 | 221.00/102.00 | 221.00/128.00 | 15 | 15 | 30 |
| 330 | Thifensulfuron methyl | 5.13 | 388.00/167.00 | 388.00/56.00 | 30 | 15 | 20 |
| 331 | Thifluzamide | 14.42 | 528.80/148.00 | 528.80/168.00 | 42 | 28 | 50 |
| 332 | Thiophanate-methyl | 5.42 | 343.00/151.00 | 343.00/93.00 | 18 | 45 | 25 |
| 333 | Tolclofos-methyl | 17.52 | 300.93/124.91 | 300.93/268.94 | 15 | 15 | 15 |
| 334 | Tolfenpyrad | 21.23 | 384.15/197.00 | 384.15/145.00 | 25 | 28 | 54 |
| 335 | Tralkoxydim | 21.77 | 330.21/138.10 | 330.21/284.10 | 18 | 15 | 31 |
| 336 | Triadimefon | 11.86 | 294.10/69.00 | 294.10/197.00 | 20 | 20 | 31 |
| 337 | Triadimenol | 12.30 | 296.12/70.10 | 296.12/99.00 | 10 | 15 | 20 |
| 338 | Triallate | 21.64 | 304.00/86.00 | 304.00/142.90 | 15 | 25 | 10 |
| 339 | Triasulfuron | 5.22 | 402.06/167.00 | 402.06/141.00 | 17 | 20 | 30 |
| 340 | Triazophos | 12.57 | 314.10/118.90 | 314.10/161.90 | 30 | 20 | 15 |
| 341 | Trichlorfon | 4.01 | 256.90/109.00 | 256.90/79.00 | 15 | 30 | 35 |
| 342 | Trifloxystrobin | 20.07 | 409.14/186.00 | 409.14/145.00 | 14 | 40 | 25 |
| 343 | Triflumizole | 19.85 | 346.10/278.00 | 346.10/73.00 | 10 | 15 | 15 |
| 344 | Triflumuron | 17.98 | 359.04/156.00 | 359.04/139.00 | 16 | 35 | 30 |
| 345 | Triflusulfuron-methyl | 11.73 | 493.20/264.10 | 493.20/96.00 | 20 | 50 | 30 |
| 346 | Triticonazole | 13.28 | 318.10/70.10 | 318.10/124.90 | 16 | 35 | 30 |
| 347 | Tritosulfuron | 8.33 | 446.00/195.00 | 446.00/145.00 | 35 | 18 | 34 |
| 348 | Vamidothion | 3.91 | 288.05/146.00 | 288.05/118.00 | 15 | 18 | 30 |
| 349 | Zoxamide | 16.43 | 336.03/187.00 | 336.03/204.00 | 25 | 25 | 30 |
| 350 | Benazolin-ethyl | 9.61 | 272.00/170.00 | 272.00/198.00 | 28 | 14 | 20 |
| 351 | Cyazofamid metabolite CCIM | 11.70 | 218.00/35.00 | 218.00/37.00 | 27 | 28 | 25 |
| 352 | Fensulfothion sulfone | 8.19 | 325.00/269.00 | 325.00/297.00 | 15 | 10 | 15 |
| 353 | Mesosulfuron methyl | 7.24 | 504.21/181.95 | 504.21/82.69 | 24 | 54 | 2 |
| 354 | Pirimicarb-desmethyl-formamido | 5.54 | 253.30/72.00 | 253.30/225.00 | 16 | 9 | 32 |
| 355 | Prochloraz metabolite BTS44596 | 16.29 | 353.00/70.00 | 353.00/308.00 | 26 | 12 | 32 |
| 356 | Propisochlor | 16.36 | 284.20/73.00 | 284.20/224.10 | 11 | 8 | 30 |
| 357 | Pyrethrins II | 20.16 | 373.20/133.00 | 373.20/161.00 | 16 | 10 | 30 |
| 358 | Pyrimorph | 16.98 | 385.10/57.00 | 385.10/242.00 | 32 | 27 | 40 |
| 359 | Spiromesifen | 22.16 | 388.00/255.00 | 388.00/273.00 | 26 | 16 | 10 |
| 360 | terbufos | 20.92 | 289.10/57.00 | 289.10/103.10 | 15 | 5 | 10 |
| 361 | Triflumizole metabolite FM-6-1 | 4.87 | 295.20/43.00 | 295.20/73.00 | 24 | 18 | 50 |
| 362 | Uniconazole | 14.13 | 292.20/70.00 | 292.20/125.00 | 24 | 28 | 30 |
| 363 | Xylazine | 10.63 | 221.10/89.70 | 221.10/163.70 | 40 | 19 | 19 |
| 364 | Pindolol | 6.63 | 249.10/115.80 | 249.10/171.80 | 50 | 14 | 14 |
| 365 | Alprenolol | 17.65 | 250.10/115.80 | 250.10/71.80 | 50 | 17 | 15 |
| 366 | Propranolol | 17.36 | 260.20/115.80 | 260.20/182.70 | 50 | 16 | 15 |
| 367 | Oxprenolol | 15.38 | 266.30/115.80 | 266.30/71.50 | 40 | 15 | 15 |
| 368 | Acebutolol | 12.57 | 337.20/115.80 | 337.20/319.10 | 18 | 14 | 50 |
| 369 | Metoprolol | 12.59 | 268.10/115.80 | 268.10/190.70 | 45 | 14 | 14 |
| 370 | Levobunolol | 13.45 | 292.10/235.90 | 292.10/200.90 | 19 | 19 | 45 |
| 371 | Carazolol | 15.36 | 299.20/115.80 | 299.20/221.90 | 50 | 16 | 17 |
| 372 | Timolol | 12.01 | 317.00/261.10 | 317.00/73.50 | 13 | 19 | 55 |
| 373 | Betaxolol | 18.00 | 308.10/115.80 | 308.10/207.20 | 45 | 20 | 17 |
| 374 | Droperidol | 17.01 | 380.10/164.80 | 380.10/193.70 | 24 | 13 | 50 |
| 375 | Carvedilol | 19.26 | 407.00/99.80 | 407.00/223.70 | 26 | 19 | 45 |
| 376 | Nebivolol | 20.77 | 406.00/150.80 | 406.00/122.80 | 35 | 30 | 30 |
| 377 | Acepromazine | 19.40 | 327.10/85.80 | 327.10/253.80 | 17 | 21 | 50 |
| 378 | Chlorpromazine | 21.25 | 319.00/85.80 | 319.00/245.90 | 15 | 19 | 45 |
| 379 | Nadolol | 7.50 | 310.00/254.00 | 310.00/235.90 | 20 | 25 | 20 |
| 380 | Estazolam | 20.12 | 294.90/266.90 | 294.90/191.60 | 35 | 20 | 25 |
| 381 | Penbutolol | 13.42 | 292.20/236.00 | 292.20/73.70 | 40 | 18 | 13 |
| 382 | Demoxepam | 20.23 | 286.90/269.00 | 286.90/240.90 | 35 | 20 | 15 |
| 383 | Diazapam | 21.91 | 284.90/153.60 | 284.90/192.70 | 35 | 22 | 25 |
| 384 | Nitrazepam | 19.20 | 282.10/207.30 | 282.10/235.90 | 55 | 28 | 20 |
| 385 | Acetylkitasamycin | 22.49 | 856.50/109.10 | 856.50/174.20 | 10 | 38 | 30 |
| 386 | Narasin | 24.73 | 787.60/431.30 | 787.60/279.10 | 40 | 45 | 20 |
| 387 | Salinomycin | 25.10 | 733.60/431.20 | 733.60/531.10 | 40 | 35 | 30 |
| 388 | Monensin | 24.99 | 693.30/675.10 | 693.30/461.10 | 30 | 40 | 35 |
| 389 | lufutong | 17.20 | 416.10/397.80 | 416.10/137.70 | 30 | 15 | 20 |
| 390 | Sulfanitran | 19.46 | 336.00/294.00 | 336.00/156.00 | 20 | 10 | 10 |
| 391 | Guanabenz | 21.92 | 334.20/137.80 | 334.20/154.80 | 20 | 18 | 30 |
| 392 | Trimethoprim | 7.11 | 291.00/261.00 | 291.00/230.00 | 20 | 15 | 25 |
| 393 | Diaveridine | 5.80 | 261.20/122.80 | 261.20/244.90 | 20 | 25 | 30 |
| 394 | Ethopabate | 16.37 | 238.10/205.80 | 238.10/163.70 | 30 | 20 | 10 |
| 395 | Sulfachlorpyridazine | 9.35 | 285.10/155.90 | 285.10/108.10 | 20 | 12 | 12 |
| 396 | Sulfamonomethoxine | 7.94 | 281.00/156.00 | 281.00/126.00 | 20 | 16 | 16 |
| 397 | Sulfisomidine | 7.31 | 279.00/186.00 | 279.00/124.00 | 16 | 19 | 20 |
| 398 | Sulfabenzamine | 13.62 | 276.90/156.00 | 276.90/108.00 | 20 | 20 | 15 |
| 399 | Sulfamethizole | 7.53 | 271.00/156.00 | 271.00/108.00 | 20 | 14 | 14 |
| 400 | Sulfamoxole | 12.67 | 268.10/156.00 | 268.10/113.10 | 20 | 14 | 16 |
| 401 | Sulfisoxazole | 12.65 | 268.00/156.00 | 268.00/113.00 | 20 | 16 | 14 |
| 402 | Sulfamerazine | 5.98 | 265.10/156.00 | 265.10/172.00 | 20 | 15 | 15 |
| 403 | Sulfathiazole | 5.48 | 256.10/156.00 | 256.10/108.00 | 20 | 20 | 20 |
| 404 | Sulfamethoxazole | 10.70 | 254.00/156.00 | 254.00/108.00 | 20 | 14 | 14 |
| 405 | Sulfapyridine | 5.72 | 250.00/156.00 | 250.00/184.00 | 20 | 16 | 16 |
| 406 | Sulfaguanidine | 3.64 | 215.00/156.00 | 215.00/108.00 | 20 | 20 | 15 |
| 407 | Sulfaquinoxaline | 16.10 | 301.10/156.00 | 301.10/107.90 | 20 | 15 | 15 |
| 408 | Sulfadimethoxine | 11.51 | 311.00/108.00 | - | 20 | 20 | 15 |
| 409 | Sulfadoxine | 11.53 | 311.00/156.00 | - | 20 | 15 | 15 |
| 410 | Sulfadimidine | 7.32 | 279.00/124.00 | 279.00/186.00 | 20 | 19 | 16 |
| 411 | Ronidazole | 4.54 | 201.00/140.00 | 201.00/55.00 | 20 | 22 | 10 |
| 412 | Metronidazole-hydroxy | 2.59 | 188.00/126.00 | 188.00/144.00 | 25 | 14 | 10 |
| 413 | Metronidazole | 3.96 | 172.00/128.00 | 172.00/82.00 | 20 | 20 | 14 |
| 414 | Ipronidazole | 10.66 | 170.00/109.00 | 170.00/124.00 | 20 | 14 | 40 |
| 415 | 5-nitrobenzimidazole | 5.50 | 164.00/118.00 | 164.00/91.00 | 20 | 18 | 15 |
| 416 | 5-chloro-1-methyl-4-nitroimidazole | 5.70 | 162.00/116.00 | 162.00/81.00 | 15 | 20 | 15 |
| 417 | Dimetridazole-2-hydroxy | 3.56 | 158.00/55.00 | 158.00/140.00 | 30 | 16 | 10 |
| 418 | Dimetridazole | 5.50 | 164.00/118.00 | 164.00/91.00 | 20 | 18 | 15 |
| 419 | 4-nitroimidazole | 1.58 | 114.00/68.00 | 114.00/84.00 | 15 | 14 | 10 |
| 420 | Enoxacin | 7.59 | 321.15/302.96 | 321.15/205.76 | 16 | 26 | 28 |
| 421 | Kitasamycin | 21.35 | 786.50/109.10 | 786.50/174.20 | 10 | 38 | 30 |
| 422 | Flumequine | 18.67 | 262.18/261.80 | 262.18/243.70 | 20 | 30 | 15 |
| 423 | Roxithromycin | 21.69 | 837.50/158.20 | 837.50/679.70 | 10 | 35 | 20 |
| 424 | Virginiamycin S1 | 22.36 | 824.30/205.00 | 824.30/190.00 | 50 | 35 | 40 |
| 425 | Rifampicin | 22.55 | 823.40/399.20 | 823.40/791.60 | 25 | 18 | 10 |
| 426 | Midecamycin | 21.34 | 814.50/109.00 | 814.50/174.00 | 20 | 45 | 35 |
| 427 | Desmycosin | 19.15 | 772.40/174.10 | 772.40/98.20 | 10 | 40 | 28 |
| 428 | Oleandomycin | 18.14 | 684.00/158.20 | 684.00544.40 | 28 | 18 | 10 |
| 429 | Virginiamycin M1 | 21.44 | 526.20/355.10 | 526.20/508.10 | 30 | 17 | 14 |
| 430 | Lincomycin | 6.53 | 407.20/126.20 | 407.20/359.30 | 28 | 18 | 10 |
| 431 | Tylosin tartrate | 20.27 | 916.50/174.20 | 916.50/101.20 | 10 | 45 | 35 |
| 432 | Acetanilide | 7.89 | 136.10/94.00 | 136.10/77.00 | 20 | 25 | 15 |
| 433 | Benzocaine | 15.23 | 166.10/138.00 | 166.10/94.00 | 20 | 20 | 15 |
| 434 | Detomidine | 13.82 | 187.10/81.00 | 187.10/54.00 | 20 | 32 | 22 |
| 435 | Levamisole | 5.63 | 205.10/178.00 | 205.10/91.00 | 20 | 30 | 20 |
| 436 | Clonidine | 5.40 | 230.00/44.10 | 230.00/167.00 | 22 | 30 | 20 |
| 437 | Lidocaine | 7.37 | 235.20/86.00 | 235.20/58.00 | 20 | 32 | 18 |
| 438 | flurbiprofen | 23.52 | 245.10/115.00 | 245.10/87.00 | 20 | 20 | 12 |
| 439 | Fenbufen | 21.92 | 255.10/237.00 | 255.10/181.00 | 20 | 25 | 10 |
| 440 | Diphenhydramine | 18.00 | 256.20/167.00 | 256.20/152.00 | 20 | 35 | 12 |
| 441 | Clenproperol | 8.06 | 263.10/245.00 | 263.10/203.00 | 20 | 18 | 10 |
| 442 | Antazoline | 17.16 | 266.20/196.00 | 266.20/91.00 | 20 | 25 | 15 |
| 443 | Chlormezanone | 16.68 | 274.00/154.00 | 274.00/209.00 | 20 | 15 | 15 |
| 444 | Clotrimazole | 21.29 | 277.20/242.00 | 278.20/166.00 | 20 | 16 | 18 |
| 445 | Doxepin | 18.57 | 280.20/107.00 | 280.20/235.00 | 20 | 22 | 18 |
| 446 | Cyproheptadine | 20.00 | 288.20/96.00 | 288.20/191.00 | 20 | 25 | 28 |
| 447 | Clencyclohexerol | 21.99 | 319.00/300.90 | 319.00/202.90 | 34 | 22 | 16 |
| 448 | clenpenterol | 21.99 | 319.00/300.90 | 319.00/202.90 | 34 | 22 | 16 |
| 449 | Anastrozole | 18.74 | 294.20/225.2 | 294.20/142.00 | 20 | 38 | 20 |
| 450 | Diclofenac | 22.87 | 297.00/216.00 | 297.00/251.00 | 20 | 18 | 12 |
| 451 | Isoxsuprine | 14.76 | 302.20/284.20 | 302.20/150.00 | 15 | 20 | 20 |
| 452 | Fluconazole | 11.70 | 307.10/238.00 | 307.10/220.00 | 20 | 18 | 16 |
| 453 | Bifonazole | 21.53 | 311.20/243.00 | 311.20/165.00 | 20 | 35 | 30 |
| 454 | Ketotifen | 16.24 | 310.10/96.00 | 310.10/82.00 | 20 | 32 | 20 |
| 455 | Kresoxim-methyl | 14.56 | 314.00/116.00 | 314.00/131.00 | 30 | 20 | 30 |
| 456 | Clomipramine | 21.51 | 315.20/86.00 | 315.20/58.00 | 20 | 28 | 15 |
| 457 | Chlorprothixene | 21.43 | 316.10/271.00 | 316.10/86.00 | 20 | 15 | 20 |
| 458 | Gliclazide | 21.74 | 324.10/127.00 | 324.10/153.00 | 20 | 20 | 18 |
| 459 | Citalopram | 18.32 | 325.20/109.00 | 325.20/262.20 | 20 | 22 | 18 |
| 460 | Danazol | 23.21 | 338.20/148.00 | 338.20/120.00 | 25 | 30 | 20 |
| 461 | Griseofulvin | 20.87 | 353.10/165.00 | 353.10/215.00 | 20 | 18 | 18 |
| 462 | bisacodyl | 20.66 | 362.10/184.00 | 362.10/226.00 | 28 | 18 | 20 |
| 463 | hydrocortisone | 18.78 | 363.20/121.00 | 363.20/327.20 | 22 | 15 | 20 |
| 464 | indapamide | 18.43 | 366.10/130.00 | 366.10/91.00 | 18 | 18 | 20 |
| 465 | Bumetanide | 21.86 | 365.10/240.10 | 365.10/184.00 | 15 | 20 | 20 |
| 466 | Bromhexine | 19.17 | 375.00/114.00 | 375.00/261.80 | 20 | 18 | 28 |
| 467 | Doxapram | 15.51 | 379.20/292.00 | 379.20/97.00 | 20 | 30 | 20 |
| 468 | Econazol | 22.34 | 381.00/125.00 | 381.00/255.00 | 20 | 24 | 20 |
| 469 | Betamethasone | 20.24 | 393.20/373.20 | 393.20/355.10 | 20 | 28 | 12 |
| 470 | Benzthiazide | 13.10 | 432.00/91.00 | 432.00/65.00 | 20 | 70 | 20 |
| 471 | Glibenclamide | 22.75 | 494.20/169.00 | 494.20/369.00 | 30 | 15 | 20 |
| 472 | Glimepiride | 23.05 | 491.20/126.00 | 491.20/352.00 | 25 | 12 | 20 |
| 473 | glibenclamide | 22.75 | 494.20/169.00 | 494.20/369.00 | 30 | 15 | 20 |
| 474 | Dipyridamole | 19.27 | 505.30/385.20 | 505.30/429.20 | 42 | 42 | 20 |
| 475 | Thiabendazole | 4.28 | 202.04/175.00 | 202.04/131.00 | 25 | 30 | 51 |
| 476 | Levamisole Hydrochloride | 5.65 | 205.10/178.20 | 205.10/ 91.20 | 20 | 30 | 20 |
| 477 | 5-Hydroxythiabendazole | 5.66 | 218.00/147.20 | 218.0/191.10 | 30 | 25 | 20 |
| 478 | 4-Formylaminoantipyrine | 6.51 | 232.10/104.20 | 232.10/214.20 | 22 | 12 | 20 |
| 479 | 4-acetylaminoantipyrine | 20.83 | 246.10/228.20 | 246.10/104.20 | 12 | 25 | 20 |
| 480 | sudan 1 | 24.18 | 249.10/93.10 | 249.10/232.20 | 20 | 12 | 20 |
| 481 | Oxibendazole | 15.78 | 250.10/148.20 | 250.10/218.10 | 35 | 18 | 20 |
| 482 | Albendazole | 18.60 | 266.00/191.00 | 266.00/234.00 | 32 | 20 | 20 |
| 483 | Disperse yellow 3 | 22.82 | 270.10/107.20 | 270.10/150.20 | 25 | 15 | 20 |
| 484 | Tolbutamide | 20.40 | 271.10/91.10 | 271.10/172.20 | 30 | 12 | 20 |
| 485 | Sudan 2 | 24.91 | 277.10/121.20 | 277.10/156.10 | 20 | 15 | 20 |
| 486 | Albendazole sulfoxide | 11.61 | 282.10/240.00 | 282.10/159.00 | 12 | 38 | 20 |
| 487 | N-acetyl dapsone | 12.26 | 291.20/108.20 | 291.20/198.20 | 30 | 25 | 14 |
| 488 | fenthion sulfoxide | 19.25 | 295.00/109.00 | 295.00/280.00 | 30 | 20 | 45 |
| 489 | 5-Hydroxymebendazole | 14.37 | 298.10/160.20 | 298.10/266.20 | 35 | 35 | 20 |
| 490 | Cambendazole | 15.43 | 303.00/217.20 | 303.00/243.20 | 28 | 30 | 20 |
| 491 | Oxfendazole | 15.84 | 316.00/191.20 | 316.00/284.00 | 20 | 18 | 20 |
| 492 | Fenbendazole sulfone | 17.60 | 332.00/300.20 | 332.00/159.00 | 20 | 35 | 22 |
| 493 | Robenidine Hydrochloride | 21.91 | 334.10/155.20 | 334.10/138.20 | 18 | 25 | 20 |
| 494 | Sudan blue 2 | 25.02 | 351.20/251.20 | 351.20/294.20 | 30 | 18 | 20 |
| 495 | Acid yellow 36 | 25.02 | 352.20/252.10 | 352.20/295.20 | 30 | 20 | 30 |
| 496 | Indometacin | 22.91 | 358.10/139.10 | 358.10/174.20 | 18 | 12 | 20 |
| 497 | Triclabendazole | 23.37 | 359.00/274.00 | 359.00/309.00 | 36 | 30 | 20 |
| 498 | Glipizide | 21.10 | 446.20/321.20 | 446.20/167.00 | 20 | 30 | 15 |
| 499 | Febantel | 22.82 | 447.10/415.20 | 447.10/383.10 | 20 | 32 | 18 |
| 500 | glibenclamide | 22.75 | 494.20/169.00 | 494.20/369.00 | 30 | 15 | 20 |
| 501 | Valnemulin hydrochloride | 21.81 | 565.40/263.20 | 565.40/164.20 | 16 | 30 | 20 |
| 502 | Phenylethanolamine A | 18.38 | 345.20/327.20 | 345.20/150.20 | 12 | 20 | 20 |
| 503 | Cimaterol | 4.23 | 220.10/160.10 | 220.10/143.00 | 30 | 24 | 15 |
| 504 | Terbutaline | 4.32 | 226.10/152.00 | 226.10/170.00 | 16 | 12 | 30 |
| 505 | Salbutamol | 4.54 | 240.20/148.10 | 240.20/222.10 | 20 | 12 | 30 |
| 506 | Procaterol | 5.43 | 291.20/273.20 | 291.20/231.10 | 12 | 18 | 20 |
| 507 | Cimbuterol | 5.51 | 234.20/160.10 | 234.20/217.00 | 30 | 15 | 10 |
| 508 | clonidine | 5.43 | 229.80/212.80 | 229.80/43.90 | 24 | 24 | 22 |
| 509 | Fenoterol | 5.76 | 304.20/135.10 | 304.20/107.00 | 20 | 18 | 30 |
| 510 | Clenproperol | 8.09 | 291.10/273.00 | 291.10/203.00 | 20 | 10 | 15 |
| 511 | Clorprenaline | 8.84 | 214.00/154.00 | 214.00/118.00 | 25 | 22 | 18 |
| 512 | Ractopamine | 9.42 | 302.20/164.10 | 302.20/284.20 | 15 | 12 | 30 |
| 513 | lsoxsuprine | 14.77 | 302.20/150.10 | 302.20/284.20 | 20 | 25 | 30 |
| 514 | Clenbuterol | 11.63 | 277.10/203.00 | 277.10/168.10 | 30 | 28 | 46 |
| 515 | Tulobuterol | 12.49 | 228.10/154.00 | 228.10/172.00 | 30 | 15 | 12 |
| 516 | Formoterol | 12.89 | 345.10/149.00 | 345.10/327.10 | 14 | 18 | 20 |
| 517 | Clencyclohexerol | 21.99 | 320.93/300.19 | 320.93/205.29 | 10 | 16 | 34 |
| 518 | Brombuterol | 13.96 | 365.10/290.90 | 365.10/217.10 | 30 | 20 | 25 |
| 519 | Clenpenterol | 8.09 | 291.10/273.00 | 291.10/203.00 | 20 | 10 | 15 |
| 520 | Bambuterol | 15.78 | 368.10/294.30 | 368.10/312.30 | 44 | 28 | 16 |
| 521 | clenhexerol | 18.84 | 304.95/287.25 | 304.95/132.45 | 34 | 10 | 26 |
| 522 | cyproheptadine | 19.22 | 288.00/96.00 | 288.20/191.00 | 24 | 28 | 29 |
| 523 | Salmeterol | 21.46 | 416.16/398.26 | 416.16/380.27 | 12 | 16 | 50 |
| 524 | Amiloride | 4.67 | 229.90/171.00 | 229.90/189.00 | 14 | 16 | 25 |
| 525 | Trenbolone | 20.40 | 271.00/199.00 | 271.00/165.30 | 22 | 44 | 20 |
| 526 | Levonorgestrel | 23.10 | 313.10/108.90 | 313.10/254.40 | 30 | 30 | 25 |
| 527 | Megestrol acetate | 23.09 | 385.10/325.10 | 385.10/267.10 | 16 | 16 | 30 |
| 528 | Androstendione | 21.91 | 287.00/97.20 | 287.00/109.20 | 20 | 24 | 20 |
| 529 | Beclomethasone | 20.61 | 409.50/373.40 | 409.50/121.20 | 8 | 42 | 20 |
| 530 | Danazol | 23.09 | 338.70/148.00 | 338.70/120.00 | 25 | 29 | 20 |
| 531 | Methylandrostendiol | 20.05 | 287.40/269.10 | 287.40/159.10 | 20 | 11 | 21 |
| 532 | Betamethasone | 20.14 | 393.20/373.20 | 393.20/355.10 | 8 | 12 | 20 |
| 533 | Corticosterone | 20.53 | 347.20/329.50 | 347.20/121.00 | 15 | 20 | 20 |
| 534 | testosterone | 21.78 | 289.00/109.00 | 289.00/ 97.00 | 20 | 22 | 35 |
| 535 | indapamide | 18.43 | 366.10/130.00 | 366.10/91.00 | 18 | 18 | 20 |
| 536 | Canrenone | 22.11 | 341.00/107.00 | 341.00/187.00 | 25 | 24 | 22 |
| 537 | Minocycline | 23.63 | 305.60/105.20 | 305.60/269.40 | 20 | 14 | 20 |
| 538 | Triamterene | 8.02 | 253.90/237.00 | 253.90/104.00 | 23 | 30 | 50 |
| 539 | Nandrolone Phenylpropionate | 24.43 | 407.00/105.00 | 407.00/257.00 | 28 | 15 | 35 |
| 540 | Testosterone propionate | 24.08 | 345.00/97.00 | 345.00/109.00 | 35 | 20 | 22 |
| 541 | Nandrolone 17-propionate | 23.87 | 331.00/257.00 | 331.00/275.00 | 16 | 16 | 30 |
| 542 | Methyltestosterone | 22.20 | 303.00/109.00 | 303.00/97.00 | 35 | 25 | 27 |
| 543 | Epiandrosterone | 16.45 | 291.00/273.00 | 291.00/255.10 | 15 | 10 | 5 |
| 544 | Testosterone | 21.78 | 289.00/109.00 | 289.00/ 97.00 | 20 | 22 | 35 |
| 545 | Boldenone | 20.97 | 287.00/121.00 | 287.00/135.00 | 22 | 15 | 20 |
| 546 | Nandrolone | 21.14 | 275.10/109.00 | 275.10/257.00 | 28 | 15 | 35 |
| 547 | Medroxyprogesterone | 23.20 | 387.10/327.10 | 387.10/285.10 | 30 | 16 | 16 |
| 548 | Chlormadinone acetate | 23.13 | 405.10/345.10 | 405.10/309.10 | 28 | 16 | 12 |
| 549 | testosterone | 24.08 | 345.00/97.00 | 345.00/109.00 | 35 | 20 | 22 |
| 550 | Aspirin | 29.27 | 181.10/99.00 | 181.10/140.00 | 20 | 15 | 10 |
| 551 | Rafoxanide | 25.04 | 625.80/127.00 | 625.80/373.00 | 30 | 30 | 20 |
| 552 | doramection | 24.92 | 899.50/113.20 | 899.50/219.20 | 20 | 30 | 15 |

Table S7. Estimation of matrix effect, average recovery ratio (R, %) and relative standard deviation (RSD, %) at three different levels for serum (n = 3).

| Serial Number | Analytes | This work | | | 2 | | | 3 | | |
| --- | --- | --- | --- | --- | --- | --- | --- | --- | --- | --- |
|  |  | R (%) | RSD | MF | R (%) | RSD | MF | R (%) | RSD | MF |
| 1 | Acephate | 94.67 | 0.50 | 1.00 | 97.99 | 0.91 | 0.97 | 98.27 | 0.29 | 0.97 |
| 2 | Acetamiprid | 94.57 | 3.07 | 0.90 | 99.65 | 1.63 | 0.79 | 96.41 | 2.21 | 0.75 |
| 3 | Acetochlor | 89.47 | 0.59 | 1.11 | 91.61 | 2.89 | 1.23 | 92.67 | 1.49 | 1.26 |
| 4 | Alachlor | 93.18 | 2.95 | 1.01 | 95.26 | 1.71 | 1.00 | 95.18 | 2.12 | 0.99 |
| 5 | Albendazole | 89.22 | 3.05 | 1.16 | 88.64 | 2.62 | 1.40 | 94.22 | 0.74 | 1.40 |
| 6 | Aldicarb | 93.71 | 8.78 | 1.03 | 95.19 | 18.91 | 1.13 | 115.42 | 13.34 | 1.08 |
| 7 | Aldicarb sulfone | 96.43 | 1.37 | 0.96 | 96.75 | 3.24 | 0.89 | 96.96 | 0.54 | 0.87 |
| 8 | Aldicarb sulfoxide | 97.53 | 2.75 | 0.91 | 98.97 | 2.49 | 0.79 | 96.93 | 0.97 | 0.79 |
| 9 | Ametoctradin | 72.57 | 1.71 | 1.33 | 82.81 | 4.16 | 1.54 | 79.56 | 1.10 | 1.65 |
| 10 | Amidosulfuron | 89.73 | 2.68 | 0.93 | 94.23 | 2.84 | 1.03 | 96.08 | 2.11 | 1.06 |
| 11 | Amisulbrom | 98.82 | 5.91 | 0.87 | 91.93 | 2.40 | 1.04 | 95.55 | 1.51 | 1.01 |
| 12 | Anilofos | 96.59 | 1.24 | 1.03 | 95.39 | 3.09 | 1.06 | 93.15 | 1.04 | 1.08 |
| 13 | Atrazine | 93.97 | 1.13 | 1.09 | 87.55 | 2.84 | 1.29 | 92.78 | 1.44 | 1.29 |
| 14 | Avermectin B1a | 88.31 | 3.89 | 1.01 | 82.60 | 15.59 | 1.23 | 87.75 | 4.62 | 1.17 |
| 15 | Azinphos-methyl | 96.29 | 2.97 | 1.02 | 96.18 | 1.13 | 1.04 | 102.02 | 3.85 | 0.98 |
| 16 | Azoxystrobin | 93.62 | 0.83 | 1.03 | 93.66 | 3.27 | 1.08 | 98.72 | 0.82 | 1.02 |
| 17 | Benalaxyl | 90.93 | 0.99 | 1.11 | 90.61 | 1.60 | 1.23 | 91.83 | 0.93 | 1.25 |
| 18 | Bendiocarb | 96.50 | 1.61 | 1.07 | 93.69 | 1.95 | 1.18 | 94.23 | 0.63 | 1.21 |
| 19 | Bensulfuron-methyl | 89.92 | 4.41 | 0.90 | 94.88 | 2.15 | 0.92 | 99.37 | 2.61 | 0.87 |
| 20 | Benzovindiflupyr | 89.24 | 1.10 | 1.22 | 88.03 | 4.87 | 1.43 | 95.83 | 1.43 | 1.41 |
| 21 | Benzoximate | 91.51 | 2.22 | 1.06 | 87.00 | 3.52 | 1.17 | 90.43 | 1.13 | 1.13 |
| 22 | Bifenox | 96.81 | 4.97 | 1.13 | 91.87 | 5.88 | 1.27 | 90.98 | 1.41 | 1.30 |
| 23 | Bifenthrin | 96.29 | 10.17 | 1.08 | 83.30 | 3.69 | 1.00 | 80.98 | 3.72 | 1.06 |
| 24 | Bioresmethrin | 93.58 | 1.13 | 1.03 | 88.14 | 1.95 | 1.02 | 86.11 | 1.91 | 1.04 |
| 25 | Bitertanol | 89.15 | 2.54 | 0.90 | 97.80 | 3.14 | 0.84 | 95.01 | 3.18 | 0.77 |
| 26 | Boscalid | 91.65 | 4.56 | 1.06 | 91.45 | 1.39 | 1.21 | 94.62 | 1.89 | 1.24 |
| 27 | Bromuconazole | 75.57 | 0.83 | 1.11 | 87.39 | 0.77 | 1.24 | 96.94 | 2.22 | 1.18 |
| 28 | Bupirimate | 85.06 | 2.84 | 1.20 | 86.18 | 4.03 | 1.34 | 90.90 | 2.17 | 1.32 |
| 29 | Buprofenzin | 90.06 | 1.47 | 1.22 | 60.87 | 84.17 | 1.37 | 88.59 | 1.08 | 1.45 |
| 30 | Butachlor | 95.49 | 1.26 | 1.07 | 90.02 | 3.53 | 1.28 | 88.98 | 0.45 | 1.33 |
| 31 | Butralin | 94.44 | 1.60 | 1.05 | 90.23 | 1.21 | 1.17 | 89.90 | 2.13 | 1.19 |
| 32 | Cadusafos | 90.19 | 0.88 | 1.06 | 92.66 | 2.20 | 1.10 | 90.97 | 3.35 | 1.11 |
| 33 | Carbaryl | 99.72 | 2.06 | 0.87 | 102.87 | 2.90 | 0.75 | 96.37 | 2.38 | 0.69 |
| 34 | Carbendazim | 92.19 | 2.27 | 1.49 | 94.43 | 3.34 | 1.45 | 96.55 | 0.76 | 1.52 |
| 35 | Carbofuran | 89.80 | 1.82 | 1.33 | 87.65 | 3.38 | 1.66 | 93.34 | 1.89 | 1.83 |
| 36 | Carbofuran-3-hydroxy | 92.08 | 3.05 | 1.02 | 96.42 | 3.95 | 0.96 | 96.89 | 2.44 | 0.95 |
| 37 | Carboxin | 88.59 | 6.49 | 1.07 | 90.19 | 2.11 | 1.25 | 94.48 | 0.67 | 1.24 |
| 38 | Carfentrazone-ethyl | 91.89 | 1.43 | 1.11 | 92.19 | 2.62 | 1.22 | 95.13 | 1.42 | 1.25 |
| 39 | Chlorantranilipole | 95.87 | 2.31 | 1.00 | 94.72 | 2.51 | 1.05 | 95.89 | 1.13 | 1.06 |
| 40 | Chlorbenzuron | 91.62 | 2.37 | 1.06 | 94.94 | 2.42 | 1.08 | 96.42 | 1.29 | 1.09 |
| 41 | Chlordimeform | 88.93 | 1.67 | 1.16 | 84.50 | 3.91 | 1.39 | 91.67 | 0.88 | 1.40 |
| 42 | Chlorfenvinphos | 91.47 | 1.78 | 1.20 | 88.73 | 3.51 | 1.47 | 89.54 | 1.06 | 1.57 |
| 43 | Chlorfluazuron | 91.62 | 1.49 | 1.07 | 91.04 | 3.32 | 1.11 | 88.66 | 5.03 | 1.14 |
| 44 | Chloridazon | 83.99 | 4.03 | 1.13 | 83.60 | 5.64 | 1.98 | 91.30 | 2.21 | 2.12 |
| 45 | Chlorimuron ethyl | 90.01 | 0.12 | 0.85 | 89.07 | 2.18 | 0.97 | 92.73 | 1.79 | 0.93 |
| 46 | Chlorpropham | 83.34 | 5.82 | 1.49 | 79.76 | 6.48 | 2.26 | 90.02 | 4.08 | 2.46 |
| 47 | Chlorpyrifos | 80.77 | 4.23 | 1.05 | 86.78 | 3.70 | 1.17 | 82.88 | 4.93 | 1.06 |
| 48 | Chlorpyriphos-methyl | 58.64 | 7.07 | 1.12 | 88.48 | 5.01 | 1.19 | 91.10 | 4.38 | 1.15 |
| 49 | Chlorsulfuron | 87.38 | 1.94 | 0.95 | 91.79 | 2.83 | 1.10 | 102.73 | 1.48 | 1.06 |
| 50 | Chlortoluron | 90.05 | 0.59 | 1.15 | 90.69 | 4.03 | 1.27 | 94.30 | 0.25 | 1.27 |
| 51 | Chromafenozide | 95.12 | 1.24 | 0.99 | 98.15 | 3.02 | 0.97 | 92.77 | 3.84 | 0.98 |
| 52 | Cinosulfuron | 91.41 | 0.28 | 1.04 | 92.15 | 1.29 | 1.09 | 97.04 | 2.44 | 1.10 |
| 53 | Clethodim | 90.26 | 4.69 | 0.79 | 92.67 | 4.38 | 0.86 | 97.98 | 0.71 | 0.84 |
| 54 | Clethodim sulfone | 90.01 | 1.93 | 0.86 | 93.19 | 1.01 | 0.91 | 92.44 | 1.10 | 0.95 |
| 55 | Clethodim sulfoxide | 76.74 | 0.80 | 0.89 | 89.44 | 2.79 | 0.96 | 88.80 | 1.55 | 0.95 |
| 56 | Clofentezine | 91.85 | 3.13 | 1.15 | 85.37 | 2.67 | 1.42 | 89.97 | 1.32 | 1.45 |
| 57 | Clomazone | 93.23 | 0.68 | 1.08 | 92.90 | 3.55 | 1.18 | 95.76 | 1.59 | 1.17 |
| 58 | Clothianidin | 91.55 | 6.82 | 1.12 | 107.43 | 3.01 | 0.66 | 102.83 | 3.75 | 1.00 |
| 59 | Coumaphos | 87.99 | 4.36 | 1.22 | 87.48 | 3.35 | 1.51 | 90.72 | 1.60 | 1.55 |
| 60 | Coumoxystrobin | 83.14 | 4.40 | 1.08 | 91.47 | 5.45 | 1.09 | 91.60 | 0.55 | 1.07 |
| 61 | Cyanazine | 96.07 | 1.12 | 0.98 | 97.83 | 2.76 | 1.06 | 94.00 | 1.20 | 1.10 |
| 62 | Cyantraniliprole | 95.92 | 1.38 | 1.03 | 92.50 | 1.08 | 1.12 | 95.96 | 2.14 | 1.05 |
| 63 | Cyazofamid | 93.35 | 2.30 | 0.97 | 93.46 | 1.75 | 1.00 | 95.65 | 3.04 | 0.97 |
| 64 | Cyclosulfamuron | 86.89 | 0.84 | 0.98 | 90.58 | 4.80 | 1.09 | 93.61 | 0.67 | 1.12 |
| 65 | Cycloxydim | 84.35 | 4.75 | 0.93 | 84.50 | 6.54 | 1.31 | 88.72 | 2.28 | 1.39 |
| 66 | Cyflufenamid | 90.78 | 0.65 | 1.15 | 92.83 | 2.92 | 1.18 | 93.90 | 1.90 | 1.19 |
| 67 | Cyflumetofen | 94.61 | 2.41 | 1.03 | 93.86 | 2.24 | 1.06 | 93.08 | 2.07 | 1.08 |
| 68 | Cymoxanil | 95.71 | 2.62 | 0.94 | 101.68 | 3.00 | 0.89 | 100.82 | 2.28 | 0.87 |
| 69 | Cyproconazole | 92.19 | 2.27 | 0.99 | 93.67 | 0.36 | 1.01 | 96.88 | 0.71 | 0.93 |
| 70 | Deltamethrin | 94.04 | 2.08 | 1.03 | 88.25 | 1.73 | 1.17 | 88.96 | 1.48 | 1.15 |
| 71 | Demeton | 94.83 | 4.48 | 0.96 | 95.13 | 3.33 | 0.98 | 94.24 | 1.62 | 0.89 |
| 72 | Demeton-S-methyl | 98.07 | 6.69 | 1.04 | 92.74 | 1.96 | 1.12 | 93.51 | 2.13 | 1.14 |
| 73 | Demeton-S-methyl sulfone | 94.44 | 2.82 | 0.99 | 101.09 | 4.83 | 0.91 | 99.09 | 2.95 | 0.93 |
| 74 | Demeton-S-sulfone | 91.12 | 2.86 | 1.23 | 90.25 | 5.40 | 1.62 | 96.97 | 3.81 | 1.74 |
| 75 | Demeton-S-sulfoxide | 91.89 | 2.91 | 0.81 | 97.17 | 4.94 | 0.94 | 99.45 | 1.82 | 0.91 |
| 76 | Diazinon | 87.91 | 1.53 | 0.97 | 86.20 | 2.96 | 1.13 | 87.15 | 1.14 | 1.12 |
| 77 | Dichlorvos | 94.28 | 3.41 | 1.10 | 89.76 | 6.09 | 1.34 | 93.12 | 2.64 | 1.42 |
| 78 | Diclobutrazol | 84.00 | 1.76 | 1.11 | 99.12 | 0.52 | 1.03 | 91.77 | 1.39 | 1.08 |
| 79 | Diclofop methyl | 92.74 | 2.25 | 1.19 | 91.02 | 8.15 | 1.48 | 94.99 | 1.63 | 1.60 |
| 80 | Dicrotophos | 96.46 | 3.27 | 0.89 | 99.23 | 1.78 | 0.81 | 97.16 | 1.25 | 0.79 |
| 81 | Diethofencarb | 98.60 | 3.93 | 0.92 | 100.91 | 0.92 | 0.80 | 101.20 | 3.81 | 0.73 |
| 82 | Diethyl aminoethyl hexanoate | 91.38 | 1.36 | 1.05 | 87.35 | 3.51 | 1.16 | 91.37 | 1.11 | 1.19 |
| 83 | Difenoconazole | 73.55 | 1.74 | 1.13 | 90.42 | 2.06 | 1.16 | 90.27 | 0.71 | 1.18 |
| 84 | Diflubenzuron | 95.09 | 2.43 | 1.02 | 94.21 | 2.12 | 1.06 | 97.15 | 1.38 | 1.04 |
| 85 | Diflufenican | 93.03 | 8.38 | 0.76 | 88.95 | 5.81 | 1.27 | 95.96 | 1.76 | 1.27 |
| 86 | Dimepiperate | 90.40 | 4.59 | 1.08 | 92.92 | 9.40 | 1.00 | 97.54 | 7.78 | 0.89 |
| 87 | Dimethenamid | 93.82 | 1.57 | 1.03 | 93.16 | 2.35 | 1.09 | 94.00 | 2.49 | 1.09 |
| 88 | Dimethoate | 96.77 | 2.21 | 0.96 | 98.05 | 2.77 | 0.97 | 98.84 | 3.09 | 0.96 |
| 89 | Dimethomorph | 92.37 | 1.32 | 1.09 | 91.81 | 1.83 | 1.20 | 93.44 | 0.94 | 1.16 |
| 90 | Dimoxystrobin | 93.95 | 1.12 | 1.03 | 95.53 | 1.96 | 1.05 | 95.97 | 0.84 | 1.04 |
| 91 | Diniconazole | 62.51 | 1.44 | 1.08 | 89.03 | 4.22 | 1.10 | 91.54 | 4.92 | 1.04 |
| 92 | Dinocap | 80.22 | 9.35 | 1.15 | 85.43 | 11.12 | 1.34 | 88.06 | 5.06 | 1.26 |
| 93 | Dinotefuran | 96.97 | 2.50 | 0.85 | 98.11 | 1.63 | 0.64 | 102.36 | 2.33 | 0.63 |
| 94 | Disulfoton | 77.74 | 6.82 | 1.28 | 101.58 | 12.85 | 1.00 | 102.32 | 16.01 | 1.08 |
| 95 | Disulfoton sulfone | 91.73 | 0.22 | 1.10 | 92.98 | 3.33 | 1.19 | 95.23 | 1.90 | 1.21 |
| 96 | Disulfoton sulfoxide | 94.04 | 1.90 | 0.94 | 98.73 | 1.71 | 0.87 | 98.29 | 3.02 | 0.82 |
| 97 | Diuron | 92.19 | 1.28 | 1.10 | 89.96 | 4.00 | 1.29 | 95.66 | 1.01 | 1.25 |
| 98 | EPN | 87.96 | 1.82 | 1.22 | 90.59 | 4.67 | 1.46 | 93.59 | 2.19 | 1.48 |
| 99 | Edifenphos | 93.74 | 2.67 | 1.01 | 93.53 | 2.39 | 1.01 | 94.72 | 1.17 | 1.00 |
| 100 | Emamectin B1a | 90.50 | 2.44 | 1.47 | 81.06 | 2.88 | 1.78 | 85.23 | 1.50 | 1.79 |
| 101 | Enestroburin | 93.60 | 0.54 | 1.04 | 93.75 | 3.50 | 1.08 | 94.96 | 1.58 | 1.07 |
| 102 | Epoxiconazole | 85.97 | 1.06 | 1.10 | 91.44 | 4.05 | 1.16 | 92.35 | 2.11 | 1.16 |
| 103 | Ethion | 94.79 | 3.25 | 1.01 | 93.99 | 2.82 | 0.99 | 90.99 | 1.08 | 0.96 |
| 104 | Ethiprole | 115.97 | 12.41 | 0.94 | 97.29 | 3.18 | 0.95 | 106.16 | 7.06 | 0.93 |
| 105 | Ethofumesate | 72.20 | 0.85 | 1.12 | 89.06 | 4.69 | 1.26 | 93.85 | 0.91 | 1.28 |
| 106 | Ethoprophos | 91.47 | 2.75 | 1.18 | 88.63 | 3.64 | 1.40 | 92.53 | 1.18 | 1.44 |
| 107 | Ethoxysulfuron | 90.71 | 4.13 | 0.74 | 101.20 | 1.96 | 0.68 | 99.79 | 1.95 | 0.67 |
| 108 | Etofenprox | 90.92 | 0.88 | 1.02 | 89.73 | 2.50 | 1.02 | 85.61 | 2.69 | 0.99 |
| 109 | Etoxazole | 114.46 | 6.52 | 0.78 | 75.74 | 2.74 | 1.22 | 82.88 | 1.68 | 1.20 |
| 110 | Etrimfos | 87.71 | 1.95 | 1.01 | 90.73 | 4.53 | 1.22 | 92.14 | 1.85 | 1.25 |
| 111 | Famoxadone | 100.73 | 1.29 | 0.93 | 95.68 | 1.09 | 0.96 | 95.87 | 0.97 | 0.93 |
| 112 | Fenamidone | 89.51 | 1.73 | 0.82 | 94.76 | 1.47 | 0.99 | 96.68 | 2.04 | 0.98 |
| 113 | Fenaminstrobin | 90.56 | 2.25 | 1.03 | 90.37 | 1.74 | 1.13 | 95.74 | 1.27 | 1.07 |
| 114 | Fenamiphos | 90.05 | 1.82 | 1.09 | 91.93 | 4.08 | 1.21 | 97.76 | 1.48 | 1.19 |
| 115 | Fenamiphos sulphone | 87.38 | 3.30 | 1.24 | 90.88 | 2.98 | 1.44 | 96.37 | 2.12 | 1.50 |
| 116 | Fenamiphos sulphoxide | 75.04 | 1.58 | 1.31 | 87.11 | 4.05 | 1.56 | 92.07 | 0.77 | 1.71 |
| 117 | Fenarimol | 89.82 | 3.73 | 1.08 | 94.13 | 3.35 | 1.10 | 92.66 | 1.66 | 1.13 |
| 118 | Fenazaquin | 59.43 | 12.31 | 0.30 | 80.70 | 0.97 | 1.06 | 75.50 | 1.64 | 1.07 |
| 119 | Fenbuconazole | 87.57 | 3.62 | 1.05 | 95.11 | 2.50 | 1.02 | 97.32 | 0.86 | 0.96 |
| 120 | Fenhexamid | 86.54 | 3.74 | 1.27 | 91.00 | 5.71 | 1.48 | 93.80 | 3.19 | 1.57 |
| 121 | Fenobucarb | 94.21 | 3.21 | 1.02 | 95.59 | 2.66 | 1.02 | 98.37 | 1.10 | 0.97 |
| 122 | Fenothiocarb | 94.35 | 3.26 | 0.97 | 95.65 | 2.37 | 0.95 | 94.35 | 1.57 | 0.92 |
| 123 | Fenoxanil | 91.23 | 1.64 | 1.12 | 92.24 | 4.21 | 1.23 | 96.07 | 1.26 | 1.26 |
| 124 | Fenoxaprop-ethyl | 60.04 | 4.10 | 0.59 | 87.00 | 5.76 | 1.65 | 94.27 | 2.52 | 1.69 |
| 125 | Fenoxycarb | 91.76 | 1.23 | 1.06 | 95.87 | 2.66 | 1.09 | 97.47 | 1.36 | 1.11 |
| 126 | Fenpropathrin | 98.27 | 3.22 | 0.93 | 89.97 | 2.69 | 1.03 | 87.66 | 1.50 | 1.02 |
| 127 | Fenpyrazamine | 94.49 | 0.53 | 1.09 | 92.27 | 3.95 | 1.19 | 91.98 | 1.24 | 1.22 |
| 128 | Fenpyroximate | 89.12 | 0.63 | 0.98 | 85.95 | 1.71 | 1.01 | 84.10 | 2.14 | 1.00 |
| 129 | Fensulfothion | 75.98 | 0.92 | 1.15 | 91.07 | 4.70 | 1.27 | 95.29 | 1.14 | 1.36 |
| 130 | Fensulfothion oxon | 91.58 | 1.43 | 1.09 | 90.64 | 6.85 | 1.15 | 94.92 | 0.97 | 1.23 |
| 131 | Fensulfothion oxon sulfone | 89.84 | 1.09 | 1.23 | 90.65 | 2.63 | 1.47 | 94.29 | 2.11 | 1.54 |
| 132 | Fenthion | 88.15 | 2.61 | 1.24 | 88.46 | 7.00 | 1.51 | 92.55 | 2.09 | 1.61 |
| 133 | Fenthion sulphone | 91.95 | 5.18 | 1.07 | 91.30 | 2.31 | 1.17 | 93.50 | 0.86 | 1.23 |
| 134 | Fenthion sulfoxide | 72.72 | 0.54 | 1.18 | 83.94 | 1.67 | 1.38 | 95.72 | 2.91 | 1.35 |
| 135 | Fenvalerate | 95.44 | 2.62 | 1.09 | 78.35 | 2.58 | 1.18 | 86.13 | 3.59 | 1.12 |
| 136 | Fipronil | 89.46 | 4.59 | 1.05 | 91.29 | 1.15 | 1.10 | 98.52 | 5.93 | 1.07 |
| 137 | Fipronil desulfinyl | 98.06 | 1.97 | 0.94 | 98.29 | 2.62 | 0.90 | 96.46 | 3.20 | 0.86 |
| 138 | Fipronil sulphide | 97.98 | 1.44 | 0.94 | 95.06 | 3.10 | 0.92 | 95.68 | 2.36 | 0.87 |
| 139 | Fipronil sulphone | 91.48 | 1.88 | 1.23 | 86.66 | 5.59 | 1.63 | 91.24 | 0.85 | 1.72 |
| 140 | Flonicamid | 89.77 | 3.84 | 1.04 | 81.83 | 7.98 | 1.71 | 85.61 | 3.73 | 1.75 |
| 141 | Florasulam | 98.44 | 2.49 | 1.06 | 96.76 | 8.95 | 0.98 | 99.46 | 1.76 | 1.24 |
| 142 | Fluazifop-butyl | 95.41 | 1.00 | 1.04 | 93.28 | 3.70 | 1.10 | 94.51 | 4.52 | 1.11 |
| 143 | Fluazinam | 92.81 | 4.92 | 1.02 | 94.60 | 2.89 | 1.21 | 93.63 | 6.79 | 1.22 |
| 144 | Flubendiamide | 99.03 | 3.05 | 0.99 | 89.14 | 0.98 | 1.21 | 93.63 | 2.11 | 1.24 |
| 145 | Flucetosulfuron | 95.56 | 0.31 | 0.81 | 92.73 | 2.76 | 0.84 | 98.17 | 1.67 | 0.80 |
| 146 | Flucythrinate | 94.93 | 2.44 | 1.09 | 93.51 | 1.35 | 1.09 | 88.92 | 3.02 | 1.11 |
| 147 | Fludioxonil | 95.07 | 3.08 | 1.03 | 93.65 | 3.40 | 1.10 | 97.27 | 2.54 | 1.09 |
| 148 | Flufenacet | 93.47 | 1.23 | 1.09 | 94.78 | 2.48 | 1.16 | 96.57 | 2.43 | 1.15 |
| 149 | Flufenoxuron | 91.69 | 2.63 | 1.02 | 95.43 | 3.90 | 1.01 | 93.58 | 0.46 | 0.96 |
| 150 | Flumetralin | 90.08 | 4.42 | 0.98 | 92.31 | 2.80 | 1.07 | 89.83 | 3.16 | 1.03 |
| 151 | Flumetsulam | 91.66 | 0.50 | 1.29 | 92.41 | 3.79 | 1.12 | 100.49 | 6.31 | 1.30 |
| 152 | Flumorph | 90.31 | 1.89 | 1.15 | 92.36 | 6.28 | 1.32 | 94.94 | 3.26 | 1.24 |
| 153 | Fluopicolide | 93.41 | 0.87 | 1.09 | 93.42 | 2.21 | 1.21 | 97.67 | 2.11 | 1.22 |
| 154 | Fluopyram | 92.53 | 2.79 | 1.04 | 90.54 | 2.75 | 1.18 | 91.17 | 1.00 | 1.20 |
| 155 | Fluoroglycofen-ethyl | 97.96 | 4.51 | 0.89 | 98.11 | 3.07 | 0.85 | 98.16 | 4.20 | 0.81 |
| 156 | Flurtamone | 86.43 | 1.40 | 1.28 | 84.21 | 6.93 | 1.75 | 94.47 | 3.24 | 1.75 |
| 157 | Flusilazole | 66.84 | 2.99 | 1.19 | 90.39 | 5.92 | 1.39 | 96.20 | 2.05 | 1.39 |
| 158 | Fluthiacet-methyl | 85.44 | 1.44 | 1.14 | 96.55 | 3.51 | 1.15 | 98.52 | 3.56 | 1.16 |
| 159 | Flutolanil | 91.80 | 1.50 | 1.15 | 89.11 | 2.80 | 1.36 | 92.58 | 1.72 | 1.38 |
| 160 | Flutriafol | 88.58 | 1.47 | 1.03 | 95.76 | 2.75 | 0.96 | 95.16 | 3.41 | 0.94 |
| 161 | Fluvalinate | 92.46 | 0.99 | 1.12 | 87.74 | 2.60 | 1.15 | 86.92 | 1.51 | 1.14 |
| 162 | Fluxapyroxad | 90.50 | 3.00 | 1.22 | 88.16 | 3.29 | 1.51 | 91.85 | 0.42 | 1.53 |
| 163 | Fonofos | 95.15 | 3.75 | 1.05 | 90.87 | 3.51 | 1.17 | 90.26 | 1.48 | 1.21 |
| 164 | Forchlorfenuron | 86.36 | 1.27 | 1.18 | 93.15 | 3.43 | 1.28 | 93.56 | 2.36 | 1.25 |
| 165 | Formothion | 91.60 | 1.58 | 1.04 | 92.71 | 5.17 | 1.19 | 98.35 | 0.67 | 1.14 |
| 166 | Fosthiazate | 93.00 | 1.22 | 1.07 | 94.36 | 2.87 | 1.12 | 96.74 | 1.35 | 1.10 |
| 167 | Furathiocarb | 93.25 | 2.02 | 0.99 | 95.68 | 1.62 | 0.99 | 94.13 | 1.55 | 0.98 |
| 168 | Heptenophos | 95.18 | 0.93 | 1.02 | 95.41 | 2.42 | 1.05 | 94.64 | 0.75 | 1.06 |
| 169 | Hexaconazole | 86.09 | 4.07 | 0.99 | 93.82 | 0.71 | 0.97 | 90.75 | 0.73 | 0.92 |
| 170 | Hexaflumuron | 99.01 | 0.54 | 1.06 | 89.80 | 4.78 | 1.42 | 91.67 | 4.43 | 1.48 |
| 171 | Hexazinone | 90.88 | 0.90 | 1.05 | 92.70 | 2.50 | 1.18 | 93.37 | 1.89 | 1.21 |
| 172 | Hexythiazox | 92.74 | 3.04 | 1.05 | 93.68 | 2.19 | 1.08 | 88.86 | 1.16 | 1.10 |
| 173 | Imazalil | 87.82 | 1.13 | 1.23 | 87.66 | 4.40 | 1.33 | 95.15 | 0.16 | 1.29 |
| 174 | Imidacloprid | 93.82 | 1.89 | 1.06 | 101.10 | 4.17 | 0.64 | 96.32 | 1.12 | 1.16 |
| 175 | Imidaclothiz | 92.65 | 14.38 | 1.15 | 117.17 | 6.65 | 0.86 | 109.93 | 12.05 | 0.97 |
| 176 | Indoxacarb | 93.40 | 5.58 | 0.91 | 86.89 | 7.23 | 1.34 | 94.10 | 0.62 | 1.36 |
| 177 | Iodosulfuron-methyl | 87.73 | 2.52 | 0.85 | 95.93 | 1.52 | 0.85 | 98.40 | 0.99 | 0.80 |
| 178 | Ipconazole | 80.33 | 4.56 | 0.76 | 91.27 | 6.16 | 0.91 | 100.44 | 1.68 | 0.83 |
| 179 | Iprobenfos | 94.11 | 2.21 | 1.05 | 93.43 | 2.84 | 1.08 | 95.16 | 0.93 | 1.09 |
| 180 | Iprodione | 95.08 | 3.72 | 1.07 | 92.97 | 4.25 | 1.16 | 94.54 | 1.16 | 1.17 |
| 181 | Iprovalicarb | 95.27 | 0.93 | 1.02 | 95.43 | 2.19 | 1.04 | 96.52 | 1.28 | 1.04 |
| 182 | Isazofos | 88.86 | 1.54 | 1.19 | 90.45 | 4.75 | 1.41 | 95.87 | 0.26 | 1.46 |
| 183 | Isocarbofos | 92.18 | 1.42 | 1.13 | 91.08 | 3.41 | 1.28 | 94.25 | 0.93 | 1.33 |
| 184 | Isofenphos-methyl | 101.28 | 12.99 | 0.95 | 100.74 | 7.47 | 0.91 | 90.88 | 2.28 | 0.99 |
| 185 | Isoprocarb | 95.39 | 0.52 | 1.03 | 93.94 | 2.02 | 1.07 | 97.55 | 3.67 | 1.01 |
| 186 | Isoprothiolane | 96.98 | 1.80 | 1.01 | 92.85 | 1.41 | 1.08 | 93.41 | 0.26 | 1.04 |
| 187 | Isoproturon | 96.19 | 2.27 | 1.02 | 94.74 | 1.52 | 1.03 | 94.48 | 1.11 | 0.98 |
| 188 | Isopyrazam | 89.91 | 2.40 | 1.25 | 87.18 | 5.03 | 1.51 | 92.42 | 1.50 | 1.55 |
| 189 | Isoxaflutole | 97.75 | 3.90 | 1.11 | 86.85 | 4.28 | 1.06 | 105.89 | 8.78 | 0.99 |
| 190 | Ivermectin B1a | 79.01 | 4.00 | 1.01 | 56.39 | 14.43 | 1.44 | 78.96 | 12.20 | 1.01 |
| 191 | Kresoxim-methyl | 90.80 | 2.14 | 1.10 | 95.60 | 5.08 | 1.10 | 95.94 | 3.77 | 1.15 |
| 192 | Lactofen | 87.30 | 1.56 | 1.23 | 88.15 | 5.27 | 1.44 | 91.69 | 1.29 | 1.52 |
| 193 | Linuron | 91.72 | 0.92 | 1.06 | 94.01 | 3.46 | 1.11 | 99.40 | 1.10 | 1.09 |
| 194 | Lufenuron | 93.57 | 0.41 | 1.04 | 98.07 | 1.92 | 1.04 | 88.62 | 0.99 | 1.11 |
| 195 | Malaoxon | 95.60 | 1.29 | 1.05 | 94.04 | 3.30 | 1.12 | 97.00 | 2.95 | 1.08 |
| 196 | Malathion | 97.44 | 0.70 | 0.97 | 97.60 | 1.41 | 0.94 | 97.75 | 2.01 | 0.89 |
| 197 | Mandipropamid | 96.06 | 0.98 | 1.05 | 94.91 | 1.50 | 1.11 | 93.45 | 0.84 | 1.11 |
| 198 | Mefenacet | 93.94 | 1.50 | 0.97 | 97.04 | 1.68 | 0.93 | 96.16 | 2.26 | 0.88 |
| 199 | Mepronil | 94.17 | 0.80 | 1.06 | 91.50 | 1.37 | 1.15 | 94.51 | 0.81 | 1.14 |
| 200 | Metaflumizone | 74.06 | 12.39 | 0.32 | 89.25 | 4.52 | 1.24 | 91.48 | 1.94 | 1.26 |
| 201 | Metalaxyl | 93.07 | 0.59 | 1.08 | 95.62 | 2.83 | 1.12 | 98.03 | 0.85 | 1.08 |
| 202 | Metamifop | 63.59 | 9.05 | 0.42 | 99.47 | 1.61 | 0.87 | 95.47 | 1.58 | 0.85 |
| 203 | Metamitron | 82.44 | 2.80 | 1.16 | 89.12 | 5.38 | 1.38 | 93.47 | 2.77 | 1.46 |
| 204 | Metazachlor | 92.49 | 0.85 | 1.10 | 93.05 | 3.00 | 1.22 | 94.41 | 0.63 | 1.17 |
| 205 | Metconazole | 79.24 | 3.45 | 0.96 | 94.22 | 2.75 | 0.97 | 90.39 | 1.73 | 0.94 |
| 206 | Methacrifos | 91.96 | 1.14 | 1.10 | 93.88 | 4.76 | 1.15 | 96.89 | 1.58 | 1.13 |
| 207 | Methamidophos | 84.17 | 13.99 | 0.77 | 122.50 | 9.96 | 0.56 | 109.95 | 8.14 | 0.49 |
| 208 | Methidathion | 90.72 | 1.50 | 1.01 | 96.61 | 1.86 | 0.99 | 93.51 | 2.21 | 0.90 |
| 209 | Methiocarb | 93.88 | 2.24 | 1.02 | 96.42 | 3.36 | 1.01 | 96.64 | 0.30 | 1.00 |
| 210 | Methiocarb sulfone | 95.47 | 2.24 | 0.94 | 93.00 | 5.77 | 0.98 | 96.68 | 3.62 | 0.94 |
| 211 | Methiocarb sulfoxide | 80.64 | 1.46 | 1.17 | 89.24 | 3.77 | 1.19 | 96.61 | 3.51 | 1.35 |
| 212 | Methomyl | 87.00 | 2.33 | 0.41 | 96.44 | 3.50 | 0.80 | 94.16 | 2.30 | 0.80 |
| 213 | Methoxyfenozide | 92.65 | 1.60 | 0.98 | 96.32 | 4.30 | 1.00 | 96.59 | 0.70 | 0.65 |
| 214 | Metolachlor | 91.80 | 0.90 | 1.00 | 94.48 | 3.00 | 1.02 | 94.60 | 2.12 | 1.00 |
| 215 | Metolcarb | 95.59 | 1.54 | 0.94 | 97.90 | 4.95 | 0.94 | 97.43 | 1.82 | 0.94 |
| 216 | Metrafenone | 72.47 | 0.60 | 1.01 | 91.21 | 2.78 | 1.05 | 93.33 | 1.37 | 1.03 |
| 217 | Metribuzin | 85.74 | 4.07 | 1.36 | 83.30 | 4.25 | 1.87 | 91.84 | 1.34 | 2.02 |
| 218 | Metsulfuron-methyl | 91.76 | 1.99 | 0.90 | 99.05 | 5.68 | 0.97 | 101.66 | 1.59 | 0.94 |
| 219 | Mevinphos | 95.02 | 0.89 | 1.04 | 92.68 | 3.64 | 1.10 | 95.44 | 0.82 | 1.12 |
| 220 | Molinate | 97.66 | 0.39 | 1.04 | 87.19 | 1.84 | 1.24 | 88.37 | 3.51 | 1.19 |
| 221 | Monocrotophos | 94.18 | 0.99 | 0.98 | 96.73 | 2.41 | 1.01 | 96.09 | 1.42 | 1.02 |
| 222 | Myclobutanil | 90.83 | 1.21 | 1.06 | 90.94 | 1.34 | 1.12 | 91.08 | 1.00 | 1.12 |
| 223 | Napropamide | 93.22 | 1.22 | 1.02 | 96.03 | 1.73 | 1.01 | 98.25 | 3.16 | 0.95 |
| 224 | Nitenpyram | 88.82 | 1.77 | 1.15 | 91.09 | 3.90 | 1.25 | 95.97 | 2.92 | 1.32 |
| 225 | Novaluron | 91.96 | 0.45 | 1.05 | 91.78 | 2.61 | 1.07 | 95.77 | 3.32 | 1.02 |
| 226 | Omethoate | 92.50 | 0.41 | 1.12 | 92.90 | 4.38 | 1.16 | 98.40 | 1.53 | 1.20 |
| 227 | Orthosulfamuron | 86.57 | 2.34 | 0.83 | 92.98 | 3.19 | 0.88 | 94.57 | 0.54 | 0.91 |
| 228 | Oxadiargyl | 91.78 | 1.29 | 1.07 | 89.47 | 2.29 | 1.15 | 89.89 | 2.57 | 1.18 |
| 229 | Oxadiazon | 87.84 | 1.74 | 1.36 | 84.17 | 7.47 | 1.99 | 87.57 | 3.70 | 2.29 |
| 230 | Oxadixyl | 91.95 | 0.39 | 1.15 | 93.20 | 3.55 | 1.27 | 92.86 | 1.52 | 1.28 |
| 231 | Oxamyl | 100.34 | 4.40 | 0.95 | 91.92 | 2.14 | 0.91 | 95.58 | 2.91 | 0.93 |
| 232 | Oxamyl-oxime | 94.47 | 1.40 | 0.92 | 89.35 | 1.98 | 0.81 | 95.74 | 1.62 | 0.82 |
| 233 | Oxaziclomefone | 92.19 | 0.92 | 1.10 | 92.74 | 2.48 | 1.17 | 94.50 | 0.71 | 1.18 |
| 234 | Oxydemeton-methyl | 92.37 | 1.84 | 1.09 | 94.13 | 3.73 | 1.07 | 95.30 | 0.31 | 1.10 |
| 235 | Oxyfluorfen | 94.22 | 3.49 | 1.06 | 81.39 | 15.29 | 1.26 | 91.41 | 1.76 | 1.22 |
| 236 | Paclobutrazol | 85.92 | 0.70 | 1.21 | 88.50 | 2.97 | 1.48 | 89.68 | 3.53 | 1.59 |
| 237 | Parathion | 87.57 | 3.11 | 1.14 | 95.41 | 8.39 | 1.15 | 105.44 | 3.54 | 1.11 |
| 238 | Penconazole | 73.16 | 3.80 | 1.02 | 96.96 | 4.15 | 0.95 | 94.51 | 1.55 | 0.92 |
| 239 | Pencycuron | 89.14 | 0.76 | 1.16 | 90.48 | 3.09 | 1.21 | 92.05 | 2.98 | 1.24 |
| 240 | Pendimethalin | 83.23 | 3.87 | 1.45 | 87.18 | 6.62 | 1.92 | 95.06 | 3.22 | 2.06 |
| 241 | Penflufen | 86.84 | 1.66 | 0.97 | 92.68 | 1.58 | 0.99 | 89.45 | 0.39 | 0.98 |
| 242 | Penoxsulam | 91.35 | 0.09 | 1.16 | 91.81 | 4.56 | 1.34 | 89.42 | 1.76 | 1.43 |
| 243 | Penthiopyrad | 94.25 | 1.05 | 1.08 | 90.78 | 3.46 | 1.18 | 96.37 | 2.23 | 1.16 |
| 244 | Permethrin | 87.80 | 13.41 | 1.06 | 83.97 | 2.62 | 1.15 | 82.25 | 3.74 | 1.16 |
| 245 | Phenamacril | 91.26 | 0.21 | 1.14 | 92.02 | 2.42 | 1.27 | 97.64 | 1.58 | 1.31 |
| 246 | Phenmedipham | 93.45 | 1.01 | 1.11 | 93.64 | 2.80 | 1.22 | 97.66 | 0.87 | 1.22 |
| 247 | Phenthoate | 92.49 | 1.17 | 1.10 | 91.92 | 3.30 | 1.20 | 94.43 | 0.57 | 1.23 |
| 248 | Phorate | 101.18 | 0.98 | 0.89 | 90.95 | 1.33 | 0.97 | 91.38 | 1.75 | 0.92 |
| 249 | Phorate sulfone | 89.19 | 2.75 | 1.30 | 87.22 | 5.49 | 1.62 | 92.58 | 0.67 | 1.79 |
| 250 | Phorate sulfoxide | 90.77 | 0.36 | 1.14 | 90.87 | 3.49 | 1.29 | 94.63 | 1.35 | 1.30 |
| 251 | Phosalone | 92.47 | 1.01 | 1.02 | 91.87 | 3.21 | 1.02 | 91.06 | 3.30 | 1.02 |
| 252 | Phosfolan | 94.26 | 4.45 | 0.99 | 96.07 | 2.14 | 0.99 | 97.17 | 2.97 | 0.87 |
| 253 | Phosfolan methyl | 95.22 | 1.83 | 0.91 | 95.11 | 3.49 | 0.95 | 100.13 | 3.40 | 0.93 |
| 254 | Phosmet | 92.90 | 2.54 | 1.01 | 96.69 | 2.68 | 1.01 | 96.51 | 1.10 | 1.00 |
| 255 | Phosmet-Oxon | 95.18 | 1.73 | 1.00 | 94.95 | 1.67 | 1.05 | 98.50 | 0.80 | 1.01 |
| 256 | Phosphamidon | 95.49 | 0.88 | 1.02 | 97.46 | 1.20 | 1.03 | 95.98 | 0.93 | 1.02 |
| 257 | Phoxim | 96.38 | 1.70 | 0.97 | 93.75 | 2.01 | 0.98 | 93.54 | 3.37 | 0.94 |
| 258 | Picolinafen | 86.87 | 4.94 | 1.08 | 93.30 | 2.26 | 1.10 | 88.43 | 2.52 | 1.20 |
| 259 | Picoxystrobin | 95.03 | 1.92 | 0.98 | 94.52 | 1.73 | 0.96 | 96.33 | 1.91 | 0.93 |
| 260 | Pirimicarb | 93.49 | 1.10 | 0.97 | 91.85 | 1.54 | 1.02 | 92.27 | 1.20 | 1.00 |
| 261 | Piperonyl butoxide | 90.57 | 2.47 | 1.10 | 95.41 | 3.12 | 1.09 | 96.22 | 1.04 | 1.07 |
| 262 | Pirimicarb desmethyl | 94.75 | 0.70 | 1.63 | 92.63 | 4.58 | 1.42 | 95.34 | 1.19 | 1.44 |
| 263 | Pirimiphos-methyl | 87.37 | 2.77 | 1.25 | 89.05 | 4.51 | 1.39 | 90.26 | 2.57 | 1.41 |
| 264 | Pretilachlor | 94.25 | 1.82 | 1.00 | 91.24 | 4.14 | 1.10 | 92.47 | 1.83 | 1.10 |
| 265 | Probenazole | 96.97 | 1.98 | 0.90 | 101.90 | 1.70 | 0.77 | 99.73 | 2.80 | 0.72 |
| 266 | Prochloraz | 60.05 | 1.30 | 1.13 | 89.06 | 2.33 | 1.25 | 89.34 | 0.51 | 1.26 |
| 267 | Procymidone | 84.32 | 3.94 | 1.40 | 79.19 | 6.98 | 1.88 | 90.92 | 2.59 | 1.82 |
| 268 | Profenofos | 91.31 | 3.24 | 1.01 | 92.34 | 4.57 | 1.10 | 91.90 | 2.15 | 1.09 |
| 269 | Promecarb | 96.37 | 2.67 | 0.97 | 96.86 | 2.72 | 0.96 | 97.85 | 0.37 | 0.93 |
| 270 | Prometryn | 90.14 | 0.75 | 1.20 | 90.32 | 3.53 | 1.34 | 91.52 | 0.33 | 1.37 |
| 271 | Propachlor | 94.82 | 1.46 | 1.01 | 96.53 | 3.01 | 1.00 | 94.02 | 2.51 | 0.86 |
| 272 | Propamocarb | 108.78 | 1.03 | 1.52 | 102.83 | 2.60 | 1.56 | 104.83 | 1.56 | 1.65 |
| 273 | Propanil | 88.35 | 0.94 | 1.21 | 88.78 | 5.79 | 1.45 | 98.34 | 1.62 | 1.44 |
| 274 | Propaquizafop | 93.43 | 6.42 | 0.85 | 99.64 | 1.57 | 0.79 | 92.83 | 1.70 | 0.75 |
| 275 | Propargite | 96.14 | 0.58 | 0.94 | 94.75 | 1.32 | 0.91 | 93.08 | 2.09 | 0.84 |
| 276 | Propiconazole | 81.42 | 0.20 | 1.08 | 92.72 | 0.87 | 1.16 | 88.57 | 2.17 | 1.13 |
| 277 | Propoxur | 94.28 | 0.78 | 1.12 | 92.32 | 2.36 | 1.20 | 94.13 | 1.21 | 1.20 |
| 278 | Propyrisulfuron | 85.31 | 0.29 | 1.02 | 97.86 | 2.96 | 1.05 | 96.25 | 2.03 | 1.07 |
| 279 | Propyzamide | 99.15 | 2.90 | 0.95 | 95.12 | 0.49 | 0.95 | 94.84 | 1.51 | 0.91 |
| 280 | Proquinazid | 86.03 | 2.97 | 1.00 | 86.77 | 1.53 | 1.10 | 83.57 | 1.65 | 1.11 |
| 281 | Prosulfocarb | 94.03 | 3.88 | 0.93 | 92.12 | 4.56 | 1.03 | 94.24 | 1.10 | 1.00 |
| 282 | Pyraclostrobin | 97.02 | 2.90 | 0.93 | 97.41 | 2.05 | 0.87 | 94.66 | 2.47 | 0.82 |
| 283 | Pyraflufen-Ethyl | 93.71 | 0.82 | 1.12 | 88.34 | 1.38 | 1.32 | 91.51 | 1.33 | 1.37 |
| 284 | Pyrametostrobin | 90.13 | 0.45 | 1.09 | 92.39 | 3.00 | 1.14 | 93.30 | 0.99 | 1.14 |
| 285 | Pyraoxystrobin | 86.48 | 0.50 | 1.09 | 93.51 | 2.97 | 1.14 | 92.57 | 1.79 | 1.16 |
| 286 | Pyrazosulfuron-ethyl | 86.52 | 2.69 | 0.95 | 95.25 | 4.28 | 0.96 | 97.91 | 0.92 | 1.03 |
| 287 | Pyrethrins I | 99.99 | 0.19 | 0.99 | 91.44 | 1.95 | 1.08 | 91.36 | 2.32 | 1.06 |
| 288 | Pyribenzoxim | 61.12 | 9.08 | 1.11 | 110.67 | 4.80 | 0.66 | 92.12 | 7.07 | 0.80 |
| 289 | Pyridaben | 89.90 | 1.51 | 1.11 | 86.55 | 1.63 | 1.04 | 84.58 | 0.16 | 1.12 |
| 290 | Pyridalyl | 87.43 | 10.64 | 1.31 | 81.84 | 3.74 | 1.56 | 79.06 | 1.35 | 1.56 |
| 291 | Pyridaphenthion | 82.94 | 1.80 | 1.06 | 94.27 | 4.88 | 1.12 | 91.45 | 0.17 | 1.18 |
| 292 | Pyriftalid | 78.83 | 1.19 | 1.21 | 95.47 | 4.41 | 1.28 | 97.66 | 1.16 | 1.30 |
| 293 | Pyrimethanil | 85.08 | 0.94 | 1.38 | 88.13 | 5.50 | 1.66 | 92.60 | 0.92 | 1.71 |
| 294 | Pyriproxyfen | 92.75 | 2.77 | 0.94 | 91.29 | 1.15 | 1.01 | 91.44 | 0.66 | 0.99 |
| 295 | Pyrisoxazole | 88.22 | 1.53 | 1.48 | 85.49 | 4.40 | 1.85 | 89.00 | 1.17 | 1.88 |
| 296 | Quinalphos | 88.48 | 0.21 | 1.12 | 93.00 | 4.26 | 1.23 | 94.26 | 1.08 | 1.26 |
| 297 | Quizalofop-ethyl | 85.85 | 1.55 | 1.02 | 93.32 | 2.39 | 1.07 | 95.65 | 1.26 | 1.07 |
| 298 | Rotenone | 88.39 | 0.51 | 1.10 | 90.32 | 4.13 | 1.19 | 97.68 | 1.82 | 1.24 |
| 299 | Saflufenacil | 95.42 | 4.84 | 1.04 | 97.92 | 4.31 | 1.03 | 97.75 | 0.60 | 1.05 |
| 300 | Sedaxane | 93.35 | 1.66 | 1.09 | 93.89 | 2.72 | 1.19 | 93.05 | 0.85 | 1.24 |
| 301 | Sethoxydim | 91.70 | 2.51 | 0.89 | 89.14 | 2.57 | 0.92 | 90.00 | 1.83 | 0.86 |
| 302 | Silthiofam | 88.83 | 1.15 | 0.94 | 92.10 | 3.35 | 1.27 | 94.79 | 0.42 | 1.30 |
| 303 | Simazine | 91.52 | 1.02 | 1.17 | 88.64 | 1.93 | 1.39 | 92.03 | 0.87 | 1.45 |
| 304 | Simetryn | 85.53 | 0.57 | 1.36 | 88.76 | 4.91 | 1.58 | 91.40 | 0.80 | 1.59 |
| 305 | Spinetoram (J) | 91.72 | 1.37 | 1.12 | 75.57 | 2.91 | 1.28 | 78.97 | 0.31 | 1.28 |
| 306 | Spinetoram (L) | 92.43 | 0.50 | 1.16 | 74.40 | 3.04 | 1.29 | 76.59 | 1.58 | 1.27 |
| 307 | Spinosad A | 91.51 | 0.86 | 1.19 | 79.40 | 5.24 | 1.31 | 83.70 | 0.58 | 1.29 |
| 308 | Spinosad D | 94.69 | 3.58 | 1.10 | 79.33 | 0.49 | 1.16 | 78.05 | 1.91 | 1.18 |
| 309 | Spirodiclofen | 97.15 | 1.99 | 0.75 | 94.67 | 1.41 | 0.73 | 90.18 | 4.01 | 0.70 |
| 310 | Spirotetramat | 94.54 | 0.19 | 1.07 | 93.54 | 2.32 | 1.15 | 96.02 | 2.43 | 1.18 |
| 311 | Spirotetramat enol | 88.69 | 11.72 | 1.40 | 93.88 | 5.95 | 1.73 | 111.23 | 7.07 | 1.59 |
| 312 | Spirotetramat ketohydroxy | 89.84 | 2.30 | 1.26 | 89.17 | 5.88 | 1.60 | 94.70 | 0.24 | 1.71 |
| 313 | Spirotetramat monohydroxy | 87.41 | 2.47 | 1.43 | 88.48 | 5.89 | 1.83 | 89.23 | 2.30 | 2.14 |
| 314 | Spirotetramat-enol-glucoside | 92.41 | 2.29 | 0.94 | 95.26 | 4.29 | 0.94 | 94.68 | 0.32 | 0.96 |
| 315 | Sulfentrazone | 93.55 | 1.12 | 1.07 | 92.63 | 2.48 | 1.13 | 98.18 | 1.66 | 1.19 |
| 316 | Sulfotep | 97.41 | 2.77 | 0.94 | 95.60 | 1.62 | 0.93 | 93.69 | 2.68 | 0.89 |
| 317 | Sulfoxaflor | 95.39 | 7.41 | 0.81 | 98.74 | 3.45 | 0.94 | 98.55 | 1.66 | 0.90 |
| 318 | Tebuconazole | 78.74 | 5.32 | 0.98 | 94.01 | 2.89 | 1.00 | 91.74 | 0.91 | 0.98 |
| 319 | Tebufenozide | 99.88 | 5.88 | 0.86 | 107.84 | 2.33 | 0.71 | 102.15 | 4.04 | 0.67 |
| 320 | Tebuthiuron | 91.16 | 1.13 | 1.11 | 91.52 | 2.38 | 1.24 | 92.53 | 1.48 | 1.25 |
| 321 | Teflubenzuron | 94.94 | 1.83 | 1.02 | 96.31 | 6.97 | 1.12 | 92.69 | 2.20 | 1.11 |
| 322 | Terbufos sulfone | 97.55 | 2.49 | 1.00 | 94.48 | 2.60 | 1.04 | 95.68 | 1.08 | 1.04 |
| 323 | Terbufos sulfoxide | 97.25 | 1.78 | 0.97 | 97.10 | 2.63 | 0.95 | 95.72 | 1.36 | 0.92 |
| 324 | Terbuthylazine | 94.13 | 0.95 | 1.06 | 95.53 | 1.78 | 1.09 | 95.75 | 0.96 | 1.10 |
| 325 | Tetraconazole | 82.34 | 2.22 | 1.19 | 92.59 | 4.96 | 1.29 | 90.80 | 1.03 | 1.34 |
| 326 | Thiabendazole | 80.37 | 1.09 | 2.61 | 87.21 | 6.11 | 3.16 | 89.35 | 2.49 | 3.48 |
| 327 | Thiacloprid | 91.59 | 1.00 | 1.10 | 95.13 | 5.85 | 1.21 | 95.32 | 1.68 | 1.26 |
| 328 | Thiamethoxam | 98.93 | 2.54 | 0.72 | 99.01 | 3.51 | 0.84 | 100.05 | 4.19 | 0.77 |
| 329 | Thidiazuron | 72.18 | 1.50 | 1.17 | 93.78 | 5.24 | 1.25 | 94.39 | 0.50 | 1.26 |
| 330 | Thifensulfuron methyl | 88.67 | 3.74 | 0.91 | 93.95 | 2.73 | 1.01 | 97.36 | 0.30 | 0.96 |
| 331 | Thifluzamide | 91.41 | 1.43 | 1.18 | 88.62 | 5.17 | 1.38 | 96.09 | 1.14 | 1.33 |
| 332 | Thiophanate-methyl | 87.89 | 1.20 | 1.01 | 90.44 | 3.42 | 1.44 | 92.21 | 2.08 | 1.55 |
| 333 | Tolclofos-methyl | 77.35 | 2.79 | 1.17 | 86.53 | 0.82 | 1.46 | 89.68 | 1.45 | 1.50 |
| 334 | Tolfenpyrad | 94.38 | 0.98 | 1.08 | 89.04 | 5.81 | 1.29 | 93.60 | 2.15 | 1.31 |
| 335 | Tralkoxydim | 88.96 | 0.71 | 1.03 | 84.99 | 2.88 | 1.26 | 87.32 | 0.53 | 1.27 |
| 336 | Triadimefon | 89.05 | 1.15 | 1.17 | 90.06 | 3.55 | 1.35 | 92.17 | 2.22 | 1.40 |
| 337 | Triadimenol | 94.70 | 2.01 | 0.95 | 97.51 | 3.06 | 0.93 | 92.57 | 1.63 | 0.69 |
| 338 | Triallate | 89.98 | 3.07 | 1.18 | 88.33 | 1.44 | 1.41 | 86.13 | 0.57 | 1.44 |
| 339 | Triasulfuron | 91.03 | 4.25 | 1.01 | 95.28 | 4.12 | 1.11 | 96.92 | 3.01 | 1.08 |
| 340 | Triazophos | 92.65 | 1.47 | 1.06 | 93.22 | 0.79 | 1.08 | 95.50 | 1.39 | 1.06 |
| 341 | Trichlorfon | 87.31 | 3.92 | 1.13 | 90.04 | 4.91 | 1.36 | 96.45 | 0.68 | 1.35 |
| 342 | Trifloxystrobin | 94.83 | 3.53 | 1.01 | 92.66 | 3.78 | 1.16 | 93.39 | 0.60 | 1.17 |
| 343 | Triflumizole | 86.72 | 2.15 | 0.91 | 91.24 | 3.25 | 1.13 | 89.70 | 1.20 | 1.13 |
| 344 | Triflumuron | 91.30 | 1.01 | 1.07 | 89.26 | 3.44 | 1.17 | 93.08 | 2.05 | 1.13 |
| 345 | Triflusulfuron-methyl | 91.78 | 1.44 | 0.83 | 94.11 | 4.14 | 0.83 | 97.64 | 2.14 | 0.81 |
| 346 | Triticonazole | 86.60 | 0.91 | 1.03 | 96.98 | 3.07 | 1.01 | 95.10 | 1.75 | 0.99 |
| 347 | Tritosulfuron | 93.79 | 1.86 | 1.01 | 93.23 | 2.29 | 1.16 | 97.10 | 2.22 | 1.18 |
| 348 | Vamidothion | 93.95 | 0.28 | 0.95 | 99.80 | 2.22 | 0.89 | 98.80 | 2.18 | 0.89 |
| 349 | Zoxamide | 93.79 | 2.98 | 1.03 | 92.03 | 2.49 | 1.07 | 91.56 | 1.23 | 1.08 |
| 350 | Benazolin-ethyl | 88.58 | 1.66 | 1.20 | 89.72 | 4.86 | 1.43 | 96.16 | 0.60 | 1.45 |
| 351 | Cyazofamid metabolite CCIM | 85.36 | 1.61 | 1.03 | 87.17 | 7.75 | 1.11 | 98.70 | 4.51 | 0.95 |
| 352 | Fensulfothion sulfone | 90.54 | 1.48 | 1.17 | 91.49 | 4.40 | 1.32 | 92.91 | 1.71 | 1.32 |
| 353 | Mesosulfuron methyl | 90.69 | 1.18 | 0.93 | 90.70 | 6.50 | 0.95 | 100.90 | 2.14 | 0.96 |
| 354 | Pirimicarb-desmethyl-formamido | 73.36 | 2.97 | 0.70 | 95.76 | 2.16 | 0.99 | 98.72 | 1.05 | 0.94 |
| 355 | Prochloraz metabolite BTS44596 | 81.90 | 1.02 | 1.06 | 87.73 | 3.14 | 1.41 | 92.43 | 1.28 | 1.47 |
| 356 | Propisochlor | 95.17 | 2.74 | 0.96 | 100.32 | 2.11 | 0.87 | 95.09 | 1.79 | 0.83 |
| 357 | Pyrethrins II | 91.14 | 6.84 | 0.97 | 96.81 | 3.93 | 1.01 | 96.57 | 2.77 | 0.99 |
| 358 | Pyrimorph | 92.53 | 2.44 | 1.05 | 98.88 | 1.97 | 0.94 | 94.88 | 1.79 | 0.87 |
| 359 | Spiromesifen | 87.05 | 12.58 | 1.26 | 98.02 | 21.77 | 1.14 | 129.32 | 26.03 | 0.82 |
| 360 | Terbufos | 83.64 | 4.71 | 1.27 | 97.62 | 6.99 | 1.32 | 109.54 | 10.12 | 1.11 |
| 361 | triflumizole Metabolite FM-6-1 | 96.11 | 4.70 | 0.95 | 101.31 | 1.58 | 0.85 | 94.26 | 2.66 | 0.85 |
| 362 | Uniconazole | 74.00 | 2.40 | 1.06 | 97.14 | 2.87 | 1.03 | 94.89 | 4.01 | 1.01 |
| 363 | Xylazine | 107.51 | 6.14 | 1.06 | 100.59 | 10.67 | 0.83 | 82.78 | 3.04 | 0.97 |
| 364 | Pindolol | 106.06 | 0.63 | 1.21 | 109.89 | 8.25 | 0.98 | 75.13 | 3.10 | 1.33 |
| 365 | Alprenolol | 99.31 | 2.69 | 1.16 | 95.13 | 4.45 | 1.04 | 82.31 | 3.64 | 1.19 |
| 366 | Propranolol | 95.94 | 2.97 | 1.14 | 96.42 | 2.20 | 1.04 | 84.77 | 7.33 | 1.16 |
| 367 | Oxprenolol | 101.26 | 0.75 | 1.02 | 99.59 | 2.61 | 0.94 | 86.56 | 7.14 | 1.05 |
| 368 | Acebutolol | 102.08 | 2.93 | 1.22 | 99.95 | 7.02 | 1.07 | 75.59 | 4.71 | 1.37 |
| 369 | Metoprolol | 111.09 | 6.52 | 1.25 | 106.12 | 8.05 | 1.00 | 77.81 | 10.00 | 1.37 |
| 370 | Levobunolol | 91.00 | 1.70 | 1.05 | 90.82 | 3.56 | 1.10 | 112.80 | 3.72 | 0.95 |
| 371 | Carazolol | 100.82 | 3.14 | 1.06 | 97.20 | 1.71 | 0.92 | 77.11 | 6.86 | 1.16 |
| 372 | Timolol | 106.68 | 4.01 | 1.18 | 99.17 | 3.40 | 1.05 | 74.92 | 3.92 | 1.30 |
| 373 | Betaxolol | 92.77 | 2.25 | 1.11 | 99.10 | 3.37 | 0.99 | 98.47 | 5.36 | 1.01 |
| 374 | Droperidol | 91.29 | 3.93 | 1.28 | 90.12 | 3.55 | 1.24 | 83.00 | 4.08 | 1.36 |
| 375 | Carvedilol | 82.09 | 0.69 | 1.42 | 93.26 | 6.46 | 1.06 | 89.45 | 9.13 | 1.10 |
| 376 | Nebivolol | 85.73 | 3.98 | 1.22 | 96.86 | 1.38 | 1.02 | 87.34 | 0.61 | 1.12 |
| 377 | Acepromazine | 96.22 | 1.06 | 1.09 | 82.66 | 2.52 | 0.94 | 75.35 | 3.86 | 1.09 |
| 378 | Chlorpromazine | 94.10 | 1.71 | 1.10 | 83.70 | 4.01 | 0.81 | 69.08 | 3.89 | 0.99 |
| 379 | Nadolol | 97.79 | 1.79 | 1.49 | 99.92 | 3.32 | 1.53 | 119.75 | 5.73 | 1.37 |
| 380 | Estazolam | 54.96 | 2.67 | 0.99 | 90.30 | 3.66 | 0.99 | 81.71 | 4.37 | 1.03 |
| 381 | Penbutolol | 98.35 | 2.05 | 1.21 | 94.73 | 2.81 | 0.98 | 78.02 | 6.90 | 1.26 |
| 382 | Demoxepam | 73.83 | 5.63 | 0.83 | 98.90 | 6.46 | 0.96 | 81.51 | 6.96 | 1.03 |
| 383 | Diazapam | 92.15 | 4.19 | 0.94 | 87.21 | 2.75 | 0.97 | 75.84 | 15.55 | 1.15 |
| 384 | Nitrazepam | 101.21 | 0.92 | 0.83 | 105.84 | 11.98 | 0.71 | 59.24 | 16.30 | 1.27 |
| 385 | Acetylkitasamycin | 94.01 | 2.52 | 0.96 | 89.15 | 6.43 | 1.03 | 78.49 | 7.00 | 1.20 |
| 386 | Narasin | 100.03 | 6.36 | 1.00 | 96.46 | 14.73 | 0.69 | 70.46 | 8.12 | 1.13 |
| 387 | Salinomycin | 96.83 | 8.96 | 0.71 | 98.84 | 7.35 | 0.77 | 52.06 | 19.63 | 1.34 |
| 388 | Monensin | 76.22 | 9.25 | 0.91 | 106.55 | 1.37 | 0.71 | 67.99 | 4.58 | 1.12 |
| 389 | luftong | 87.31 | 2.45 | 1.62 | 92.28 | 3.33 | 1.72 | 112.36 | 5.51 | 1.46 |
| 390 | Sulfanitran | 112.34 | 4.55 | 0.89 | 93.09 | 3.46 | 1.00 | 93.96 | 7.53 | 1.10 |
| 391 | Guanabenz | 66.39 | 6.51 | 1.26 | 86.65 | 4.53 | 1.08 | 74.62 | 1.96 | 1.33 |
| 392 | Trimethoprim | 88.63 | 3.84 | 1.38 | 106.71 | 3.15 | 1.12 | 85.43 | 2.04 | 1.40 |
| 393 | Diaveridine | 104.69 | 6.12 | 1.28 | 100.19 | 5.46 | 1.12 | 83.97 | 10.29 | 1.32 |
| 394 | Ethopabate | 93.23 | 1.13 | 0.97 | 95.40 | 3.67 | 0.97 | 92.20 | 1.77 | 1.01 |
| 395 | Sulfachlorpyridazine | 104.25 | 1.07 | 1.00 | 90.45 | 4.00 | 1.10 | 85.75 | 7.00 | 1.16 |
| 396 | Sulfamonomethoxine | 98.84 | 1.81 | 1.01 | 94.74 | 5.98 | 1.02 | 80.82 | 6.02 | 1.16 |
| 397 | Sulfisomidine | 89.88 | 0.60 | 1.08 | 92.02 | 8.90 | 1.10 | 88.70 | 3.51 | 1.14 |
| 398 | Sulfabenzamine | 95.09 | 1.45 | 0.92 | 92.47 | 1.27 | 1.00 | 101.74 | 1.94 | 0.90 |
| 399 | Sulfamethizole | 91.45 | 4.23 | 1.02 | 95.25 | 4.97 | 1.13 | 96.30 | 1.72 | 1.11 |
| 400 | Sulfamoxole | 93.72 | 3.02 | 0.92 | 91.08 | 2.48 | 1.12 | 105.34 | 2.19 | 0.98 |
| 401 | Sulfisoxazole | 91.41 | 3.11 | 0.98 | 91.16 | 5.74 | 1.07 | 92.53 | 4.05 | 1.06 |
| 402 | Sulfamerazine | 93.37 | 2.05 | 1.00 | 100.50 | 6.50 | 0.94 | 84.47 | 2.49 | 1.08 |
| 403 | Sulfathiazole | 90.90 | 5.14 | 0.86 | 83.45 | 5.13 | 1.25 | 111.17 | 8.15 | 0.98 |
| 404 | Sulfamethoxazole | 97.26 | 4.30 | 0.99 | 100.99 | 0.59 | 0.96 | 83.25 | 2.68 | 1.12 |
| 405 | Sulfapyridine | 99.08 | 2.56 | 0.92 | 101.01 | 4.12 | 0.94 | 90.72 | 2.07 | 0.97 |
| 406 | Sulfaguanidine | 99.28 | 8.65 | 0.94 | 87.79 | 9.85 | 1.05 | 107.53 | 3.43 | 0.89 |
| 407 | Sulfaquinoxaline | 94.70 | 3.32 | 0.97 | 94.73 | 2.13 | 0.98 | 81.01 | 2.61 | 1.12 |
| 408 | Sulfadimethoxine | 99.03 | 2.31 | 1.17 | 101.08 | 2.56 | 1.03 | 72.41 | 7.44 | 1.42 |
| 409 | Sulfadoxine | 100.09 | 2.08 | 1.00 | 105.52 | 2.27 | 0.90 | 76.62 | 6.36 | 1.19 |
| 410 | Sulfadimidine | 86.95 | 3.59 | 1.05 | 93.18 | 6.07 | 0.99 | 84.81 | 3.31 | 1.09 |
| 411 | Ronidazole | 98.94 | 4.30 | 0.90 | 91.32 | 3.23 | 0.87 | 86.12 | 1.54 | 0.98 |
| 412 | Metronidazole-hydroxy | 74.76 | 8.04 | 1.18 | 91.30 | 5.21 | 1.14 | 75.21 | 11.86 | 1.32 |
| 413 | Metronidazole | 97.25 | 1.63 | 0.96 | 92.59 | 1.71 | 0.89 | 85.12 | 2.47 | 0.97 |
| 414 | Ipronidazole | 108.19 | 4.99 | 0.86 | 110.39 | 9.14 | 0.60 | 69.75 | 11.07 | 0.86 |
| 415 | 5-nitrobenzimidazole | 94.34 | 2.45 | 1.33 | 100.78 | 7.00 | 1.10 | 70.31 | 5.50 | 1.39 |
| 416 | 5-chloro-1-methyl-4-nitroimidazole | 110.90 | 1.84 | 0.79 | 90.35 | 5.58 | 0.67 | 79.44 | 4.03 | 0.69 |
| 417 | Dimetridazole-2-hydroxy | 83.69 | 14.13 | 0.91 | 120.30 | 11.93 | 0.58 | 88.16 | 6.34 | 0.72 |
| 418 | Dimetridazole | 105.06 | 2.35 | 0.80 | 106.74 | 7.28 | 0.62 | 93.58 | 3.93 | 0.67 |
| 419 | 4-nitroimidazole | 115.82 | 0.86 | 0.91 | 112.63 | 23.68 | 0.57 | 3267.79 | 4.35 | 0.12 |
| 420 | Enoxacin | 98.59 | 0.54 | 1.15 | 98.37 | 2.29 | 1.20 | 108.79 | 8.63 | 1.14 |
| 421 | Kitasamycin | 114.49 | 12.56 | 0.79 | 101.88 | 2.49 | 0.90 | 80.95 | 17.24 | 1.11 |
| 422 | Flumequine | 101.17 | 4.24 | 0.88 | 95.08 | 6.95 | 1.02 | 88.12 | 4.44 | 1.07 |
| 423 | Roxithromycin | 97.91 | 5.42 | 0.88 | 91.39 | 3.93 | 1.10 | 88.93 | 0.48 | 1.17 |
| 424 | Virginiamycin S1 | 98.96 | 3.32 | 0.93 | 107.53 | 10.19 | 0.70 | 64.25 | 10.18 | 1.14 |
| 425 | Rifampicin | 81.24 | 7.98 | 0.90 | 80.35 | 2.26 | 0.33 | 78.42 | 10.68 | 0.56 |
| 426 | Midecamycin | 93.48 | 2.29 | 0.96 | 94.90 | 0.56 | 0.97 | 98.23 | 3.05 | 0.98 |
| 427 | Desmycosin | 97.89 | 1.02 | 1.52 | 92.77 | 2.59 | 1.19 | 85.78 | 5.11 | 1.30 |
| 428 | Oleandomycin | 97.48 | 3.42 | 1.09 | 88.57 | 2.96 | 1.15 | 95.66 | 4.90 | 1.11 |
| 429 | Virginiamycin M1 | 86.49 | 1.08 | 0.98 | 100.04 | 4.35 | 0.90 | 81.53 | 3.66 | 1.09 |
| 430 | Lincomycin | 103.66 | 2.96 | 2.94 | 100.04 | 1.70 | 3.15 | 100.53 | 2.40 | 3.17 |
| 431 | Tylosin tartrate | 91.40 | 7.63 | 0.73 | 99.26 | 1.80 | 0.91 | 82.53 | 11.14 | 1.17 |
| 432 | Acetanilide | 107.44 | 2.23 | 0.75 | 98.56 | 8.23 | 0.64 | 93.49 | 6.45 | 0.60 |
| 433 | Benzocaine | 103.16 | 1.48 | 0.80 | 91.57 | 4.50 | 0.81 | 93.64 | 3.14 | 0.75 |
| 434 | Detomidine | 102.16 | 2.41 | 1.16 | 90.89 | 2.05 | 1.25 | 98.04 | 0.65 | 1.18 |
| 435 | Levamisole | 98.82 | 6.97 | 1.11 | 99.69 | 3.83 | 0.86 | 92.06 | 0.57 | 0.89 |
| 436 | Clonidine | 101.89 | 4.45 | 1.03 | 86.55 | 1.89 | 1.13 | 95.73 | 2.86 | 1.09 |
| 437 | Lidocaine | 97.82 | 2.91 | 1.05 | 91.27 | 1.20 | 1.02 | 94.34 | 1.45 | 1.00 |
| 438 | flurbiprofen | 96.49 | 6.56 | 1.09 | 98.96 | 2.41 | 1.06 | 99.73 | 15.84 | 1.77 |
| 439 | Fenbufen | 55.20 | 5.76 | 1.13 | 114.34 | 6.29 | 0.89 | 88.44 | 5.19 | 1.41 |
| 440 | Diphenhydramine | 92.25 | 1.57 | 1.09 | 89.80 | 2.57 | 1.05 | 96.32 | 0.57 | 1.02 |
| 441 | Clenproperol | 100.79 | 1.16 | 1.12 | 93.99 | 2.34 | 1.12 | 92.30 | 2.17 | 1.14 |
| 442 | Antazoline | 88.84 | 3.72 | 1.16 | 96.49 | 4.85 | 1.03 | 86.18 | 2.13 | 1.14 |
| 443 | Chlormezanone | 97.99 | 6.96 | 0.91 | 96.81 | 4.08 | 0.87 | 82.39 | 5.08 | 1.01 |
| 444 | Clotrimazole | 97.28 | 6.16 | 1.09 | 112.27 | 3.75 | 0.83 | 75.62 | 12.71 | 1.23 |
| 445 | Doxepin | 92.40 | 4.21 | 1.10 | 87.03 | 1.36 | 1.03 | 88.48 | 9.83 | 1.07 |
| 446 | Cyproheptadine | 97.13 | 4.61 | 1.02 | 85.85 | 4.85 | 1.07 | 81.84 | 1.64 | 1.05 |
| 447 | Clencyclohexerol | 97.55 | 3.72 | 1.03 | 92.44 | 1.23 | 1.00 | 91.37 | 1.30 | 1.03 |
| 448 | Clenpenterol | 97.55 | 3.72 | 1.03 | 92.44 | 1.23 | 1.00 | 91.37 | 1.30 | 1.03 |
| 449 | Anastrozole | 99.11 | 1.52 | 0.89 | 97.47 | 3.33 | 0.93 | 89.53 | 4.01 | 1.02 |
| 450 | Diclofenac | 86.18 | 6.52 | 1.02 | 105.37 | 2.11 | 0.93 | 91.73 | 7.05 | 1.03 |
| 451 | Isoxsuprine | 100.08 | 4.29 | 1.00 | 89.67 | 3.08 | 1.08 | 98.60 | 0.93 | 1.01 |
| 452 | Fluconazole | 77.53 | 6.65 | 1.20 | 97.99 | 5.23 | 1.17 | 82.40 | 3.81 | 1.34 |
| 453 | Ketotifen | 99.72 | 4.13 | 1.19 | 93.25 | 2.14 | 1.04 | 75.23 | 3.05 | 1.30 |
| 454 | Bifonazole | 90.20 | 5.29 | 1.04 | 91.86 | 1.66 | 1.05 | 91.00 | 5.47 | 1.03 |
| 455 | Kresoxim-Methyl | 96.24 | 1.64 | 0.94 | 90.57 | 2.01 | 0.97 | 99.59 | 1.83 | 0.89 |
| 456 | Clomipramine | 100.22 | 2.12 | 1.07 | 94.17 | 2.30 | 0.98 | 80.41 | 6.34 | 1.21 |
| 457 | Chlorprothixene | 99.27 | 7.19 | 1.11 | 92.14 | 6.44 | 0.99 | 86.16 | 5.18 | 1.19 |
| 458 | Gliclazide | 92.97 | 2.72 | 0.76 | 93.04 | 2.20 | 0.95 | 91.16 | 2.08 | 1.00 |
| 459 | Citalopram | 95.73 | 2.63 | 1.04 | 93.42 | 5.55 | 0.99 | 90.25 | 7.80 | 1.05 |
| 460 | Danazol | 84.41 | 5.92 | 0.84 | 89.48 | 6.59 | 0.92 | 82.33 | 1.89 | 0.97 |
| 461 | Griseofulvin | 78.81 | 3.26 | 1.03 | 88.32 | 6.09 | 1.07 | 89.50 | 5.41 | 1.10 |
| 462 | Bisacodyl | 91.57 | 0.60 | 1.06 | 90.22 | 2.62 | 1.08 | 90.87 | 2.45 | 1.12 |
| 463 | Hydrocortisone | 99.14 | 7.68 | 1.18 | 104.13 | 4.73 | 1.13 | 84.83 | 3.36 | 1.38 |
| 464 | Bumetanide | 60.36 | 5.59 | 1.07 | 103.49 | 1.62 | 0.91 | 79.50 | 8.52 | 1.19 |
| 465 | Indapamide | 72.94 | 4.18 | 0.80 | 89.30 | 16.18 | 0.73 | 56.83 | 10.26 | 1.26 |
| 466 | Bromhexine | 101.87 | 1.10 | 1.00 | 87.91 | 5.06 | 1.05 | 76.85 | 2.34 | 1.19 |
| 467 | Doxapram | 98.07 | 4.57 | 1.14 | 100.26 | 2.80 | 0.96 | 75.86 | 5.53 | 1.24 |
| 468 | Econazol | 84.18 | 3.48 | 1.19 | 91.64 | 1.57 | 0.96 | 77.48 | 10.56 | 1.12 |
| 469 | Betamethasone | 89.18 | 1.44 | 1.03 | 66.24 | 86.35 | 0.94 | 91.67 | 3.44 | 0.98 |
| 470 | Benzthiazide | 91.55 | 1.12 | 1.00 | 94.25 | 2.18 | 1.01 | 86.82 | 1.91 | 1.07 |
| 471 | Glipizide | 90.92 | 1.92 | 1.02 | 95.87 | 2.65 | 0.98 | 92.12 | 1.66 | 1.06 |
| 472 | Glimepiride | 86.85 | 2.64 | 1.01 | 96.63 | 3.37 | 0.96 | 85.65 | 0.97 | 1.06 |
| 473 | Glibenclamide | 86.56 | 3.94 | 1.07 | 94.64 | 4.13 | 0.97 | 84.28 | 5.57 | 1.07 |
| 474 | Dipyridamole | 53.49 | 5.62 | 1.10 | 69.99 | 4.14 | 1.01 | 64.00 | 6.04 | 1.27 |
| 475 | Thiabendazole | 104.14 | 4.08 | 1.30 | 108.01 | 4.47 | 1.05 | 95.95 | 4.55 | 1.08 |
| 476 | Levamisole Hydrochloride | 103.01 | 0.90 | 1.09 | 101.37 | 0.84 | 0.98 | 78.14 | 3.36 | 1.19 |
| 477 | 5-hydroxythiabendazole | 96.74 | 5.64 | 1.99 | 109.33 | 4.73 | 1.66 | 69.66 | 9.19 | 2.26 |
| 478 | 4-acetylaminoantipyrine | 75.82 | 14.43 | 0.91 | 102.18 | 1.14 | 1.15 | 80.49 | 1.78 | 1.46 |
| 479 | Albendazole-2-Aminosulfone | 73.83 | 1.82 | 0.95 | 133.96 | 15.39 | 0.63 | 92.42 | 21.34 | 1.06 |
| 480 | sudan 1 | 73.83 | 1.82 | 0.95 | 89.18 | 3.28 | 0.89 | 78.56 | 6.96 | 0.87 |
| 481 | Oxibendazole | 98.05 | 1.98 | 1.08 | 102.80 | 4.16 | 1.04 | 84.10 | 1.23 | 1.21 |
| 482 | Albendazole | 89.65 | 1.49 | 1.17 | 90.30 | 4.97 | 1.19 | 92.29 | 1.34 | 1.22 |
| 483 | Disperse yellow 3 | 82.67 | 2.49 | 0.86 | 87.44 | 3.83 | 1.00 | 85.44 | 1.99 | 1.00 |
| 484 | Tolbutamide | 100.57 | 9.13 | 0.89 | 83.50 | 6.49 | 1.05 | 67.38 | 21.00 | 1.62 |
| 485 | Sudan 2 | 60.75 | 0.42 | 1.00 | 85.25 | 1.93 | 0.91 | 65.48 | 2.16 | 0.98 |
| 486 | Albendazole sulfoxide | 86.21 | 1.01 | 1.17 | 95.81 | 3.35 | 1.32 | 84.61 | 1.31 | 1.44 |
| 487 | N-acetyl dapsone | 91.20 | 4.17 | 0.98 | 66.02 | 85.90 | 0.91 | 93.11 | 1.76 | 0.96 |
| 488 | Fenbendazole sulfone | 86.21 | 5.38 | 1.05 | 87.92 | 5.43 | 1.14 | 83.23 | 6.41 | 1.23 |
| 489 | 5-Hydroxymebendazole | 98.34 | 3.12 | 1.17 | 102.08 | 2.85 | 1.04 | 79.59 | 7.98 | 1.28 |
| 490 | Cambendazole | 98.14 | 2.00 | 1.09 | 94.53 | 3.13 | 1.10 | 81.09 | 2.24 | 1.29 |
| 491 | Oxfendazole | 91.72 | 1.31 | 1.06 | 97.99 | 1.81 | 1.06 | 83.64 | 4.17 | 1.26 |
| 492 | Fenbendazole sulfone | 95.21 | 5.79 | 0.98 | 92.43 | 5.60 | 1.02 | 78.80 | 4.24 | 1.19 |
| 493 | Robenidine Hydrochloride | 61.24 | 9.04 | 1.28 | 104.17 | 6.38 | 0.99 | 72.93 | 3.29 | 1.30 |
| 494 | Sudan blue 2 | 66.72 | 7.92 | 1.04 | 84.15 | 3.91 | 0.97 | 59.49 | 2.39 | 1.28 |
| 495 | Acid yellow 36 | 62.17 | 3.53 | 1.09 | 84.93 | 4.38 | 0.94 | 60.24 | 3.81 | 1.27 |
| 496 | Indometacin | 53.60 | 6.60 | 0.95 | 93.73 | 2.90 | 0.95 | 87.97 | 3.56 | 1.03 |
| 497 | Triclabendazole | 87.34 | 2.68 | 0.95 | 95.53 | 3.51 | 0.77 | 70.00 | 4.66 | 1.05 |
| 498 | Glipizide | 90.92 | 1.92 | 1.02 | 95.87 | 2.65 | 0.98 | 92.12 | 1.66 | 1.06 |
| 499 | Febantel | 89.72 | 3.89 | 0.96 | 96.76 | 5.64 | 0.90 | 79.62 | 1.68 | 1.13 |
| 500 | Glibenclamide | 88.68 | 0.81 | 1.00 | 94.18 | 1.73 | 1.00 | 91.73 | 3.52 | 0.99 |
| 501 | Valnemulin hydrochloride | 86.90 | 2.44 | 1.47 | 93.93 | 3.23 | 1.37 | 83.24 | 14.51 | 1.38 |
| 502 | Phenylethanolamine A | 95.90 | 1.84 | 1.02 | 96.19 | 3.02 | 1.04 | 89.74 | 2.44 | 1.11 |
| 503 | Cimaterol | 107.35 | 1.24 | 1.22 | 92.58 | 3.30 | 1.17 | 82.30 | 6.84 | 1.33 |
| 504 | Terbutaline | 110.72 | 0.55 | 1.17 | 95.00 | 2.56 | 1.24 | 98.92 | 3.54 | 1.24 |
| 505 | Salbutamol | 103.87 | 3.20 | 1.23 | 96.26 | 2.34 | 1.29 | 104.67 | 0.79 | 1.23 |
| 506 | Procaterol | 62.73 | 3.56 | 1.59 | 93.23 | 4.59 | 1.70 | 97.47 | 0.85 | 1.60 |
| 507 | Cimbuterol | 98.11 | 1.06 | 1.06 | 93.36 | 2.81 | 1.08 | 95.53 | 4.85 | 1.06 |
| 508 | Clonidine | 101.32 | 5.64 | 1.10 | 96.85 | 1.42 | 1.04 | 88.86 | 4.28 | 1.11 |
| 509 | Fenoterol | 95.80 | 2.33 | 0.67 | 94.52 | 4.55 | 0.71 | 107.13 | 5.53 | 0.62 |
| 510 | Clenproperol | 103.88 | 1.16 | 1.09 | 93.99 | 2.34 | 1.12 | 92.30 | 2.17 | 1.14 |
| 511 | Clorprenaline | 104.28 | 1.08 | 1.05 | 91.97 | 1.09 | 1.08 | 93.26 | 3.12 | 1.07 |
| 512 | Ractopamine | 102.20 | 2.34 | 1.17 | 95.46 | 1.36 | 1.11 | 87.89 | 4.40 | 1.19 |
| 513 | lsoxsuprine | 103.48 | 2.54 | 1.07 | 94.42 | 1.02 | 1.00 | 80.06 | 7.28 | 1.15 |
| 514 | Clenbuterol | 100.02 | 2.21 | 1.10 | 97.90 | 1.87 | 1.01 | 84.02 | 1.54 | 1.16 |
| 515 | Tulobuterol | 105.30 | 1.91 | 1.03 | 95.55 | 3.11 | 1.02 | 83.03 | 2.85 | 1.16 |
| 516 | Formoterol | 102.67 | 2.35 | 1.49 | 106.61 | 5.77 | 1.18 | 71.34 | 7.69 | 1.72 |
| 517 | Clencyclohexerol | 101.36 | 1.23 | 0.91 | 95.08 | 3.87 | 1.04 | 103.90 | 2.88 | 0.94 |
| 518 | Brombuterol | 98.81 | 2.19 | 1.05 | 90.82 | 1.68 | 1.06 | 92.51 | 2.91 | 1.05 |
| 519 | Clenpenterol | 97.55 | 3.72 | 1.03 | 92.44 | 1.23 | 1.00 | 91.37 | 1.30 | 1.03 |
| 520 | Bambuterol | 103.90 | 1.08 | 1.14 | 99.24 | 1.43 | 0.99 | 74.54 | 5.38 | 1.30 |
| 521 | Clenhexerol | 99.76 | 2.79 | 1.05 | 93.72 | 1.95 | 1.00 | 87.12 | 3.62 | 1.09 |
| 522 | Cyproheptadine | 102.01 | 1.56 | 0.99 | 91.78 | 2.67 | 0.95 | 80.55 | 2.97 | 1.10 |
| 523 | Salmeterol | 100.97 | 5.98 | 1.40 | 114.33 | 4.10 | 1.01 | 75.13 | 6.21 | 1.54 |
| 524 | Amiloride | 77.59 | 11.74 | 1.44 | 102.40 | 5.73 | 1.16 | 82.07 | 8.65 | 1.45 |
| 525 | Trenbolone | 75.38 | 2.34 | 0.94 | 94.27 | 2.80 | 0.92 | 79.91 | 5.86 | 1.15 |
| 526 | Levonorgestrel | 95.36 | 6.57 | 0.98 | 95.78 | 7.22 | 1.05 | 103.85 | 23.20 | 1.03 |
| 527 | Megestrol acetate | 73.91 | 2.42 | 0.86 | 89.00 | 1.96 | 0.88 | 94.42 | 1.61 | 0.79 |
| 528 | Androstendione | 61.93 | 2.66 | 0.93 | 97.30 | 4.17 | 0.84 | 82.57 | 4.34 | 0.96 |
| 529 | Beclomethasone | 89.25 | 6.91 | 0.94 | 98.46 | 4.51 | 0.86 | 77.06 | 12.21 | 1.00 |
| 530 | Danazol | 77.36 | 5.21 | 0.86 | 84.81 | 6.29 | 0.96 | 84.18 | 4.80 | 0.96 |
| 531 | Methylandrostendiol | 60.15 | 3.27 | 0.93 | 93.36 | 6.90 | 1.06 | 123.95 | 7.08 | 0.79 |
| 532 | Betamethasone | 93.41 | 1.44 | 0.99 | 98.99 | 4.00 | 0.94 | 91.67 | 3.44 | 0.98 |
| 533 | Corticosterone | 57.92 | 4.92 | 0.98 | 96.21 | 5.14 | 0.91 | 80.83 | 9.77 | 1.09 |
| 534 | testosterone | 68.44 | 5.58 | 0.82 | 57.67 | 8.17 | 0.87 | 31.25 | 6.34 | 1.05 |
| 535 | Indapamide | 107.10 | 0.63 | 0.88 | 85.51 | 2.44 | 0.97 | 88.49 | 2.58 | 0.98 |
| 536 | Canrenone | 66.63 | 5.42 | 1.04 | 100.07 | 1.15 | 0.82 | 81.03 | 5.93 | 1.01 |
| 537 | Chlorthalidone | 92.95 | 7.57 | 1.60 | 88.55 | 17.09 | 1.75 | 168.71 | 17.21 | 0.71 |
| 538 | Triamterene | 79.76 | 7.41 | 1.69 | 106.52 | 10.47 | 1.16 | 66.07 | 7.12 | 1.73 |
| 539 | Nandrolone Phenylpropionate | 68.43 | 4.03 | 0.93 | 88.67 | 3.02 | 0.90 | 78.11 | 1.40 | 0.92 |
| 540 | Testosterone propionate | 64.71 | 1.92 | 1.00 | 59.51 | 86.35 | 0.96 | 77.86 | 2.70 | 1.04 |
| 541 | Nandrolone 17-propionate | 72.62 | 1.44 | 0.95 | 82.86 | 2.02 | 0.96 | 79.35 | 5.03 | 0.98 |
| 542 | Methyltestosterone | 79.58 | 0.31 | 0.97 | 92.42 | 3.38 | 0.92 | 88.47 | 1.09 | 0.96 |
| 543 | Epiandrosterone | 94.67 | 0.89 | 1.02 | 91.36 | 4.70 | 1.24 | 112.34 | 7.56 | 1.04 |
| 544 | Testosterone | 64.12 | 2.57 | 0.98 | 96.95 | 6.77 | 0.91 | 78.71 | 6.02 | 1.05 |
| 545 | Boldenone | 68.43 | 1.12 | 0.95 | 92.80 | 1.45 | 0.98 | 84.73 | 1.03 | 0.95 |
| 546 | Nandrolone | 72.61 | 7.52 | 0.88 | 89.82 | 2.52 | 0.91 | 89.73 | 1.91 | 0.93 |
| 547 | Medroxyprogesterone | 79.80 | 5.04 | 0.85 | 85.75 | 2.72 | 0.91 | 93.58 | 1.36 | 0.81 |
| 548 | Chlormadinone acetate | 75.50 | 13.09 | 0.83 | 112.34 | 2.78 | 0.65 | 88.69 | 3.73 | 0.90 |
| 549 | Testosterone | 54.98 | 10.99 | 1.09 | 88.62 | 2.05 | 1.03 | 89.63 | 2.06 | 0.98 |
| 550 | Aspirin | 109.90 | 7.54 | 0.89 | 105.87 | 4.24 | 0.93 | 91.79 | 9.14 | 1.08 |
| 551 | Rafoxanide | 76.10 | 19.56 | 0.98 | 98.59 | 18.25 | 0.60 | 59.23 | 13.27 | 0.99 |
| 552 | Doramectin | 86.99 | 14.48 | 0.88 | 94.13 | 10.04 | 0.89 | 84.75 | 2.72 | 0.99 |

**References**

[1] K. Sdougkou, H. Xie, S. Papazian, et al., "Phospholipid Removal for Enhanced Chemical Exposomics in Human Plasma", *Environmental Science & Technology* 57(28) (2023) 10173-10184. <https://doi.org/10.1021/acs.est.3c00663>.

[2] J. Carmical, S. Brown, "The impact of phospholipids and phospholipid removal on bioanalytical method performance", *Biomedical Chromatography* 30(5) (2016) 710-720. <https://doi.org/https://doi.org/10.1002/bmc.3686>.

[3] J. Godzien, M. Ciborowski, M.P. Martínez-Alcázar, et al., "Rapid and Reliable Identification of Phospholipids for Untargeted Metabolomics with LC–ESI–QTOF–MS/MS", *Journal of Proteome Research* 14(8) (2015) 3204-3216. <https://doi.org/10.1021/acs.jproteome.5b00169>.

[4] M.C. Sullards, J.C. Allegood, S. Kelly, et al., Structure‐Specific, Quantitative Methods for Analysis of Sphingolipids by Liquid Chromatography–Tandem Mass Spectrometry: “Inside‐Out” Sphingolipidomics, Methods in Enzymology, Academic Press2007, pp. 83-115. <https://doi.org/https://doi.org/10.1016/S0076-6879(07)32004-1>.
